# A computationally designed binding mode flip leads to a novel class of potent tri-vector cyclophilin inhibitors†
†Electronic supplementary information (ESI) available: Chemical synthesis and characterization of novel compounds. Computational, biochemical, biophysical and cellular assays protocols. The crystal structures of **2**, **4**, **5**, **7**, **8**, **9**, **10**, **15** in complex with CypA have been deposited in the Protein Data Bank (PDB codes 6GJJ, 6GJM, 6GJY, 6GJP, 6GJI, 6GJR, 6GJL, 6GJN respectively). See DOI: 10.1039/c8sc03831g


**DOI:** 10.1039/c8sc03831g

**Published:** 2018-10-23

**Authors:** Alessio De Simone, Charis Georgiou, Harris Ioannidis, Arun A. Gupta, Jordi Juárez-Jiménez, Dahlia Doughty-Shenton, Elizabeth A. Blackburn, Martin A. Wear, Jonathan P. Richards, Paul N. Barlow, Neil Carragher, Malcolm D. Walkinshaw, Alison N. Hulme, Julien Michel

**Affiliations:** a University of Edinburgh , Joseph Black Building, King's Buildings, David Brewster Road , Edinburgh , Scotland EH9 3FJ , UK . Email: mail@julienmichel.net; b Edinburgh Phenotypic Assay Centre , University of Edinburgh , Queen's Medical Research Institute , Little France Cres , Edinburgh , Scotland EH16 4TJ , UK; c The Edinburgh Protein Production Facility (EPPF) , University of Edinburgh , Level 3 Michael Swann Building, King's Buildings, Max Born Crescent , Edinburgh , Scotland EH9 3BF , UK; d Cancer Research UK Edinburgh Centre , University of Edinburgh , MRC Institute of Genetics and Molecular Medicine , Crewe Road South , Edinburgh , Scotland EH4 2XR , UK; e University of Edinburgh , Michael Swann Building, Max Born Crescent , Edinburgh , Scotland EH9 3BF , UK

## Abstract

Cyclophilins (Cyps) are a major family of drug targets that are challenging to prosecute with small molecules because the shallow nature and high degree of conservation of the active site across human isoforms offers limited opportunities for potent and selective inhibition. Herein a computational approach based on molecular dynamics simulations and free energy calculations was combined with biophysical assays and X-ray crystallography to explore a flip in the binding mode of a reported urea-based Cyp inhibitor. This approach enabled access to a distal pocket that is poorly conserved among key Cyp isoforms, and led to the discovery of a new family of sub-micromolar cell-active inhibitors that offer unprecedented opportunities for the development of next-generation drug therapies based on Cyp inhibition. The computational approach is applicable to a broad range of organic functional groups and could prove widely enabling in molecular design.

## Abstract 2

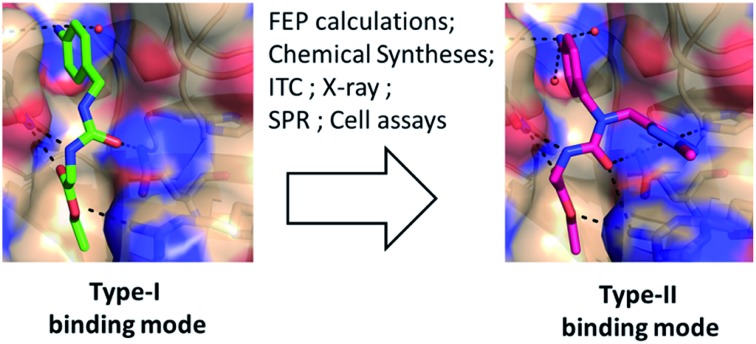
Molecular simulations led to the discovery of a new class of small molecules that inhibit the cyclophilin family of proteins.

## Introduction

In the absence of innovative treatments, the societal burden of neurodegenerative disorders is projected to grow substantially over the next few decades worldwide. A growing body of evidence points to the central role of the opening of the mitochondrial permeability transition pore (mPTP) in neurodegenerative cell death.^1^ The enzyme cyclophilin D (CypD) is an essential regulator of the mPTP opening, and blockage of CypD function is neuroprotective in models of Alzheimer's and Parkinson's diseases.^2^–^4^ Besides neurodegenerative disorders, CypD inhibition is protective in animal models of acute kidney injury, a condition that accounts for a fifth of emergency hospitalizations in the USA.^5^ Other related cyclophilin isoforms CypA and CypB are overexpressed in malignant cancers,^6^ and CypA inhibition has potent antiviral and anti-inflammatory effects.^7^ Cyp inhibition may also protect the liver from fibrosis subsequent to non-alcoholic steatohepatitis (NASH), a common indication for liver transplantation after chronic hepatitis C.^8^

**Fig. 1:**
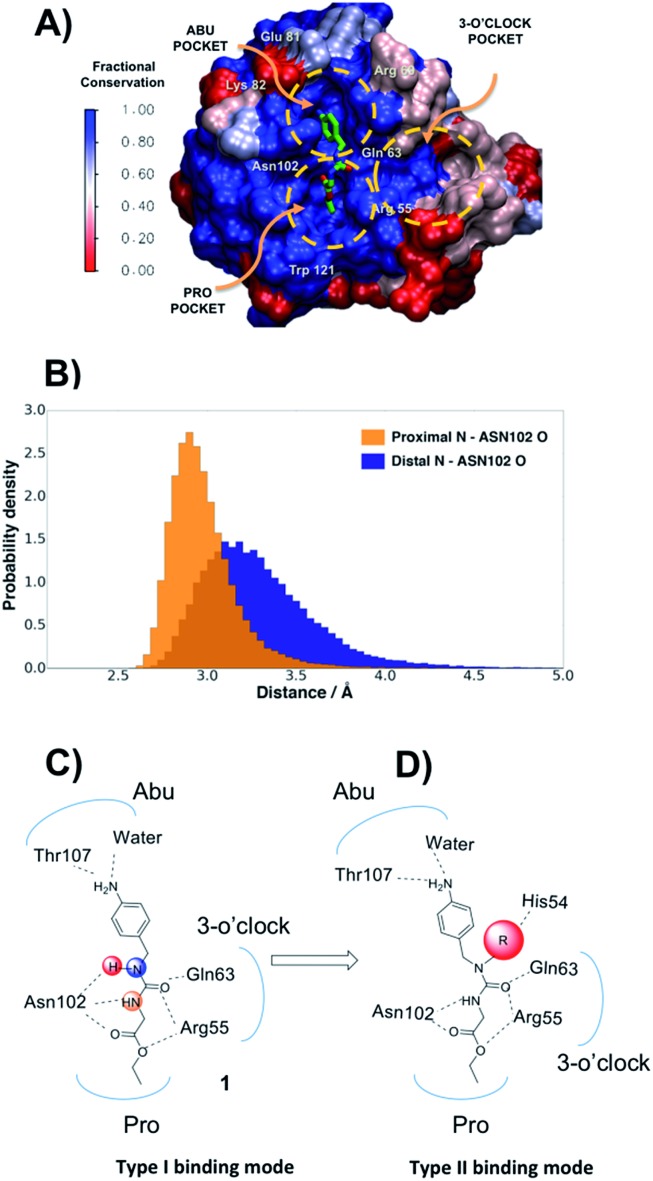
(A) Surface representation of the PPIAse domain coloured by residue conservation across human Cyp A–D isoforms. The location of Abu, Pro and 3 o'clock pockets are highlighted and compound **1** is shown in coloured sticks. (B) Distribution of distances between urea nitrogen atoms in **1** and Asn102 backbone oxygen observed in MD simulations. (C) Interactions of **1** with CypA residues in a canonical type-I binding mode. (D) Hypothesized type-II binding mode for alkylated urea variants of **1**.

The majority of existing Cyp inhibitors are derived from the natural products cyclosporin A (CsA) and sanglifehrin A.^9^ These scaffolds achieve potent inhibition but are complex to synthesize, lack sub-type selectivity, show undesirable side-effects, and have poor CNS activity. Extensive efforts have been pursued to simplify these scaffolds into more synthetically tractable variants.^10^–^13^ There is also considerable interest in developing small molecule Cyp inhibitors that may be more easily optimized for use as therapeutics.^14^–^20^ However, there are few reports of well characterized small molecules that bind strongly to Cyps.^21^,^22^ Current small molecule design strategies typically feature linear (thio)ureas^15^,^23^ or amides^24^ as scaffolds. Compound **1** provides an example that exploits interactions with residues in the highly conserved Pro and Abu pockets (Fig. 1A). Fig. 1A also highlights a less conserved distal three o'clock pocket that is thought to offer prospects for potency and selectivity improvements,^25^ but to date it is unclear how this pocket can be engaged using current scaffolds.

Herein we report a multidisciplinary approach to redesigning linear Cyp inhibitor scaffolds into the first class of tri-vector inhibitors that simultaneously target the Pro, Abu and three o'clock pockets.

## Results and discussion

### Molecular dynamics simulations suggest a new ligand optimisation strategy

The protein X-ray structure of **1** in complex with CypA suggests that both urea nitrogen atoms are hydrogen-bonded to the backbone oxygen of Asn102.^21^ However, molecular dynamics (MD) simulations revealed that the nitrogen atom distal to the ester moiety in **1** is only weakly interacting with Asn102 and readily moves away from this residue (Fig. 1B, C and S1†), suggesting that alkylation of that nitrogen to introduce a new vector in the scaffold could be tolerated. *Ab initio* calculations on model ureas suggest a modest energetic preference for the *Z*,*Z* urea conformer.^26^ This led to the hypothesis that a suitably chosen R group could stabilize an alkylated urea in an *E*,*Z* conformer able to adopt a novel type-II binding mode that would enable access to the 3 o'clock pocket (Fig. 1D).

### A general synthetic route to tri-vector cyclophilin ligands

**Scheme 1:**
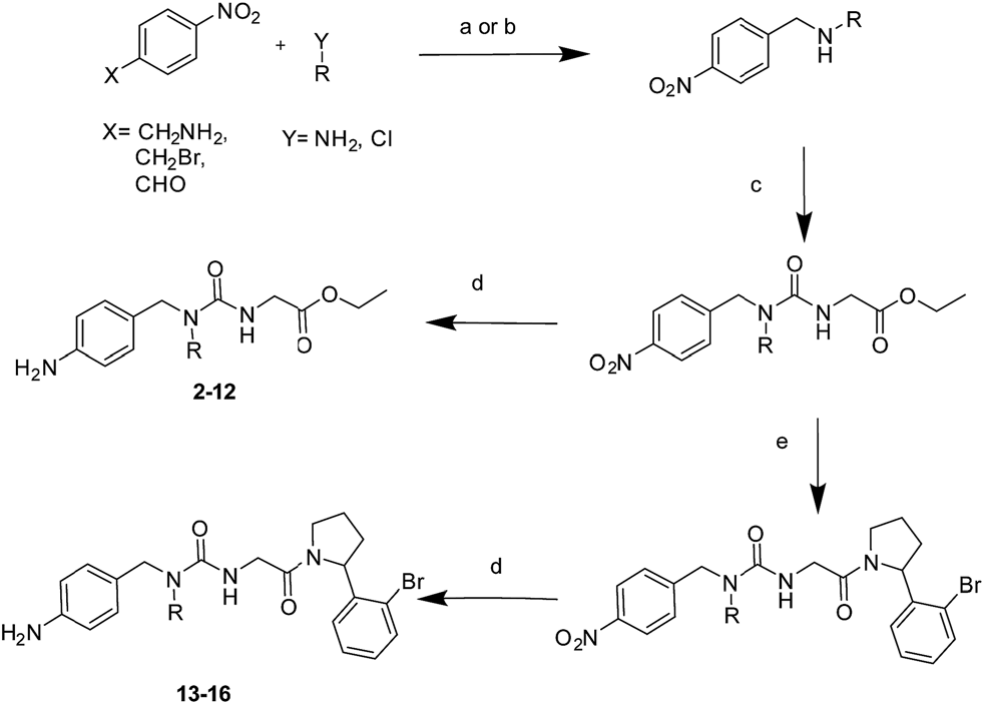
(a) NaBH_4_, EtOH, RT, 24 hours, yields: 93–95%; (b) K_2_CO_3_, MeCN, 70 °C, 6 hours, yields: 95–99%; (c) ethyl isocyanatoacetate, DCM, RT, overnight, yields: 81–99%; (d) Fe, CaCl_2_, EtOH/H_2_O, 90 °C, 4 hours, yields: 17–98%. (e) LiOH·H_2_O, THF/MeOH/H_2_O, RT, 2 hours, then 2-(2-bromophenyl)-pyrrolidine, HATU, DIPEA, DMF, RT, overnight, yields: 9–46% over two steps.

The tri-vector design hypothesis was pursued by synthesis of a series of alkylated urea ester ligands. The majority of the compounds were prepared according to Scheme 1 (steps a–d, see ESI† for details). Substituted amino derivatives were typically prepared from 4-nitrophenyl derivatives by reductive amination,^27^ or nucleophilic substitution.^28^ The urea moiety was prepared by condensation of the resulting amines with ethyl isocyanatoacetate,^15^ and the desired products were obtained by subsequent reduction of the nitro group.^29^

### A free-energy perturbation (FEP) protocol guides the selection of substituents

**Fig. 2:**
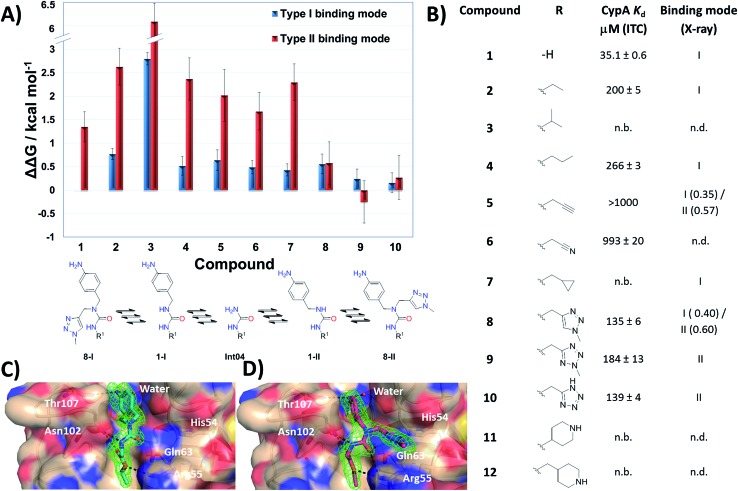
(A) FEP-calculated binding energetics. The figures are relative to **1** in a type-I binding mode and were obtained from automated analysis of FEP maps (Fig. S2†). A partial map depicted below the table illustrates how molecule Int04 is used to connect other compounds in a type-I or II binding mode. (B) ITC-derived dissociation constants and observed binding mode in X-ray crystallography derived CypA structures. Uncertainties on *K*_d_ values from fit to a one-site binding model. Partial occupancies are given for compounds refined in multiple binding modes. n.b.: no binding observed at the maximum concentration tested. n.d.: not determined. (C) X-ray crystal structure of CypA: **2** depicting a type-I binding mode. (D) X-ray crystal structure of CypA: **9** depicting a type-II binding mode. Fo – Fc electron density omit maps are shown as a green mesh at 2.5*σ* contour (see ESI† for details).

MD simulations cannot readily sample the desired type-I to type-II binding mode flip because rotational barriers of *ca.* 15 kcal mol^–1^ separate *Z*,*Z* and *E*,*Z* conformers.^30^ Free Energy Perturbation (FEP) calculation methods have shown potential for optimization of ligand potency and selectivity.^31^–^38^ Here the selection of potential R groups was guided by a novel FEP protocol designed to compute the energetics of this binding mode flip (Fig. 2A). The setup, execution and analysis of the FEP calculations were carried out with the software FESetup,^39^ SOMD,^40^–^42^ and freenrgworkflows.^43^ The selection of R groups for computational and experimental evaluation was driven by the following considerations: lack of obvious steric or electrostatic mismatches, polar or non-polar interactions with 3 o'clock pocket residues, ease of synthesis, diversity of structure–activity relationships, and validation of FEP-calculated energetics.

Compound **1** was predicted to favour a type-I binding mode by *ca.* 1 kcal mol^–1^, in line with the preferences observed in X-ray structures (Fig. 2A).^21^ Non-polar substitutions (**2**, **3**, **4**, **5**, **7**) were predicted to worsen free energies of binding relative to **1** and maintain preference for a type-I binding mode. By contrast nitrogen-rich 5-membered rings (**8**, **9**, **10**) were predicted to show a similar or slight preference for type-II binding mode.

Ligand binding to CypA was characterized by isothermal titration calorimetry (ITC) measurements (Fig. 2B). Substitutions with small linear alkyl groups (**2**, **4**) were tolerated but weakened binding, whereas branched alkyls (**3**) were inactive, in line with the FEP calculations. Alkyne, cyano and cyclopropyl derivatives (**5**, **6**, **7**) were computed to bind with potencies comparable to linear alkyl groups, but turned out to be very weak binders in the ITC assay. Nitrogen rich five-membered rings (**8**, **9**, **10**) showed more favourable binding constants, in line with the FEP calculations. Piperidines **11** and **12** had not been evaluated by FEP calculations and turned out to be inactive.

Evidence of binding mode preferences was sought by soaking CypA crystals with compounds **2–12**. Seven X-ray structures of CypA complexes were determined. Compounds **2**, **4**, **7** were found to adopt a type-I binding mode in line with the FEP calculations (Fig. 2C and ESI†). Compound **5** was observed to adopt dual occupancy, whereas the FEP calculations anticipated a type-I binding mode. Compound **8** was observed to adopt dual occupancy, whereas **9** and **10** were only observed in a type-II binding mode in the X-ray refined structures. The FEP calculations suggest both binding modes are feasible for these three compounds. In **8–10** the type-II binding mode is stabilized by hydrogen-bonding interactions between nitrogen atoms in the five-membered rings and His54. This binding mode flip from type-I to type-II is accompanied by a slight rearrangement in the orientation of the urea carbonyl to maintain optimal placement of the aniline ring in the Abu pocket. Strikingly the 5-membered ring methyl groups in **8** and **9** point into the 3 o'clock pocket of CypA and provide a clear vector for further derivatization (Fig. 2D).

### The tri-vector design generalises to other ligand families and Cyp isoforms

**Fig. 3:**
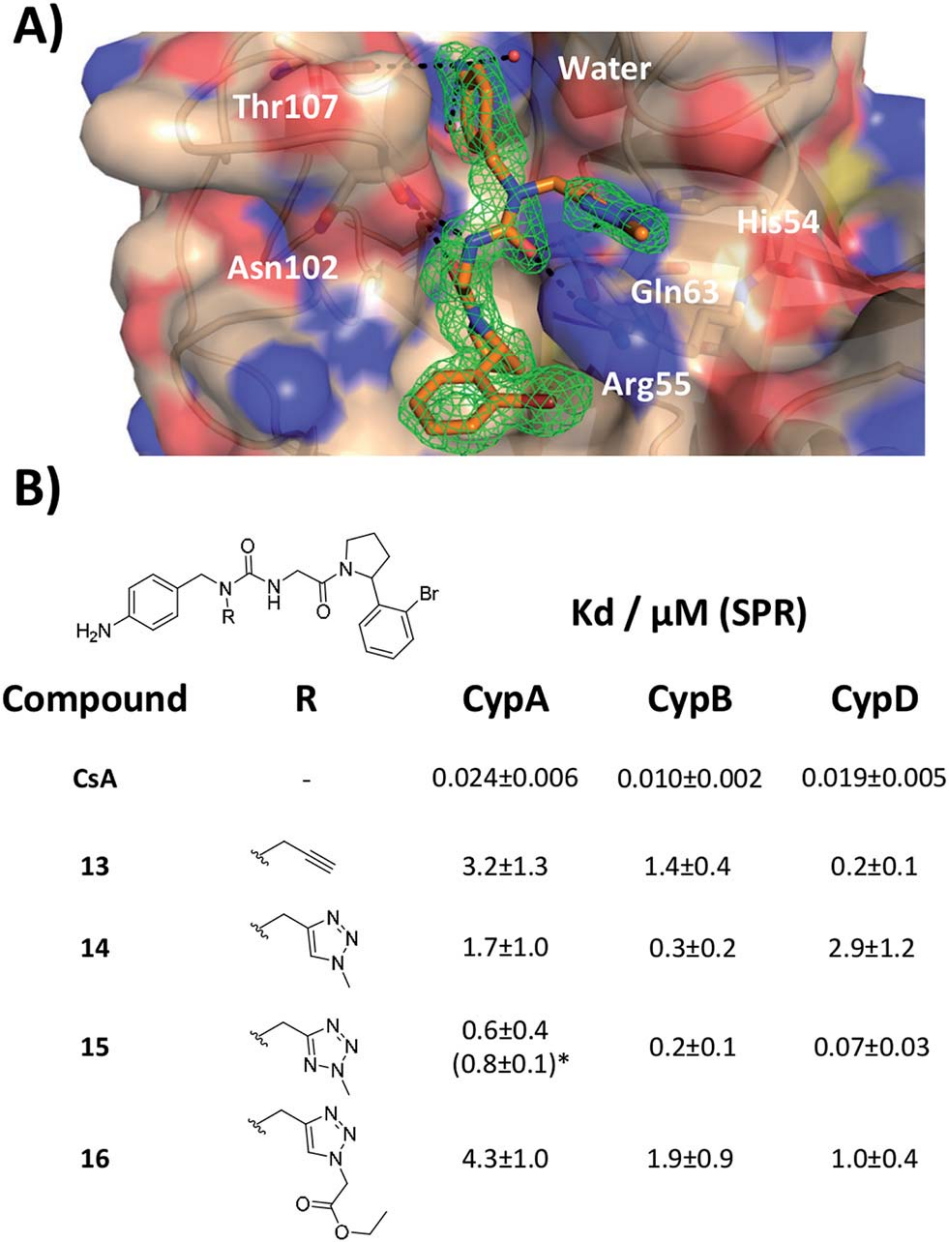
(A) X-ray crystal structure of CypA in complex with **15**. (B) SPR-derived dissociation constants for alkylated urea arylpyrrolidine ligands. Values are the mean ± 1*σ* from three repeat experiments. * ITC-derived *K*_d_ value (see ESI† for details).

With the binding mode flip hypothesis validated for the urea ester series in CypA, attention turned to transferability of the strategy to other ligand families and Cyp isoforms. FEP calculations suggested that a type-II binding mode preference was maintained for nitrogen-rich heterocycles in the context of a urea arylpyrrolidine scaffold for both CypA and CypD isoforms (Fig. S3–S5†).^21^ Thus alkylated variants **13–16** were prepared from 4-nitrophenyl ester intermediates (Scheme 1, steps e and d). Compounds **13–15** feature similar R groups to **5**, **8** and **9**, whereas **16** was prepared to assess viability of further extension into the three o'clock pocket *via* appendage of an ester group to a triazole. The X-ray crystallography derived structure of **15** in complex with CypA confirmed that the type-II binding mode has been maintained (Fig. 3A). Thus **13–16** were assayed by surface plasmon resonance (SPR) against CypA, CypB and CypD isoforms. The compounds bind to all three isoforms with *K*_d_ values in the low μM to mid nM range (Fig. 3B). Compound **15** binds particularly well to CypD, with a potency (*ca.* 70 nM) approaching that of CsA (*ca.* 20 nM). For comparison the unsubstituted (R = H) urea has been reported to inhibit CypD with an IC_50_ of 1.1 ± 0.2 μM.^15^ Although **13–15** only access the entrance of the three o'clock pocket, the SPR assay suggests this is sufficient to endow a degree of isoform selectivity. Compound **16** binds slightly worse to CypA and CypB but modestly better to CypD with respect to **14**. Thus further lead optimization efforts to tune desired selectivity profiles appear auspicious.

### Tri-vector ligands show improved efficacy and decreased toxicity over cyclosporine in cell assays

**Fig. 4:**
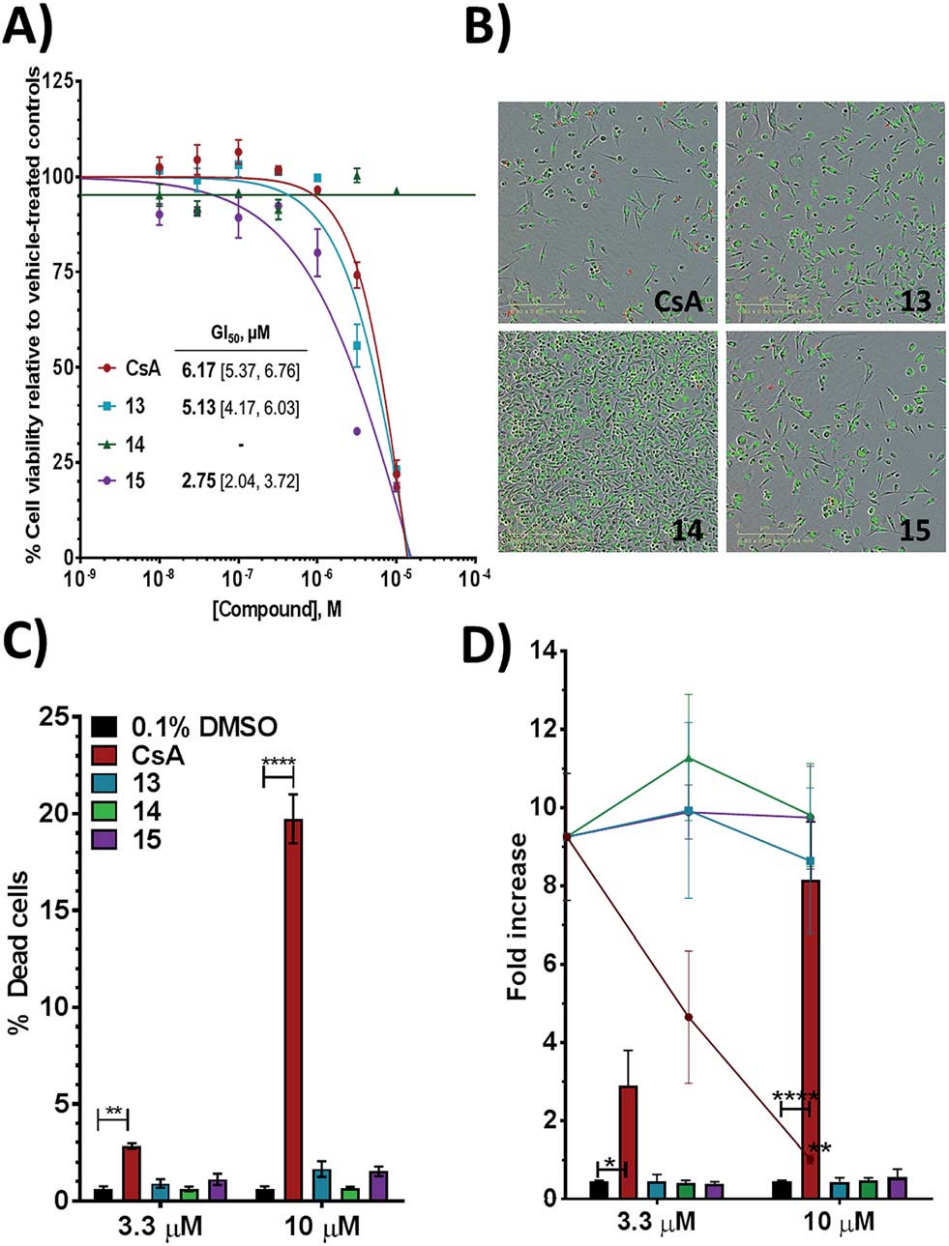
(A) Growth inhibition for MDA-MB-231_NLG cells and mean GI_50_ with 95% CI [lower limit, upper limit]. (B) MDA-MB-231_NLG cells after 120 hours treatment; red nuclei indicate dead cells. Area shown is 0.64 mm^2^, scale bar is 200 μm. (C) % dead MDA-MB-231_NLG cells. (D) Fold increase of IMR-90 cell confluence (line graphs) and number of dead IMR-90 cells (bar graphs). Values in panels A, C, D represent mean ± SEM of three independent experiments each performed in triplicate with 120 hours of treatment.

Additional evidence for the potential of this tri-vector design was sought by evaluating **13–15** in cellular assays. CypA has previously been shown to be necessary for the prolactin-induced activation of Janus-activated kinase 2 in human breast cancer cells, with CsA treatment causing inhibition of growth of the triple negative MDA-MB-231 breast-cancer cell line.^44^ Compounds **13** and **15** showed evidence of dose-dependent growth inhibition at low micromolar concentration resulting in GI_50_ values similar (**13**) or two-fold better (**15**) than those measured for CsA. By contrast **14** was inactive (Fig. 4A and B). Thus alkylation of the urea moiety does not appear to prevent cell penetration. Negligible cell death was observed for compounds **13** and **15** suggesting low toxicity in comparison with CsA. This indicates that in MDA-MB-231_NLG cells growth inhibition by compounds **13** and **15** is due to reduced proliferation, whereas growth inhibition by CsA is also a consequence of cell death (Fig. 4C). Additionally **13–15** showed no growth inhibition or induction of cell death in the non-tumorigenic fibroblast IMR-90 cell line. By contrast CsA induced significant growth inhibition and cell death (Fig. 4D).

Thus, further evaluation of the present tri-vector Cyp inhibitors in a range of *in vitro* and *in vivo* disease models implicating Cyps seems to be warranted, particularly in instances where CsA toxicity and off-target effects have been a cause for concern. Alternatives to the aniline moiety in **15** to alleviate toxicity concerns have been suggested elsewhere.^21^,^45^ Immunosuppression *via* inhibition of calcineurin phosphatase activity is a well-known feature of CsA. The tri-vector Cyp inhibitors are unlikely to possess this capability given that the parent urea-arylpyrrolidine compounds they are derived from lack calcineurin inhibition properties.^21^

## Conclusions

In summary, this work has validated a strategy for the redesign of existing cyclophilin inhibitors into a novel class of inhibitors that can simultaneously target three pockets on the surfaces of cyclophilins. This breakthrough has led to significantly more potent cyclophilin inhibitors and also opens up new horizons to optimise isoform-selectivity. Nanomolar acylurea-based cyclophilin inhibitors have been reported previously,^46^ but subsequent work was unable to reproduce these findings or to detect evidence of binding to cyclophilins for these molecules.^21^ Members of the tri-vector class of cyclophilin inhibitors (**13**, **15**) show improved efficacy and reduced toxicity in cell assays over the drug cyclosporine A. They are thus already attractive leads for new drug therapies based on Cyp inhibition. To our knowledge this is the first example of the validation of an FEP methodology to computationally design a large scale binding mode flip in a protein–ligand complex. The approach is readily applicable to a broad range of commonly encountered organic functional groups that exhibit *E*/*Z* conformational equilibria. We anticipate that this methodology may be of widespread utility to facilitate ligand optimisations in structure-based drug design efforts.

## Conflicts of interest

There are no conflicts to declare.

## Supplementary Material

Supplementary information

Supplementary information
