# Supplementary material for: A computationally designed binding mode flip leads to a novel class of potent tri-vector cyclophilin inhibitors
Source: Chem Sci. 2018 Oct 23;10(2):542–7. doi: 10.1039/c8sc03831g (PMC6335623; doi:10.1039/c8sc03831g)
Supplement: Supplementary file 2 [file SC-010-C8SC03831G-s002.pdf]

Electronic Supplementary Material (ESI) for Chemical Science.
This journal is © The Royal Society of Chemistry 2018

A Computationally Designed Binding Mode Flip Leads to a Novel Class of Potent tri-vector Cyclophilin Inhibitors

Alessio De Simone, Charis Georgiou, Harris Ioannidis, Arun A. Gupta, Jordi Juárez-Jiménez, Dahlia Doughty-Shenton, Elizabeth A. Blackburn, Martin A. Wear , Jonathan P. Richards, Paul N. Barlow, Neil Carragher, Malcolm D. Walkinshaw, Alison N. Hulme, Julien Michel*

Electronic Supplementary Information

Table of Contents

Chemistry.. 2

Chemicals, materials, and methods. 2

General Procedure 1 for the synthesis of the N-substituted 4-nitrobenzylamine derivatives (Steps a and b, Scheme 1 in main text) 3

General Procedure 2 for the synthesis of urea derivatives (Step c, Scheme 1 in main text) 3

General Procedure 3 for the synthesis of the amide derivative (Step e, Scheme 1 in main text).................4

General Procedure 4 for the synthesis of the final aniline derivatives (Step d, Scheme 1 in main text) 4

Synthesis of the intermediates..5

Synthesis of final products 13

Computational Methods 21

System preparation 21

Molecular dynamics simulations..22

Free energy calculations 23
Protein Expression and Purification 31
PEP1 - HisCypA and His-CypD transformation and expression 31
PEP2 - Protein Purification 32
PEP3 – HisTag cleavage 32
PEP4 – Miscellaneous 34
Isothermal Titration Calorimetry (ITC) 34
ITC1 – Ester series: Instrument and experimental setup 34
ITC2 – Bromo-aryl-pyrrolidine series: Instrument and experimental setup 36
Surface Plasmon Resonance (SPR) 37
SPR1 – SPR equipment and reagents 37
SPR2 – Immobilization and covalent stabilizations of His-Cyps 37
SPR3 – Kinetic titration experiments 38
X-ray diffraction experiments 41
Cell assays 43
Materials and Reagents 43
Cell Culture 44
Image-based Cell Viability Assays 44
Western Blotting 45
^{1}H NMR and ^{13}C NMR of final products 47
References 63

Chemistry

Chemicals, materials, and methods.

Abbreviations used in the description of the examples that follow are: Acetonitrile (MeCN); ammonium chloride (NH_4Cl); BnBr (benzyl bromide); carbonyldiimidazole (CDI); caesium carbonate (Cs_2CO_3); cyclohexane (Cy); chloroform ($CHCl_3$); deuterated dimethylsulfoxide (DMSO-*d6*); dichloromethane (DCM); dimethylsulfoxide (DMSO); *N*,*N*-diisopropylethylamine (DIPEA); dimethylformamide (DMF); di-tert-butyldicarbonate (Boc_2O); 4-(dimethylamino)-pyridine (DMAP); ethylene glycol monomethyl ether (EGME); ethanol (EtOH); electrospray ionization (ESI); ethyl acetate (EtOAc); hydrochloric acid (HCl); mass spectrometry (MS); microwave (MW); sulfuric acid (H_2SO_4); iodomethane (MeI); *N*,*N*-dimethylformamide (DMF); lithium hydroxide (LiOH); magnesium sulfate ($MgSO_4$); methanol (MeOH); nuclear magnetic resonance (NMR); room temperature (RT); palladium acetate ($Pd(OAc)_2$); potassium carbonate (K_2CO_3); sodium bicarbonate ($NaHCO_3$); sodium borohydride ($NaBH_4$); tetrabutylammonium iodide (TBAI); triethylsilane (TES); tetrahydrofuran (THF); thin layer chromatography (TLC); triethylamine (Et_3N or TEA) and trifluoroacetic acid (TFA).

Automated column chromatography purifications were conducted using Biotage Isolera One apparatus with prepacked silica gel columns of different sizes (10 and 25 g). Mixtures of increasing polarity of cyclohexane and ethyl acetate or dichloromethane and methanol were used as eluents. Microwave heating was performed using Biotage Initiator instrument. Nuclear magnetic resonance (NMR) spectra were recorded at ambient temperature (298 K, unless otherwise stated) on a Bruker AVA400, AVA500 or AVA600 spectrometer running at 400, 500, or 601 MHz (^{1}H spectra) or 101, 126, 151 MHz (^{13}C spectra, respectively). Chemical shifts (δ values) are reported in parts-per-million (ppm) relative to tetramethylsilane (^{1}H and ^{13}C spectra; δTMS = 0 ppm) and are calibrated to the residual solvent peak. The peaks reported correspond to the principal rotamer. Mass spectra were obtained by electrospray ionization (ESI) on a Bruker microTOF II mass spectrometer. Mass-to-charge ratios (*m/z*) of all parent (molecular) ions ($[M]^{+/-}$) and their intensities are reported, followed by (major) fragment or adduct ions and their intensities. LC-MS analyses were run on a Bruker microTOF II system equipped with an electrospray ionization interface and a photodiode array detector. PDA range was 210−400 nm. Electrospray ionization was applied in positive modes. UPLC mobile

phases were (A) H_2O/Formic Acid (99.9:0.1), and (B) MeCN/Formic acid (99.9:0.1). Analyses were performed with the method reported below. Gradient: 5−100% B over 10 min. Flow rate: 200 μL/min. The purity of all final compounds was determined to be ≥95%. Temperature: 30 °C. Column: Phenomenex Kinetex C18 (5 μm, 2.1 mm × 50 mm). Melting points (mp) were determined on a Gallenkamp Electrothermal Melting Point apparatus.

General Procedure 1 for the synthesis of the N-substituted 4-nitrobenzylamine derivatives (Steps a and b, Scheme 1 in main text)

Method A (Reductive amination).

To a solution of 4-nitrobenzaldehyde (1 eq.) in dry ethanol was added the amine derivative (1.1 eq.) under a nitrogen atmosphere. The solution was stirred at RT for 16 hours and then cooled to 0 °C, and $NaBH_4$ (2 eq.) was added portion-wise until the intermediate imine disappeared (TLC analysis, approximately 8 hours). The reaction mixture was concentrated *in vacuo* and the residue dissolved in DCM. The organic phase was washed sequentially with a saturated solution of $NaHCO_3$, water and brine, dried over $MgSO_4$, filtered, and concentrated *in vacuo* to yield the title compound as a pale-yellow oil, which was used directly in the next step.

Method B (Nucleophilic substitution).

A mixture of 4-nitrobenzyl bromide or 4-nitrobenzylamine hydrochloride (1 eq.), the alkyl amine or alkyl halide (1 eq.), and K_2CO_3 (2 eq.) in acetonitrile was stirred at 70 °C for 6 hours. The suspension was filtered and the residue washed with acetone several times. The combined filtrates were concentrated *in vacuo* to give the desired intermediate as an oil, which was used directly in the next step.

General Procedure 2 for the synthesis of urea derivatives (Step c, Scheme 1 in main text)

To ethyl isocyanatoacetate (1 eq.) dissolved in DCM was added the 4-nitrophenylamino intermediate (1 eq.) and the reaction mixture was stirred at RT overnight. After this time, water was added to the mixture and the organic phase was collected. The aqueous phase was back-extracted with DCM several times and the combined organic phases were dried over magnesium sulfate and evaporated and concentrated *in vacuo*, yielding the desired product as an oil.

General Procedure 3 for the synthesis of the amide derivative (Step e, Scheme 1 in main text)

The ethyl ester derivative (1 eq.) was dissolved in THF/MeOH (1:1) and treated with LiOH monohydrate (5 eq.) dissolved in water (Water/THF/MeOH ratio: 1:2:2). The reaction was stirred at RT for 2 hours, then the reaction mixture was concentrated, diluted with water and washed with DCM. The aqueous phase was treated with HCl (1 N aq.) until an acidic pH was reached and then extracted several times with EtOAc. The combined organic phases were dried over magnesium sulphate and concentrated *in vacuo* to afford the desired products, which were used directly in the next step.

2-(2-Bromophenyl)-pyrrolidine (1 eq.), the carboxylic acid derivative (1.1 eq.) and HATU (1.4 eq.) were dissolved in DMF. DIPEA (1.5 eq.) was added and the reaction mixture was stirred at RT for 18 hours. After this time, the reaction mixture was diluted with EtOAc and washed with water and brine. The organic layer was dried over $MgSO_4$, filtered and concentrated *in vacuo*. The crude product was purified by flash column chromatography eluting with MeOH in DCM. The desired compound was obtained as an oil.

General Procedure 4 for the synthesis of the final aniline derivatives (Step d, Scheme 1 in main text)

The 4-nitrophenylurea intermediate (1 eq.) was placed in a round-bottomed flask and ethanol was added. Fe powder (3 eq.) was added and the reaction heated to reflux temperature (90 °C) at which time water (EtOH/water ratio: 10:2) was added through the top of the condenser together with calcium chloride (1 eq.). After 4 hours the reaction was allowed to cool and filtered through Celite. The reaction solvent was removed *in vacuo*, yielding a crude mixture which was dissolved in DCM and washed with water. The organic phase was collected, dried over magnesium sulfate, filtered and concentrated, yielding the crude product as a yellow oil. The crude product was purified by column chromatography on silica gel (DCM/MeOH 9:1). The desired compound was obtained as an oil.

Synthesis of the intermediates

Ethyl[(4-nitrophenyl)methyl]amine

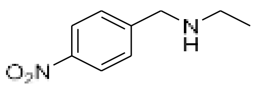

The title compound was synthesized applying the general procedure 1 method A using 4-nitrobenzaldehyde (500 mg, 3.31 mmol), dry ethanol (10 mL), ethylamine (2 M solution in THF, 1.80 mL, 3.64 mmol) and $NaBH_4$ (250 mg, 6.62 mmol). Yellow oil 555 mg (93%). LC-MS: Rt 2.8 min; *m/z* 181 $[M+H]^+$. ^1H NMR (601 MHz, DMSO-*d6*) δ 8.19 – 8.16 (m, 2H), 7.63 – 7.59 (m, 2H), 3.81 (s, 2H), 2.51 (q, *J* = 7.0 Hz, 2H), 2.21 (bs, 1H), 1.03 (t, *J* = 7.1 Hz, 3H).

[(4-Nitrophenyl)methyl](propan-2-yl)amine

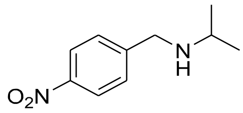

The title compound was synthesized applying the general procedure 1 method A using 4-nitrobenzaldehyde (500 mg, 3.31 mmol), dry ethanol (10 mL), isopropylamine (0.31 mL, 3.64 mmol) and $NaBH_4$ (250 mg, 6.62 mmol). Yellow oil 600 mg (93%). LC-MS: Rt 1.3 min; *m/z* 195 $[M+H]^+$. ^1H NMR (500 MHz, DMSO-d_6) δ 8.20 – 8.15 (m, 2H), 7.65 – 7.60 (m, 2H), 3.82 (s, 2H), 2.69 (qn, *J* = 6.2 Hz, 1H), 2.14 (s, 1H), 1.00 (d, *J* = 6.3 Hz, 6H).

(Cyclopropylmethyl)[(4-nitrophenyl)methyl]amine

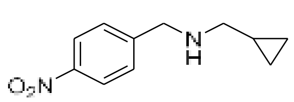

The title compound was synthesized applying the general procedure 1 method A using 4-nitrobenzaldehyde (620 mg, 4.10 mmol), dry ethanol (10 mL), cyclopropylmethylamine (0.40 mL, 4.51 mmol) and $NaBH_4$ (310 mg, 8.21 mmol). Yellow oil 800 mg (95%). LC-MS: Rt 2.9 min; *m/z* 207 $[M+H]^+$. ^1H NMR (500 MHz, DMSO-d_6) δ 8.20 – 8.15 (m, 2H), 7.64 – 7.60 (m, 2H), 3.84 (s, 2H), 2.37 (d, *J* = 6.7 Hz, 2H), 2.33 (s, 1H), 0.93 – 0.84 (m, 1H), 0.41 – 0.37 (m, 2H), 0.10 – 0.06 (m, 2H).

***tert*-Butyl 4-{[(4-nitrophenyl)methyl]amino}piperidine-1-carboxylate**

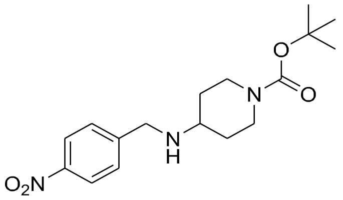

The title compound was synthesized applying the general procedure 1 method A using 4-nitrobenzaldehyde (300 mg, 1.99 mmol), dry ethanol

(10 mL), 4-Amino-1-boc-piperidine (438 mg, 2.19 mmol) and $NaBH_4$ (150 mg, 3.97 mmol). Yellow oil 650 mg (98%). LC-MS: Rt 5.2 min; *m/z* 280 [M –t-butyl+H]$^+$. ^{1}H NMR (500 MHz, DMSO-d_6) δ 8.20 – 8.16 (m, 2H), 7.66 – 7.61 (m, 2H), 3.86 (s, 2H), 3.81 (bs, 2H), 2.79 (bs, 2H), 2.41 – 2.27 (m, 1H), 1.78 (dt, *J* = 12.9, 3.6 Hz, 2H), 1.74 – 1.50 (m, 1H), 1.39 (s, 9H), 1.15 (dddd, *J* = 12.9, 11.1, 9.7, 4.2 Hz, 2H).

***tert*-Butyl 4-({[(4-nitrophenyl)methyl]amino}methyl)piperidine-1-carboxylate**

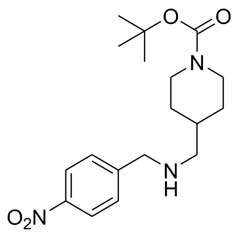

The title compound was synthesized applying the general procedure 1 method A using 4-nitrobenzaldehyde (300 mg, 1.99 mmol), dry ethanol (10 mL), 4-Amino-1-boc-piperidine (469 mg, 2.19 mmol) and $NaBH_4$ (150 mg, 3.97 mmol). Yellow oil 600 mg (86%). LC-MS: Rt 5.3 min; *m/z* 294 [M –t-butyl+H]$^+$. ^{1}H NMR (500 MHz, DMSO-d_6) δ 8.20 – 8.16 (m, 2H), 7.64 – 7.59 (m, 2H), 3.92 (bs, 4H), 3.81 (s, 2H), 2.35 (d, *J* = 6.7 Hz, 2H), 1.68 (td, *J* = 11.1, 9.0, 3.2 Hz, 4H), 1.39 (s, 9H), 1.02 – 0.91 (m, 2H).

2-{[(4-Nitrophenyl)methyl]amino}acetonitrile

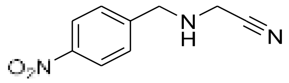

The title compound was synthesized applying the general procedure 1 method B using 4-nitrobenzylamine hydrochloride (700 mg, 3.71 mmol), chloroacetonitrile (0.23 mL, 3.71 mmol) and K_2CO_3 (1026 mg, 7.42 mmol) in acetonitrile (10 mL). Brown oil 705 mg (99%). LC-MS: Rt 2.4 min; *m/z* 192 [M+H]$^+$. ^{1}H NMR (500 MHz, DMSO-d_6) δ 8.24 – 8.15 (m, 2H), 7.66 – 7.58 (m, 2H), 3.90 (d, *J* = 6.1 Hz, 2H), 3.65 (d, *J* = 7.2 Hz, 2H), 3.29 – 3.23 (m, 1H).

[(4-Nitrophenyl)methyl](prop-2-yn-1-yl)amine

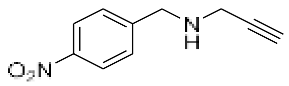

The title compound was synthesized applying the general procedure 1 method B using 4-nitrobenzyl bromide (700 mg, 3.24 mmol), propargylamine (0.21 mL, 3.24 mmol) and K_2CO_3 (896 mg, 6.48 mmol) in acetonitrile (10 mL). Brown oil 603 mg (98%). LC-MS: Rt 1.2 min; *m/z* 191 [M+H]$^+$. ^{1}H NMR (601 MHz, DMSO-d_6) δ 8.20 – 8.17 (m, 2H), 7.64 – 7.59 (m, 2H), 3.88 (s, 2H), 3.81 (s, 1H), 3.31 (d, *J* = 2.3 Hz, 2H), 3.11 (t, *J* = 2.4 Hz, 1H).

[(4-Nitrophenyl)methyl](propyl)amine

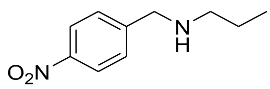

The title compound was synthesized applying the general procedure 1 method B using 4-nitrobenzyl bromide (700 mg, 3.24 mmol), 1-propylamine (0.27 mL, 3.24 mmol) and K_2CO_3 (896 mg, 6.48 mmol) in acetonitrile (10 mL). Brown oil 600 mg (95%). LC-MS: Rt 1.2 min; *m/z* 195 $[M+H]^+$. ^{1}H NMR (500 MHz, DMSO-d_6) δ 8.20 – 8.14 (m, 2H), 7.64 – 7.58 (m, 2H), 3.80 (s, 2H), 2.44 (t, *J* = 7.4 Hz, 2H), 2.23 (s, 1H), 1.43 (sxt, *J* = 7.4 Hz, 2H), 0.87 (t, *J* = 7.4 Hz, 3H).

Ethyl 2-({ethyl[(4-nitrophenyl)methyl]carbamoyl}amino)acetate

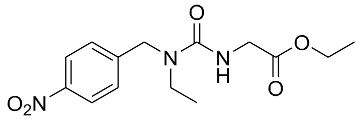

The title compound was synthesized applying the general procedure 2 using ethyl[(4-nitrophenyl)methyl]amine (480 mg, 2.66 mmol), ethyl isocyanatoacetate (0.3 mL, 2.66 mmol) in 10 mL of DCM. Yellow oil 800 mg (97%). LC-MS: Rt 5.6 min; *m/z* 332 $[M-Na]^+$. ^{1}H NMR (500 MHz, DMSO-d_6) δ 8.17-8.23 (m, 2H), 7.53 – 7.48 (m, 2H), 6.95 (t, *J* = 5.9 Hz, 1H), 4.57 (s, 2H), 4.10 (q, *J* = 6.9 Hz, 2H), 3.77 (d, *J* = 6.0 Hz, 2H), 3.24 (q, *J* = 7.0 Hz, 2H), 1.20 (t, *J* = 7.1 Hz, 3H), 1.04 (t, *J* = 7.0 Hz, 3H). ^{13}C NMR (126 MHz, DMSO-d_6) δ 171.48, 157.88, 148.24, 146.95, 128.62 (2C), 123.90 (2C), 60.60, 48.95, 42.86, 41.49, 14.59, 13.81.

Ethyl 2-({[(4-nitrophenyl)methyl](propan-2-yl)carbamoyl}amino)acetate

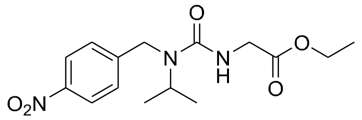

The title compound was synthesized applying the general procedure 2 using [(4-nitrophenyl)methyl](propan-2-yl)amine (600 mg, 3.09 mmol) and ethyl isocyanatoacetate (0.35 mL, 3.09 mmol) in 10 mL of DCM. Yellow oil 990 mg (99%). LC-MS: Rt 5.9 min; *m/z* 346 $[M-Na]^+$. ^{1}H NMR (500 MHz, DMSO-d_6) δ 8.20 – 8.15 (m, 2H), 7.55 – 7.48 (m, 2H), 6.91 (t, *J* = 5.8 Hz, 1H), 4.52 (s, 2H), 4.31 (h, *J* = 6.8 Hz, 1H), 4.10 (q, *J* = 7.1 Hz, 2H), 3.75 (d, *J* = 5.9 Hz, 2H), 1.20 (t, *J* = 7.1 Hz, 3H), 1.03 (d, *J* = 7.3 Hz, 6H). ^{13}C NMR (126 MHz, DMSO-d_6) δ 171.47, 158.17, 149.87, 146.62, 128.13 (2C), 123.66 (2C), 60.58, 46.68, 44.13, 42.94, 21.04 (2C), 14.58.

Ethyl 2-{[(cyclopropylmethyl)[(4-nitrophenyl)methyl]carbamoyl]amino}acetate

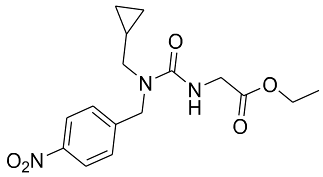

The title compound was synthesized applying the general procedure 2 using (Cyclopropylmethyl)[(4-nitrophenyl)methyl]amine (380 mg, 1.84 mmol) and ethyl isocyanatoacetate (0.2 mL, 1.84 mmol) in 10 mL of DCM. Yellow oil 500 mg (81%). LC-MS: Rt 5.8 min; *m/z* 358 $[M-Na]^+$. ^{1}H NMR (500 MHz, DMSO-d_6) δ 8.23 – 8.13 (m, 2H), 7.54 – 7.46 (m, 2H), 6.98 (t, *J* = 5.8 Hz, 1H), 4.67 (s, 2H), 4.14 – 4.07 (m, 2H), 3.76 (d, *J* = 5.9 Hz, 2H), 3.13 (d, *J* = 6.8 Hz, 2H), 1.24 – 1.14 (m, 3H), 0.95 (ddtd, *J* = 11.7, 8.0, 6.8, 4.9 Hz, 1H), 0.40 –0.31 (m, 2H), 0.21 – 0.12 (m, 2H). ^{13}C NMR (126 MHz, DMSO-d_6) δ 171.45, 158.05, 148.24, 146.86, 128.47 (2C), 123.80 (2C), 60.88, 51.05, 49.59, 42.78, 14.58, 10.66, 3.81 (2C).

Ethyl 2-{[(cyanomethyl)[(4-nitrophenyl)methyl]carbamoyl]amino}acetate

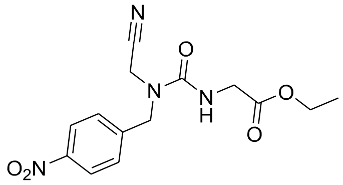

The title compound was synthesized applying the general procedure 2 using 2-{[(4-nitrophenyl)methyl]amino}acetonitrile (600 mg, 3.14 mmol) and ethyl isocyanatoacetate (0.35 mL, 3.14 mmol) in 10 mL of DCM. Brown oil 900 mg (90%). LC-MS: Rt 5.5 min; *m/z* 321 $[M+H]^+$. ^{1}H NMR (500 MHz, DMSO-d_6) δ 8.26 – 8.21 (m, 2H), 7.58 – 7.54 (m, 2H), 7.51 – 7.48 (m, 1H), 4.72 (s, 2H), 4.38 (s, 2H), 4.13 – 4.09 (m, 2H), 3.83 – 3.79 (m, 2H), 1.22 – 1.19 (m, 3H). ^{13}C NMR (126 MHz, DMSO-d_6) δ 170.95, 157.32, 147.30, 145.77, 128.84 (2C), 123.99 (2C), 67.48, 60.79, 50.49, 36.21, 25.60, 14.57.

Ethyl 2-({[(4-nitrophenyl)methyl](prop-2-yn-1-yl)carbamoyl}amino)acetate

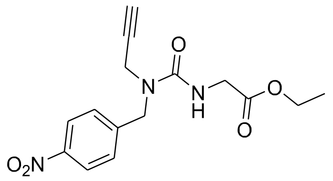

The title compound was synthesized applying the general procedure 2 using [(4-nitrophenyl)methyl](prop-2-yn-1-yl)amine (480 mg, 2.52 mmol) and ethyl isocyanatoacetate (0.28 mL, 2.5 mmol) in 10 mL of DCM. Red oil 800 mg (99%). LC-MS: Rt 5.4 min; *m/z* 320 $[M+H]^+$. ^{1}H NMR (500 MHz, DMSO-d_6) δ 8.21 (m, 2H), 7.57 – 7.51 (m, 2H), 7.20 (t, *J* = 5.8 Hz, 1H), 4.66 (s, 2H), 4.17 – 4.06 (m, 4H), 3.77 (d, *J* = 5.8 Hz, 2H), 3.18 (t, *J* = 2.4 Hz, 1H), 1.24 – 1.16 (m, 3H). ^{13}C NMR (126 MHz, DMSO-d_6) δ 171.17, 157.54, 147.09, 146.81, 128.78 (2C), 123.87 (2C), 80.35, 75.24, 60.67, 49.29, 42.90, 36.34, 14.58.

Ethyl 2-({[(4-nitrophenyl)methyl](propyl)carbamoyl}amino)acetate

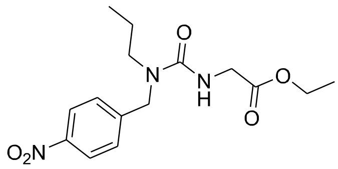

The title compound was synthesized applying the general procedure 2 using [(4-nitrophenyl)methyl](propyl)amine (600 mg, 3.09 mmol) and ethyl isocyanatoacetate (0.35 mL, 3.09 mmol) in 10 mL of DCM. Yellow oil 990 mg (99%). LC-MS: Rt 5.8 min; *m/z* 346 $[M-Na]^+$. ^1H NMR (400 MHz, DMSO-d_6) δ 8.24 – 8.16 (m, 2H), 7.52 – 7.46 (m, 2H), 6.94 (t, *J* = 5.8 Hz, 1H), 4.58 (s, 2H), 4.09 (q, *J* = 7.1 Hz, 2H), 3.76 (d, *J* = 5.8 Hz, 2H), 3.17 – 3.09 (m, 2H), 1.49 (sxt, *J* = 7.3 Hz, 2H), 1.20 (t, *J* = 7.2 Hz, 3H), 0.81 (t, *J* = 7.4 Hz, 3H). ^{13}C NMR (126 MHz, DMSO-d_6) δ 171.45, 158.10, 148.25, 146.93, 128.57 (2C), 123.87 (2C), 60.62, 49.45, 48.54, 42.89, 21.46, 14.59, 11.41.

Ethyl 2-({[(4-nitrophenyl)methyl][(1*H*-1,2,3,4-tetrazol-5-yl)methyl]carbamoyl}amino)acetate

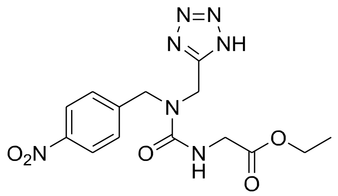

To a 100 mL round-bottomed flask equipped with a stir bar was added ethyl 2-{[(cyanomethyl)[(4-nitrophenyl)methyl]carbamoyl]-amino}acetate (700 mg, 2.19 mmol), 12 mL of DMF, sodium azide (256 mg, 3.93 mmol), and ammonium chloride (222 mg, 4.15 mmol). The reaction vessel was stirred at 90 °C overnight (18 h). The reaction was cooled to RT and diluted with 50 mL of HCl (1 M aq), then extracted with ethyl acetate (3×20 mL). The organic phase was collected, dried over magnesium sulfate and filtered, then evaporated to give the desired product. Brown oil 571 mg (72%). LC-MS: Rt 5.4 min; *m/z* 364 $[M+H]^+$. ^1H NMR (500 MHz, DMSO-d_6) δ 16.23 (s, 1H), 8.24 – 8.19 (m, 2H), 7.56 – 7.51 (m, 2H), 7.31 (t, *J* = 5.7 Hz, 1H), 4.75 (s, 2H), 4.72 (s, 2H), 4.09 (q, *J* = 7.1 Hz, 2H), 3.77 (d, *J* = 5.7 Hz, 2H), 1.18 (t, *J* = 7.1 Hz, 3H). ^{13}C NMR (126 MHz, DMSO-d_6) δ 171.11, 163.73, 162.75, 157.83, 146.94, 128.65 (2C), 124.17(2C), 61.16, 50.07, 42.50, 36.25, 15.03.

Ethyl 2-({[(1-methyl-1*H*-1,2,3-triazol-4-yl)methyl][(4-nitrophenyl)methyl]carbamoyl}amino)acetate

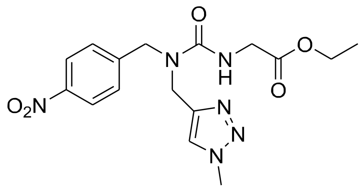

To a solution of iodomethane (0.2 mL, 433 mg, 3.05 mmol) in H_2O/DMF 1:4 (15 mL), NaN_3 (229 mg, 3.52 mmol), Na_2CO_3 (996 mg, 9.39 mmol), ascorbic acid (331 mg, 1.88 mmol), $CuSO_4*5H_2O$ (235 mg, 0.95 mmol) and ethyl 2-({[(4-nitrophenyl)methyl](prop-2-yn-1-yl)carbamoyl}amino)acetate (750 mg, 2.35

mmol) were added. The reaction mixture was stirred at RT overnight, diluted with a saturated solution of NH_4Cl (20 mL), treated with solid EDTA (1.0 g) and extracted with EtOAc (2×20 mL). The combined organic extracts were washed with H_2O (20 mL), dried over anhydrous $MgSO_4$, and concentrated under reduced pressure. The resulting residue was washed with hexane and dried under vacuum, to give the desired compound. Orange oil 860 mg (97%). LC-MS: Rt 5.4 min; *m/z* 377 $[M+H]^+$. ^1H NMR (500 MHz, DMSO-d_6) δ 8.22 – 8.17 (m, 2H), 7.91 (s, 1H), 7.52 – 7.48 (m, 2H), 7.20 (t, J = 5.8 Hz, 1H), 4.59 (s, 2H), 4.45 (s, 2H), 4.11 (q, J = 7.1 Hz, 2H), 4.01 (s, 3H), 3.79 (d, J = 5.8 Hz, 2H), 1.20 (t, J = 7.1 Hz, 3H). ^{13}C NMR (126 MHz, DMSO-d6) δ 171.34, 162.76, 157.95, 147.02, 144.22, 128.71 (2C), 123.90 (2C), 60.67, 49.18, 42.95, 41.68, 36.68, 31.24, 14.57.

Ethyl 2-({[(2-methyl-2H-1,2,3,4-tetrazol-5-yl)methyl][(4-nitrophenyl)methyl]carbamoyl}amino)acetate

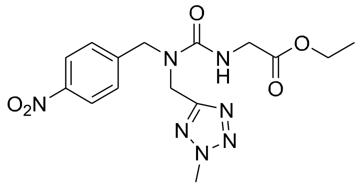

The tetrazole derivative ethyl 2-({[(4-nitrophenyl)methyl][(1*H*-1,2,3,4-tetrazol-5-yl)methyl]carbamoyl}amino)acetate (700 mg, 1.93 mmol) was dissolved in a solution of triethylamine (0.4 mL, 2.70 mmol) and acetonitrile (10 mL). The solution was heated to reflux temperature (90 °C), followed by the dropwise addition of Iodomethane (0.2 mL, 2.60 mmol). Upon completion, the solution was allowed to cool and stirred at RT for three days, then evaporated to dryness. The crude liquid containing a mixture of the 2- and 1-regioisomers was purified using silica gel chromatography (elution with DCM/MeOH) to give the desired 2-regioisomer. Brown oil 170 mg (23%). LC-MS: Rt 5.4 min; *m/z* 378 $[M+H]^+$. ^1H NMR (601 MHz, DMSO-d_6) δ 8.21 – 8.19 (m, 2H), 7.53 – 7.49 (m, 2H), 7.25 (t, J = 5.8 Hz, 1H), 4.72 (s, 2H), 4.68 (s, 2H), 4.31 (s, 3H), 4.09 (q, J = 7.1 Hz, 2H), 3.77 (d, J = 5.7 Hz, 2H), 1.19 (t, J = 7.1 Hz, 3H). ^{13}C NMR (151 MHz, DMSO-d_6) δ 171.30, 163.73, 162.76, 157.86, 147.23, 128.50 (2C), 123.91 (2C), 60.69, 49.70, 42.91, 36.25, 31.24, 14.56.

tert-Butyl 4-{[(2-ethoxy-2-oxoethyl)carbamoyl][(4-nitrophenyl)methyl]amino}piperidine-1-carboxylate

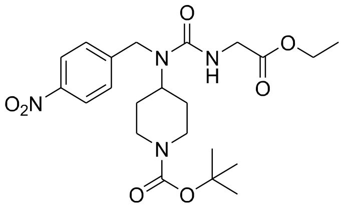

The title compound was synthesized applying the general procedure 2 using *tert*-butyl 4-{[(4-nitrophenyl)-methyl]amino}piperidine-1-carboxylate (600 mg, 1.79 mmol) and ethyl isocyanatoacetate (0.20 mL, 1.79 mmol) in 10 mL of DCM. Yellow oil 800 mg (96%). LC-MS: Rt 6.0 min; *m/z* 487 $[M-Na]^+$. ^1H NMR (601 MHz, DMSO-d_6) δ 8.19 – 8.15 (m, 2H), 7.52 (d, *J* = 8.6 Hz, 2H), 7.02 (t, *J* = 5.9 Hz, 1H), 4.57 (s, 2H), 4.20 (q, *J* = 7.1 Hz, 2H), 3.77 (d, *J* = 5.9 Hz, 2H), 3.63 – 3.59 (m, 4H), 3.54 – 3.49 (m, 1H), 1.78 – 1.75 (m, 4H), 1.36 (s, 9H), 1.23 (t, *J* = 7.1 Hz, 3H). ^{13}C NMR (126 MHz, DMSO-d_6) δ 171.42, 158.05, 154.10, 146.67, 128.11 (2C), 125.49, 123.71 (2C), 79.07, 67.48, 60.65, 60.61, 53.31, 42.95, 41.91, 35.22, 28.54 (3C), 25.59, 14.58.

tert-Butyl 4-({[(2-ethoxy-2-oxoethyl)carbamoyl][(4-nitrophenyl)methyl]amino}methyl)piperidine-1-carboxylate

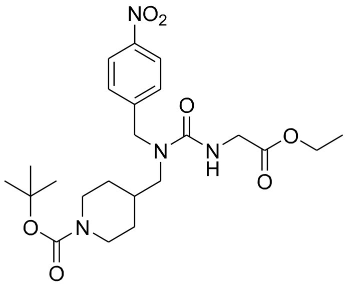

The title compound was synthesized applying the general procedure 2 using *tert*-butyl 4-({[(4-nitrophenyl)methyl]amino}methyl)-piperidine-1-carboxylate (600 mg, 1.72 mmol) and ethyl isocyanatoacetate (0.20 mL, 1.72 mmol) in 10 mL of DCM. Yellow oil 800 mg (97%). LC-MS: Rt 6.1 min; *m/z* 501 $[M-Na]^+$. ^1H NMR (601 MHz, DMSO-d_6) δ 8.22 – 8.17 (m, 2H), 7.50 – 7.44 (m, 2H), 6.94 (t, *J* = 5.9 Hz, 1H), 4.60 (s, 2H), 4.09 (d, *J* = 7.1 Hz, 2H), 3.77 (d, *J* = 5.9 Hz, 2H), 3.75 (dd, *J* = 5.9, 3.2 Hz, 2H), 3.63 – 3.58 (m, 2H), 3.09 (d, *J* = 7.3 Hz, 2H), 1.79 – 1.74 (m, 2H), 1.61 – 1.53 (m, 3H), 1.39 (s, 9H), 1.21 (t, *J* = 7.1 Hz, 3H). ^{13}C NMR (126 MHz, DMSO-d_6) δ 171.70, 158.19, 154.27, 129.33, 128.52 (2C), 124.39, 123.88 (2C), 78.90, 67.48, 66.66, 60.63, 51.77, 49.95, 42.90, 41.96, 35.22, 28.57 (3C), 25.59, 14.57.

3-{2-[2-(2-Bromophenyl)pyrrolidin-1-yl]-2-oxoethyl}-1-[(4-nitrophenyl)methyl]-1-(prop-2-yn-1-yl)urea

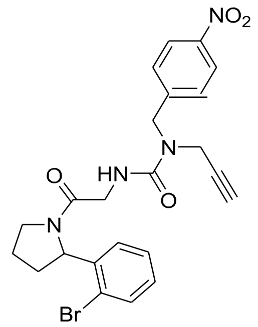

The title compound was synthesized applying the general procedure 3 using ethyl 2-({[(4-nitrophenyl)-methyl](prop-2-yn-1-yl)carbamoyl}amino)-acetate (2100 mg, 6.58 mmol) and LiOH monohydrate (1380 mg, 32.88 mmol) in 16 mL of MeOH/THF 1:1, then 4 mL of water. The so-obtained carboxylic acid (1368 mg, 4.70 mmol) was then reacted with 2-(2-Bromophenyl)-pyrrolidine (905 mg, 4.00 mmol) HATU (2130 mg, 5.60 mmol) and DIPEA (1.05 mL, 6.00 mmol) in DMF (20 mL). Brown oil 1499 mg (46% over two steps). LC-MS: Rt 6.1 min; *m/z* 499 $[M+H]^+$. ^{1}H NMR (601 MHz, DMSO-d_6) δ 8.20 – 8.15 (m, 2H), 7.55 – 7.50 (m, 2H), 7.32 – 7.23 (m, 2H), 7.20 – 7.10 (m, 2H), 6.77 (t, *J* = 5.3 Hz, 1H), 4.65 (s, 2H), 4.08 (d, *J* = 2.5 Hz, 2H), 3.97 (d, *J* = 5.4 Hz, 2H), 3.94 – 3.87 (m, 1H), 3.66 – 3.58 (m, 2H), 3.17 (t, *J* = 2.4 Hz, 1H), 2.30 – 2.23 (m, 1H), 1.98 – 1.90 (m, 1H), 1.84 – 1.66 (m, 2H). ^{13}C NMR (126 MHz, DMSO-d_6) δ 168.20, 157.50, 147.04, 142.38, 133.02, 128.99, 128.84, 128.81 (2C), 127.93, 127.26, 123.86 (2C), 121.83, 80.39, 75.27, 60.74, 49.45, 46.75, 43.49, 36.44, 32.29,23.34.

3-{2-[2-(2-Bromophenyl)pyrrolidin-1-yl]-2-oxoethyl}-1-[(1-methyl-1*H*-1,2,3-triazol-4-yl)methyl]-1-[(4-nitrophenyl)methyl]urea

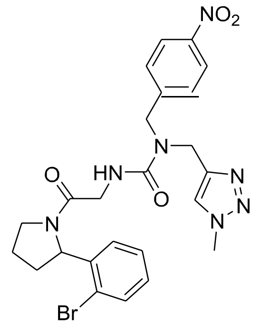

The title compound was synthesized applying the general procedure 3 using ethyl 2-({[(1-methyl-1*H*-1,2,3-triazol-4-yl)methyl][(4-nitrophenyl)methyl]carbamoyl}-amino)acetate (600 mg, 1.59 mmol) and LiOH monohydrate (334 mg, 7.97 mmol) in 8 mL of MeOH/THF 1:1, then 2 mL of water. The so-obtained carboxylic acid (254 mg, 0.73 mmol) was then reacted with 2-(2-Bromophenyl)-pyrrolidine (150 mg, 0.66 mmol), HATU (353 mg, 0.93 mmol) and DIPEA (0.17 mL, 1.00 mmol) in DMF (10 mL). Brown oil 311 mg (35% over two steps). LC-MS: Rt 5.9 min, *m/z* 556 $[M+H]^+$. ^{1}H NMR (500 MHz, DMSO-d_6) δ 8.18 – 8.14 (m, 2H), 7.91 (s, 1H), 7.51 – 7.46 (m, 2H), 7.31 – 7.23 (m, 2H), 7.21 – 7.12 (m, 2H), 6.81 (t, *J* = 5.4 Hz, 1H), 4.58 (s, 2H), 4.43 (s, 2H), 3.99 (d, *J* = 5.4 Hz, 2H), 3.96 (s, 3H), 3.93 – 3.86 (m, 1H), 3.61 (qd, *J* = 11.1, 10.3, 5.4 Hz, 2H), 232 – 2.27 (m, 2H), 1.98 – 1.66 (m, 2H). ^{13}C NMR (126 MHz, DMSO-d_6) δ 169.44, 156.94, 147.65, 138.41, 137.91, 136.57, 132.88, 128.83, 128.04 (2C), 127.62, 125.34, 124.66, 124.42 (2C), 121.84, 64.96, 52.05, 46.78, 43.43, 42.85, 37.72, 32.46, 24.61.

3-{2-[2-(2-Bromophenyl)pyrrolidin-1-yl]-2-oxoethyl}-1-[(2-methyl-2H-1,2,3,4-tetrazol-5-yl)methyl]-1-[(4-nitrophenyl)methyl]urea

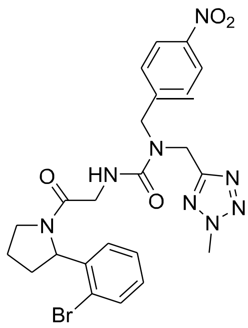

The title compound was synthesized applying the general procedure 3, using ethyl 2-({[(2-methyl-2*H*-1,2,3,4-tetrazol-5-yl)methyl][(4-nitrophenyl)methyl]-carbamoyl}amino)acetate (602 mg, 1.60 mmol), and LiOH monohydrate (335 mg, 7.98 mmol) in 8 mL of MeOH/THF 1:1, then 2 mL of water. The so-obtained carboxylic acid (255 mg, 0.73 mmol) was then reacted with 2-(2-Bromophenyl)-pyrrolidine (150 mg, 0.66 mmol) HATU (353 mg, 0.93 mmol) and DIPEA (0.17 mL, 1.00 mmol) in DMF (10 mL). Brown oil 221 mg (25% over two steps). LC-MS: Rt 5.9 min; *m/z* 558 $[M+H]^+$. ^1H NMR (500 MHz, DMSO-d_6) δ 8.29 – 8.22 (m, 2H), 7.63 – 7.53 (m, 2H), 7.48 – 7.42 (m, 2H), 7.08 – 7.03 (m, 2H), 6.27 (s, 1H), 4.65 (s, 2H), 4.54 (s, 2H), 4.09 (s, 3H), 3.97 (d, J = 5.2 Hz, 2H), 3.93 – 3.86 (m, 1H), 3.61 – 3.58 (m, 2H), 2.30 – 2.23 (m, 1H), 1.98-1.90 (m, 1H), 1.84 – 1.66 (m, 2H). ^{13}C NMR (126 MHz, DMSO-d_6) δ 168.82, 157.22, 147.35, 140.39, 135.46, 132.78, 130.86, 128.82 (2C), 127.98, 127.44, 125.00, 124.14 (2C), 121.84, 70.11, 56.39, 48.11, 45.09, 43.17, 37.08, 32.37, 23.97.

Synthesis of final products

Ethyl 2-({[(4-aminophenyl)methyl](ethyl)carbamoyl}amino)acetate hydrochloride (2).

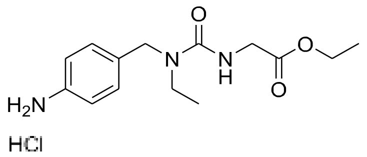

The title compound was synthesized according to the general procedure 4, starting from Fe powder (235 mg, 4.21 mmol), calcium chloride (156 mg, 1.40 mmol), ethyl 2-({ethyl[(4-nitrophenyl)methyl]carbamoyl}-amino)acetate (434 mg, 1.40 mmol), in Ethanol/Water (12 mL, 10:2). The resultant oil was dissolved in a small amount of ethanol, to which HCl (1.25 M in ethanol) was added. Evaporation of the solvent produced the title compound as yellow solid 145 mg (33%). Mp: 88−91 °C. LC−MS: Rt = 1.3 min, *m/z* 302 $[M + Na]^+$. ^1H NMR (500 MHz, DMSO-d_6) δ 10.22 (bs, 2H), 7.38 – 7.31 (m, 4H), 6.91 (s, 1H), 4.45 (s, 2H), 4.09 (q, J = 7.2 Hz, 2H), 3.76 (s, 2H), 3.19 (q, J = 7.0 Hz, 2H), 1.20 (t, J = 7.0 Hz, 3H), 1.01 (t, J = 6.8 Hz, 3H). ^{13}C NMR (126 MHz, DMSO-d_6) δ 171.62, 157.82, 139.80, 130.95, 128.81 (2C), 123.50 (2C), 60.57, 48.61,

42.86, 41.06, 14.60, 13.75. ESI+ (*m/z*): [M + Na] $^{+}$ calculated for $C_{14}H_{21}N_3O_3Na$ 302.1475; found 302.1460 [M + Na] $^{+}$.

Ethyl 2-({[(4-aminophenyl)methyl](propan-2-yl)carbamoyl}amino)acetate hydrochloride (3).

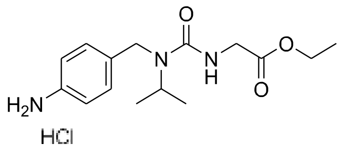

The title compound was synthesized according to the general procedure 4, starting from Fe powder (235 mg, 4.21 mmol), calcium chloride (156 mg, 1.40 mmol), ethyl 2-({[(4-nitrophenyl)methyl](propan-2-yl)carbamoyl}-amino)acetate (452 mg, 1.40 mmol), in Ethanol/Water (12 mL, 10:2). The resultant oil was dissolved in a small amount of ethanol, to which HCl (1.25 M in ethanol) was added. Evaporation of the solvent produced the title compound as yellow solid 200 mg (43%). Mp: 71−75 °C. LC−MS: Rt = 1.4 min, *m/z* 316 [M + Na]$^{+}$. ^{1}H NMR (500 MHz, DMSO-*d6*) δ 10.09 (bs, 2H), 7.39 – 7.36 (m, 2H), 7.33 – 7.27 (m, 2H), 6.82 (s, 1H), 4.41 (s, 2H), 4.28 (h, *J* = 6.9 Hz, 1H), 4.12 – 4.05 (m, 2H), 3.74 (d, *J* = 5.8 Hz, 2H), 1.20 (t, *J* = 6.7 Hz, 3H), 1.05 – 0.99 (m, 6H). ^{13}C NMR (126 MHz, DMSO-d_6) δ 171.70, 158.15, 130.60, 129.13, 128.27 (2C), 123.22 (2C), 60.97, 41.88, 41.43, 31.16, 23.63, 21.11, 14.80. ESI+ (*m/z*): [M + Na] $^{+}$ calculated for $C_{15}H_{23}N_3O_3Na$ 316.1631; found 316.1640 [M + Na] $^{+}$.

Ethyl 2-({[(4-aminophenyl)methyl](propyl)carbamoyl}amino)acetate (4).

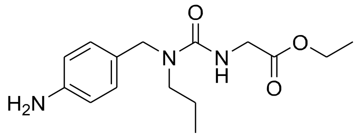

The title compound was synthesized according to the general procedure 4, starting from Fe powder (503 mg, 9.00 mmol), calcium chloride (333 mg, 3.00 mmol), ethyl 2-({[(4-nitrophenyl)methyl](propyl)carbamoyl}-amino)acetate (970 mg, 3.00 mmol), in Ethanol/Water (24 mL, 20:4). Yellow oil 550 mg (62%). LC−MS: Rt = 4.7 min, *m/z* 316 [M + Na]$^{+}$. ^{1}H NMR (500 MHz, DMSO-d_6) δ 6.92 – 6.89 (m, 2H), 6.72 (t, *J* = 5.6 Hz, 1H), 6.52 – 6.49 (m, 2H), 4.24 (s, 2H), 4.09 (q, *J* = 7.1 Hz, 2H), 3.76 (d, *J* = 5.8 Hz, 2H), 3.03 – 2.97 (m, 2H), 1.49 (h, *J* = 7.3 Hz, 2H), 1.20 (t, *J* = 7.2 Hz, 3H), 0.81 (t, *J* = 7.4 Hz, 3H). ^{13}C NMR (126 MHz, DMSO-d_6) δ 171.66, 157.97, 147.90, 128.65 (2C), 126.10, 114.38 (2C), 60.77, 49.02, 47.26, 42.76, 21.13, 14.80, 11.54. ESI+ (*m/z*): [M + Na] $^{+}$ calculated for $C_{15}H_{23}N_3O_3Na$ 316.1631; found 316.1614 [M + Na] $^{+}$.

Ethyl 2-({[(4-aminophenyl)methyl](prop-2-yn-1-yl)carbamoyl}amino)acetate (5).

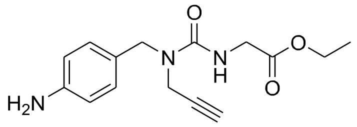

The title compound was synthesized according to the general procedure 4, starting from Fe powder (487 mg, 8.73 mmol), calcium chloride (323 mg, 2.91 mmol), ethyl 2-({[(4-nitrophenyl)methyl](prop-2-yn-1-yl)carbamoyl}amino)acetate (929 mg, 2.91 mmol), in Ethanol/Water (24 mL, 20:4). Yellow oil 350 mg (42%). LC−MS: Rt = 4.3 min, *m/z* 312 $[M + Na]^{+}$. ^{1}H NMR (500 MHz, DMSO-d_6) δ 7.40 – 7.37 (m, 2H), 7.36 – 7.33 (m, 2H), 7.19 (t, *J* = 6.0 Hz, 1H), 4.54 (s, 2H), 4.10 (q, *J* = 7.1 Hz, 1H), 4.02 (d, *J* = 2.5 Hz, 2H), 3.77 (d, *J* = 5.9 Hz, 2H), 3.18 (t, *J* = 2.4 Hz, 1H), 1.20 (t, *J* = 7.1 Hz, 3H). ^{13}C NMR (126 MHz, DMSO-d_6) δ 171.26, 157.55, 138.24, 131.25, 128.90 (2C), 123.59 (2C), 80.43, 75.02, 60.65, 48.91, 42.90, 35.79, 14.60. ESI+ (*m/z*): $[M + Na]^{+}$ calculated for $C_{15}H_{19}N_3O_3Na$ 312.1318; found 312.1316 $[M + Na]^{+}$.

Ethyl 2-({[(4-aminophenyl)methyl](cyanomethyl)carbamoyl}amino)acetate hydrochloride (6).

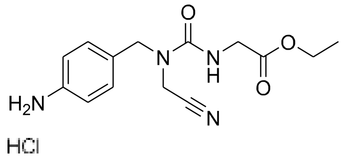

The title compound was synthesized according to the general procedure 4, starting from Fe powder (124 mg, 2.22 mmol), calcium chloride (82 mg, 0.74 mmol), ethyl 2-{[(cyanomethyl)[(4-nitrophenyl)methyl]carbamoyl]-amino}acetate (237 mg, 0.74 mmol), in Ethanol/Water (8 mL, 6:2). The resultant oil was dissolved in a small amount of ethanol, to which HCl (1.25 M in ethanol) was added. Evaporation of the solvent produced the title compound as an orange solid 600 mg (25%). Mp: 91−95 °C. LC−MS: Rt = 1.3 min, *m/z* 314 $[M + Na]^{+}$. ^{1}H NMR (500 MHz, DMSO-d_6) δ 9.73 (bs, 2H), 7.32 (d, *J* = 8.3 Hz, 2H), 7.25 (d, *J* = 8.0 Hz, 2H), 6.97 (t, *J* = 7.9 Hz, 1H), 4.52 (s, 2H), 4.22 (s, 2H), 4.17 – 4.13 (m, 2H), 4.03 (d, *J* = 7.9 Hz, 2H), 1.23 – 1.20 (m, 3H). ^{13}C NMR (126 MHz, DMSO-d_6) δ 172.19, 170.08, 167.90, 156.09, 129.28 (4C), 122.40, 61.78, 43.56, 33.40, 25.86, 14.51. ESI+ (*m/z*): $[M + Na]^{+}$ calculated for $C_{14}H_{19}N_4O_3Na$ 314.1349; found 314.1360 $[M + Na]^{+}$.

Ethyl 2-({[(4-aminophenyl)methyl](cyclopropylmethyl)carbamoyl}amino)acetate hydrochloride (7).

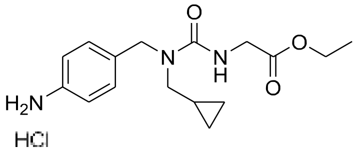

The title compound was synthesized according to the general procedure 4, starting from Fe powder (75 mg, 1.34 mmol), calcium chloride (50 mg, 0.45 mmol), ethyl 2-{[(cyclopropylmethyl)[(4-nitrophenyl)methyl]carbamoyl]-

amino}acetate (150 mg, 0.45 mmol), in Ethanol/Water (8 mL, 6:2). The resultant oil was dissolved in a small amount of ethanol, to which HCl (1.25 M in ethanol) was added. Evaporation of the solvent produced the title compound as an orange solid 150 mg (98%). Mp: 101−105 °C. LC−MS: Rt = 4.8 min, *m/z* 328 [M + Na]$^+$. ^{1}H NMR (500 MHz, DMSO-d_6) δ 10.32 (bs, 2H), 7.37 – 7.31 (m, 4H), 6.94 (s, 1H), 4.56 (s, 2H), 4.09 (q, *J* = 7.1 Hz, 2H), 3.75 (s, 2H), 3.07 (d, *J* = 6.8 Hz, 2H), 2.88 (dd, *J* = 6.7, 1.4 Hz, 1H), 1.20 (t, *J* = 6.7 Hz, 3H), 0. 39 – 0.35 (m, 2H), 0.18 – 0.10 (m, 2H). ^{13}C NMR (126 MHz, DMSO-d_6) δ 171.52, 158.08, 139.79, 128.76, 128.70 (2C), 123.54 (2C), 60.58, 50.51, 49.12, 42.91, 14.60, 10.55, 3.75, 3.44. ESI+ (*m/z*): [M + Na]$^+$ calculated for $C_{16}H_{23}N_3O_3Na$ 328.1631; found 328.1650 [M + Na]$^+$.

Ethyl 2-({[(4-aminophenyl)methyl][(1-methyl-1*H*-1,2,3-triazol-4-yl)methyl]carbamoyl}amino)acetate hydrochloride (8).

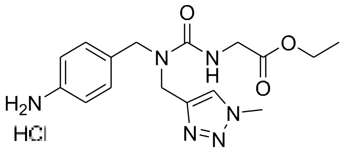

The title compound was synthesized according to the general procedure 4, starting from Fe powder (85 mg, 1.51 mmol), calcium chloride (56 mg, 0.50 mmol), ethyl 2-({[(1-methyl-1*H*-1,2,3-triazol-4-yl)methyl][(4-nitrophenyl)-methyl]carbamoyl}amino)acetate (190 mg, 0.50 mmol), in Ethanol/Water (8 mL, 6:2). The resultant oil was dissolved in a small amount of ethanol, to which HCl (1.25 M in ethanol) was added. Evaporation of the solvent produced the title compound as an orange solid 155 mg (80%). Mp: 144−147 °C. LC−MS: Rt = 1.2 min, *m/z* 369 [M + Na]$^+$. ^{1}H NMR (500 MHz, DMSO-d_6) δ 10.29 (bs, 2H), 7.87 (s, 1H), 7.41 – 7.30 (m, 4H), 7.18 (s, 1H), 4.47 (s, 2H), 4.39 (s, 2H), 4.13 – 4.07 (m, 2H), 4.01 (s, 3H), 3.78 (s, 2H), 1.23 – 1.17 (m, 3H). ^{13}C NMR (126 MHz, DMSO-d_6) δ 171.41, 157.94, 144.36, 138.82, 130.99, 129.01 (2C), 124.60, 123.62 (2C), 60.64, 56.48, 48.74, 42.94, 36.70, 14.60. ESI+ (*m/z*): [M + Na]$^+$ calculated for $C_{16}H_{22}N_6O_3Na$ 369.1645; found 369.1650 [M + Na]$^+$.

Ethyl 2-({[(4-aminophenyl)methyl][(2-methyl-2H-1,2,3,4-tetrazol-5yl)methyl]carbamoyl}amino)acetate hydrochloride (9).

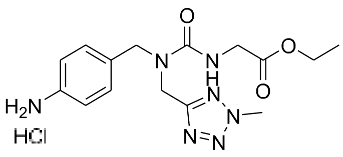

The title compound was synthesized according to the general procedure 4, starting from Fe powder (67 mg, 1.19 mmol), calcium chloride (44 mg, 0.40

mmol), ethyl 2-({[(2-methyl-2H-1,2,3,4-tetrazol-5-yl)methyl][(4-nitrophenyl)methyl]carbamoyl}amino)acetate (150 mg, 0.40 mmol), in Ethanol/Water (8 mL, 6:2). The resultant oil was dissolved in a small amount of ethanol, to which HCl (1.25 M in ethanol) was added. Evaporation of the solvent produced the title compound as an orange solid 50 mg (33%). Mp: 128−131 °C. LC−MS: Rt = 2.9 min, *m/z* 370 $[M + Na]^+$. ^{1}H NMR (400 MHz, DMSO-d_6) δ 9.87 (bs, 2H), 7.37 – 7.31 (m, 2H), 7.31 – 7.25 (m, 2H), 7.23 (t, *J* = 5.5 Hz, 1H), 4.64 (s, 2H), 4.53 (s, 2H), 4.32 (s, 3H), 4.09 (q, *J* = 7.1 Hz, 2H), 3.77 (d, *J* = 5.5 Hz, 2H), 1.20 (t, *J* = 7.1 Hz, 3H). ^{13}C NMR (126 MHz, DMSO-d_6) δ 169.32, 158.73, 156.94, 144.54, 128.55 (2C), 126.17 (2C), 117.15, 61.61, 52.05, 43.44, 41.86, 32.67, 14.60. ESI+ (*m/z*): $[M + Na]^+$ calculated for $C_{15}H_{21}N_7O_3Na$ 370.1598; found 370.1600 $[M + Na]^+$.

Ethyl 2-({[(4-aminophenyl)methyl][(1*H*-1,2,3,4-tetrazol-5-yl)methyl]carbamoyl}amino)acetate hydrochloride (10).

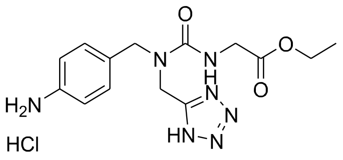

The title compound was synthesized according to the general procedure 4, starting from Fe powder (263 mg, 4.71 mmol), calcium chloride (174 mg, 1.57 mmol), ethyl 2-({[(4-nitrophenyl)methyl][(1*H*-1,2,3,4-tetrazol-5-yl)methyl]-carbamoyl}amino)acetate (570 mg, 1.57 mmol), in Ethanol/Water (12 mL, 10:2). The resultant oil was dissolved in a small amount of ethanol, to which HCl (1.25 M in ethanol) was added. Evaporation of the solvent produced the title compound as an orange solid 145 mg (25%). Mp: 146−149 °C. LC−MS: Rt = 1.4 min, *m/z* 334 $[M + H]^+$. ^{1}H NMR (500 MHz, DMSO-d_6) δ 10.05 (bs, 2H), 7.37 – 7.26 (m, 5H), 4.66 (s, 2H), 4.56 (s, 2H), 4.07 (q, *J* = 6.7 Hz, 2H), 3.76 (d, *J* = 5.2 Hz, 2H), 1.05 (t, *J* = 6.8 Hz, 3H). ^{13}C NMR (126 MHz, DMSO-d_6) δ 171.18, 157.88, 147.54, 128.93 (4C), 122.94 (2C), 60.70, 56.49, 49.70, 42.91, 14.61. ESI+ (*m/z*): $[M + H]^+$ calculated for $C_{14}H_{19}N_7O_3$ 333.1543; found 333.1550 $[M + H]^+$.

Ethyl 2-({[(4-aminophenyl)methyl](piperidin-4-yl)carbamoyl}amino)acetate dihydrochloride (11)

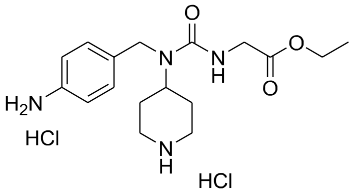

The title compound was synthesized according to the general procedure 4, starting from Fe powder (168 mg, 3.00 mmol), calcium chloride (111 mg, 1.00 mmol), *tert*-butyl 4-{[(2-ethoxy-2-oxoethyl)carbamoyl][(4-nitrophenyl)-methyl]amino}piperidine-1-carboxylate (465 mg, 1.00 mmol), in Ethanol/Water (12 mL, 10:2). The resultant oil was dissolved in a small amount of ethanol, to which HCl (1.25 M in ethanol) was added. Evaporation of the solvent produced the title compound as an orange solid 70 mg (17%). Mp: 106-110°C. LC−MS: Rt = 1.3 min, *m/z* 335 $[M + H]^+$. ^{1}H NMR (500 MHz, DMSO-d_6) δ 10.39 (bs, 2H), 8.96 (bs, 1H), 7.42 – 7.26 (m, 5H), 4.11 (q, *J* = 7.1 Hz, 2H), 3.76 (s, 2H), 3.74 (s, 2H), 3.67 – 3.62 (m, 1H), 3.26 – 3.13 (m, 2H), 2.93 (q, *J* = 11.2 Hz, 2H), 1.89 (dt, *J* = 12.9, 4.0 Hz, 2H), 1.56 (dddt, *J* = 13.9, 10.6, 7.4, 3.7 Hz, 2H), 1.19 (t, *J* = 7.1 Hz, 3H). ^{13}C NMR (126 MHz, DMSO-d_6) δ 172.85, 171.53, 157.68, 129.08 (2C), 123.76, 123.51 (2C), 60.61, 44.36, 42.55, 42.25, 41.91 (2C), 29.23 (2C), 14.59. ESI+ (*m/z*): $[M + H]^+$ calculated for $C_{17}H_{26}N_4O_3$ 334.1999; found 334.1990 $[M + H]^+$.

Ethyl 2-({[(4-aminophenyl)methyl][(piperidin-4-yl)methyl]carbamoyl}amino)acetate dihydrochloride

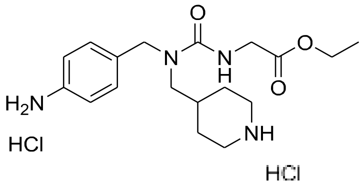

The title compound was synthesized according to the general procedure 4, starting from Fe powder (166 mg, 2.98 mmol), calcium chloride (110 mg, 0.99 mmol), *tert*-butyl 4-({[(2-ethoxy-2-oxoethyl)carbamoyl][(4-nitrophenyl)methyl]amino}methyl)piperidine-1-carboxylate (475 mg, 0.99 mmol), in Ethanol/Water (12 mL, 10:2). The resultant oil was dissolved in a small amount of ethanol, to which HCl (1.25 M in ethanol) was added. Evaporation of the solvent produced the title compound as an orange solid 145 mg (42%). Mp: 130-134°C. LC−MS: Rt = 1.4 min, *m/z* 349 $[M + H]^+$. ^{1}H NMR (500 MHz, DMSO-d_6) δ 9.08 – 8.99 (m, 2H), 8.69 (bs, 1H), 7.43 – 7.34 (m, 1H), 7.37 – 7.28 (m, 4H), 4.14 (s, 2H), 4.08 (q, *J* = 7.1 Hz, 2H), 3.75 (s, 2H), 3.24 (dd, *J* = 14.6, 9.8 Hz, 2H), 2.91 (d, *J* = 6.8 Hz, 2H), 2.85 – 2.74 (m, 2H), 1.73 (t, *J* = 14.3 Hz, 2H), 1.61 (ddt, *J* = 10.8, 7.2, 3.7 Hz, 1H), 1.42 – 1.24 (m, 2H), 1.23 (t, *J* = 7.1 Hz, 3H). ^{13}C NMR (126 MHz, DMSO-d_6) δ 171.99, 171.62, 158.54, 129.29, 128.67 (2C), 123.46 (2C), 60.59, 46.53, 44.56, 43.15, 42.96, 41.98 (2C), 26.55 (2C), 14.58. ESI+ (*m/z*): $[M + H]^+$ calculated for $C_{18}H_{28}N_4O_3$ 348.2155; found 348.2170 $[M + H]^+$.

1-[(4-Aminophenyl)methyl]-3-{2-[2-(2-bromophenyl)pyrrolidin-1-yl]-2-oxoethyl}-1-(prop-2-yn-1-yl)urea hydrochloride (13).

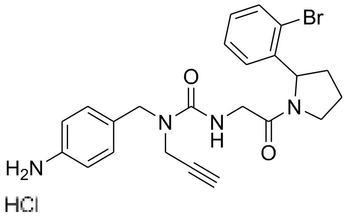

The title compound was synthesized according to the general procedure 4, starting from Fe powder (502 mg, 8.99 mmol), calcium chloride (333 mg, 3.00 mmol), 3-{2-[2-(2-bromophenyl)pyrrolidin-1-yl]-2-oxoethyl}-1-[(4-nitrophenyl)methyl]-1-(prop-2-yn-1-yl)urea (1496 mg, 3.00 mmol), in Ethanol/Water (24 mL, 20:4). The resultant oil was dissolved in a small amount of ethanol, to which HCl (1.25 M in ethanol) was added. Evaporation of the solvent produced the title compound as an orange solid 600 mg (40%). Mp: 136−140 °C. LC−MS: Rt = 5.3 min, *m/z* 469 $[M + H]^+$. ^{1}H NMR (500 MHz, DMSO-d_6) δ 10.28 (s, 2H), 7.60 (ddd, *J* = 7.9, 2.6, 1.2 Hz, 1H), 7.37 (dd, *J* = 8.6, 2.1 Hz, 2H), 7.35 – 7.24 (m, 3H), 7.16 (dddd, *J* = 12.6, 9.4, 7.6, 1.7 Hz, 2H), 6.71 (s, 1H), 5.22 – 5.16 (m, 1H), 4.53 (s, 2H), 4.02 (d, *J* = 2.4 Hz, 2H), 4.00 – 3.94 (m, 2H), 3.90 (tdd, *J* = 7.7, 4.6, 2.3 Hz, 1H), 3.60 (dtd, *J* = 16.7, 9.6, 7.7 Hz, 1H), 3.16 (dt, *J* = 7.3, 2.4 Hz, 1H), 2.26 (tdd, *J* = 11.8, 9.7, 6.8 Hz, 1H), 1.95 (tq, *J* = 10.4, 3.6 Hz, 1H), 1.80 – 1.73 (m, 1H), 1.68 (ddt, *J* = 13.6, 10.2, 6.2 Hz, 1H). ^{13}C NMR (126 MHz, DMSO-d_6) δ 168.25, 157.49, 142.39, 138.47, 133.47, 133.02, 131.11, 129.81, 129.02 (2C), 127.95, 127.27, 123.60 (2C), 80.47, 75.02, 60.72, 56.48, 49.00, 43.47, 35.97, 32.28, 24.61. ESI+ (m/z): $[M + H]^+$ calculated for $C_{23}H_{25}{}^{79}BrN_4O_2$ 468.1161; found 468.1193 $[M + H]^+$ calculated for $C_{23}H_{25}{}^{81}BrN_4O_2$ (97.3%) 470.1140; found 470.1147 $[M + H]^+$.

1-[(4-Aminophenyl)methyl]-3-{2-[2-(2-bromophenyl)pyrrolidin-1-yl]-2-oxoethyl}-1-[(1-methyl-1*H*-1,2,3-triazol-4-yl)methyl]urea hydrochloride (14).

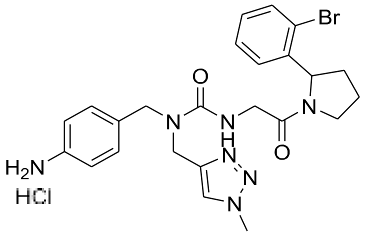

The title compound was synthesized according to the general procedure 4, starting from Fe powder (92 mg, 1.64 mmol), calcium chloride (61 mg, 0.55 mmol), 3-{2-[2-(2-bromophenyl)pyrrolidin-1-yl]-2-oxoethyl}-1-[(1-methyl-1*H*-1,2,3-triazol-4-yl)methyl]-1-[(4-nitrophenyl)methyl]urea (305 mg, 0.55 mmol), in Ethanol/Water (8 mL, 6:2). The resultant oil was dissolved in a small amount of ethanol, to which HCl (1.25 M in ethanol) was added. Evaporation of the solvent produced the title compound as an orange solid 300 mg (97%). Mp: 201−205 °C. LC−MS: Rt = 5.3 min, *m/z* 527 $[M + H]^+$. ^{1}H NMR (500 MHz,

DMSO-d_6) δ 10.33 (bs, 2H), 7.89 (s, 1H), 7.62 – 7.59 (m, 1H), 7.36 – 7.32 (m, 3H), 7.32 – 7.23 (m, 2H), 7.21 – 7.13 (m, 3H), 5.21 (dd, *J* = 8.1, 2.4 Hz, 1H), 4.46 (s, 2H), 4.38 (s, 2H), 4.01 – 3.95 (m, 5H), 3.92 – 3.88 (m, 1H), 3.61 (qd, *J* = 10.0, 7.1 Hz, 1H), 2.26 (tt, *J* = 12.1, 8.2 Hz, 1H), 1.95 (dtt, *J* = 11.0, 7.9, 4.0 Hz, 1H), 1.90 – 1.73 (m, 1H), 1.70 (ddd, *J* = 12.6, 6.2, 3.0 Hz, 1H). ^{13}C NMR (126 MHz, DMSO-d_6) δ 168.46, 157.93, 144.41, 142.40, 139.01, 133.47, 133.02, 130.86, 129.34 (2C), 127.95, 127.39, 124.66, 123.67 (2C), 121.84, 60.73, 56.48, 46.76, 43.41, 38.72, 36.80, 32.29, 23.34. ESI+ (*m/z*): [M + H] $^+$ calculated for $C_{24}H_{28}{}^{79}BrN_7O_2$ (100.0%) 525.1488; found 525.1473 [M + H] $^+$; calculated for $C_{24}H_{28}{}^{81}BrN_7O_2$ (97.3%) 527.1467; found 527.1478 [M + H] $^+$.

1-[(4-Aminophenyl)methyl]-3-{2-[2-(2-bromophenyl)pyrrolidin-1-yl]-2-oxoethyl}-1-[(2-methyl-2H-1,2,3,4-tetrazol-5-yl)methyl]urea hydrochloride (15).

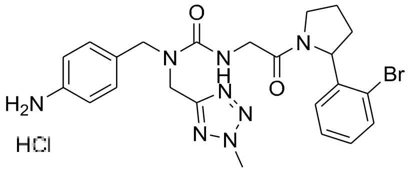

The title compound was synthesized according to the general procedure 4, starting from Fe powder (64 mg, 1.15 mmol), calcium chloride (43 mg, 0.39 mmol), 3-{2-[2-(2-bromophenyl)pyrrolidin-1-yl]-2-oxoethyl}-1-[(2-methyl-2H-1,2,3,4-tetrazol-5-yl)methyl]-1-[(4-nitrophenyl)methyl]urea (215 mg, 0.39 mmol), in Ethanol/Water (8 mL, 6:2). The resultant oil was dissolved in a small amount of ethanol, to which HCl (1.25 M in ethanol) was added. Evaporation of the solvent produced the title compound as an orange solid 120 mg (55%). Mp: 212−214 °C. LC−MS: Rt = 1.0 min, *m/z* 549 [M + Na]$^+$. ^{1}H NMR (500 MHz, DMSO-d_6) δ 10.27 (bs, 2H), 7.62 – 7.58 (m, 1H), 7.36 – 7.24 (m, 5H), 7.16 – 7.10 (m, 3H), 5.19 (dt, *J* = 8.3, 2.5 Hz, 1H), 4.46 (s, 2H), 4.38 (s, 2H), 4.30 (s, 3H), 4.01 – 3.95 (m, 2H), 3.85 (td, *J* = 11.6, 10.4, 7.1 Hz, 1H), 3.59 (dtd, *J* = 12.6, 6.9, 2.8 Hz, 1H), 2.26 (dtt, *J* = 10.4, 8.0, 5.1 Hz, 1H), 1.94 (ddt, *J* = 11.7, 7.0, 3.6 Hz, 1H), 1.79 (dtd, *J* = 8.2, 5.4, 4.7, 2.6 Hz, 1H), 1.69 (ddt, *J* = 11.8, 5.7, 2.6 Hz, 1H). ^{13}C NMR (126 MHz, DMSO-d_6) δ 168.26, 162.76, 142.32, 133.03, 129.80, 129.03 (2C), 129.00, 128.59, 128.56, 127.97, 127.24, 123.45 (2C), 121.81, 60.71, 52.07, 46.67, 43.08, 36.25, 32.33, 31.25, 23.37. ESI+ (*m/z*): [M + H] $^+$ calculated for $C_{23}H_{27}{}^{79}BrN_8O_2$ (100.0%) 526.1440; found 526.1470 [M + H] $^+$; calculated for $C_{23}H_{27}{}^{81}BrN_8O_2$ (97.3%) 528.1420; found 528.1428 [M + H] $^+$.

Ethyl 2-[4-({[(4-aminophenyl)methyl]({2-[2-(2-bromophenyl)pyrrolidin-1-yl]-2 oxoethyl}carbamoyl)amino}methyl)-1*H*-1,2,3-triazol-1-yl]acetate hydrochloride (16)

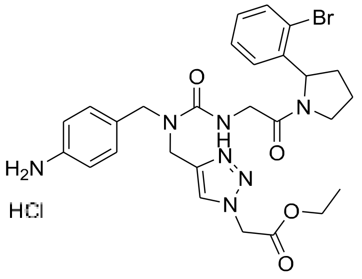

A mixture of 1-[(4-aminophenyl)methyl]-3-{2-[2-(2-bromophenyl)-pyrrolidin-1-yl]-2-oxoethyl}-1-(prop-2-yn-1-yl)urea hydrochloride (**13**) (250 mg, 0.5 mmol, 1 equiv), ethyl azidoacetate (25% solution in Ethanol, 0.4 mL, 0.6 mmol, 1.2 equiv), $CuSO_4$ (123 mg, 0.5 mmol, 1 equiv) and sodium ascorbate (196 mg, 1.0 mmol, 2 equiv) in EtOH/H_2O (20 mL, 1:1) was stirred at RT for 16 h. The reaction mixture was quenched with crushed ice and extracted with ethyl acetate (10 mL x 3). The organic extracts were washed with brine solution (20 mL) and dried over anhydrous magnesium sulfate. The solvent was evaporated under reduced pressure to afford the desired compound. The resultant brown oil was dissolved in a small amount of ethanol, to which HCl (1.25 M in ethanol) was added. Evaporation of the solvent produced the title compound as an orange solid 30 mg (10%). Mp: 186−190 °C. LC−MS: Rt = 5.3 min, *m/z* 598 $[M + H]^+$. ^{1}H NMR (500 MHz, DMSO-d_6) δ 7.97 (t, *J* = 9.6 Hz, 1H), 7.67 (dt, *J* = 8.0, 1.5 Hz, 1H), 7.36 – 7.22 (m, 4H), 7.22 – 7.11 (m, 4H), 5.30 – 5.16 (m, 1H), 4.58 – 4.48 (m, 3H), 4.39 (d, *J* = 11.2 Hz, 2H), 4.11 – 4.01 (m, 3H), 3.98 (d, *J* = 3.0 Hz, 2H), 3.67 – 3.54 (m, 2H), 1.95 (ddt, *J* = 12.5, 6.4, 3.1 Hz, 1H), 1.90 – 1.65 (m, 3H), 1.15 (t, *J* = 7.1 Hz, 3H). ^{13}C NMR (126 MHz, DMSO-d_6) δ 205.02, 163.93, 141.94, 140.54, 138.41, 138.20, 133.03, 132.88, 129.02 (2C), 128.83, 127.97, 127.62, 127.28, 123.57 (2C), 121.84, 60.76, 60.29, 48.95, 47.72, 46.76, 43.52, 42.80, 32.29, 23.36, 14.45. ESI+ (*m/z*): $[M + H]^+$ calculated for $C_{27}H_{32}{}^{79}BrN_7O_4$ (100.0%) 597.1699; found 597.1670 $[M + H]^+$; calculated for $C_{27}H_{32}{}^{81}BrN_7O_4$(97.3%) 599.1679; found 599.1668 $[M + H]^+$.

Computational Methods

System preparation

The structure of the CypA-**1** complex (PDBID: 3RDD[1]) was used as starting point to manually build different protein-ligand structures using Maestro and standard MD protein preparation procedures, including removal of co-solvent molecules and addition of missing hydrogen atoms, were followed. Input files for all

computational simulations were set up using FESetup1.2.1 software[2]. All ligands were parameterized using the GAFF[3] force field as implemented in Amber14 and AM1-BCC charges[4] while ff14SB parameters were assigned to the protein[5]. Systems were solubilized in a rectangular box with a length of 10 Å away from the edge of the solute, including TIP3P[6] water molecules and Na+ or Cl− ions to neutralize the net charge of the system. The systems were energy minimized for 300 steepest descent followed by 700 steps of conjugate gradient steps, followed by a heating step to 300 K for another 1000 steps, and finally equilibrated for 1000 steps using a NVT ensemble followed by 5000 steps of NPT ensemble at 1 atm. Harmonic potential restraints on all non-solvent atoms using a 10 kcal mol^{-1} $Å^2$ force constant were applied throughout the minimization, heating and equilibration steps.

Molecular dynamics simulations

The CUDA accelerated version of PMEMD[7] available in Amber14 was used to produce a 100 ns long molecular dynamics trajectory of the CypA-**1** complex in the NTP ensemble at 300K and 1 atm, applying SHAKE to bonds involving hydrogen and using a timestep of 2 fs.

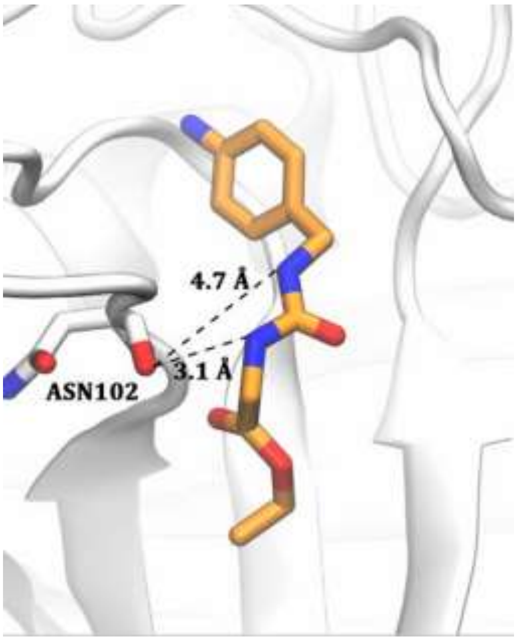

Figure S1. Representative snapshot from a MD simulation of CypA-**1** indicative of weakened hydrogen-bonding interactions between the urea nitrogen atom distal to the ester moiety of the ligand and the backbone carbonyl of Asn102.

Free energy calculations

Relative binding free energies of all tested compounds were estimated by alchemical free energy calculations[8]. A perturbation map was generated (Fig. S2 and Fig. S3) by manual connection of the ligands in both types of binding modes via multiple transformations. Simulations were performed using the SOMD (Sire–OpenMM) framework version 2017.3[9] on GeForce GTX465 and Tesla/M2090/K20 graphic cards. The number of equidistant λ windows used for each perturbation was varied between 9, 17 or 26 (values for each window between 0.0000 - 1.0000), based on the chemical similarity of the starting and the final compounds. Before the production run, all systems were energy minimized for 1000 steps and then re-equilibrated at the appropriate λ value for 20 ps. The total length of each simulation was 2 ns, and the perturbed energies were saved every 200 fs. A softcore potential and 2 fs timestep were used in all simulations and all not-perturbed hydrogen bonds were constrained using SHAKE. Simulations were performed in the NPT ensemble using the Andersen thermostat[10] and a Monte Carlo barostat. Periodic boundary conditions were also applied with a 10-Å cutoff for the non-bonded interactions. Electrostatic interactions were handled with an atom-based Barker-Watts reaction field.[11] The perturbed energies where post-processed using the MBAR estimator[12].Binding free energies were averaged and errors where estimated across three independent repeats for each forward and backward perturbation. Convergence was assessed by checking the consistency between binding free energies from forward and backward simulations, as well as the cycle closures in the perturbation network. Average binding free energies and standard errors of the mean for each free energy perturbation and cycle closures can be seen in Tables S3-S5. Binding free energies of all compounds relative to 1-BM1 in the ester series and 22-BM1 in the bromo-arylpyrrolidone series were subsequently obtained by summing all the relative binding free energies along all possible paths that connect the final and reference compounds and errors were propagated across the same path. The final reported relative free energy is the average across all unique paths, weighted by the uncertainties of each path such that more precise paths have a greater statistical weight. See ref[13] section ‘’Free energy analysis and convergence’’ for a more detailed description. All input files for the free energy calculations and a summary of the simulation output files are available online on a github repository at https://github.com/michellab/cyp-trivector .

BM1: Type I binding

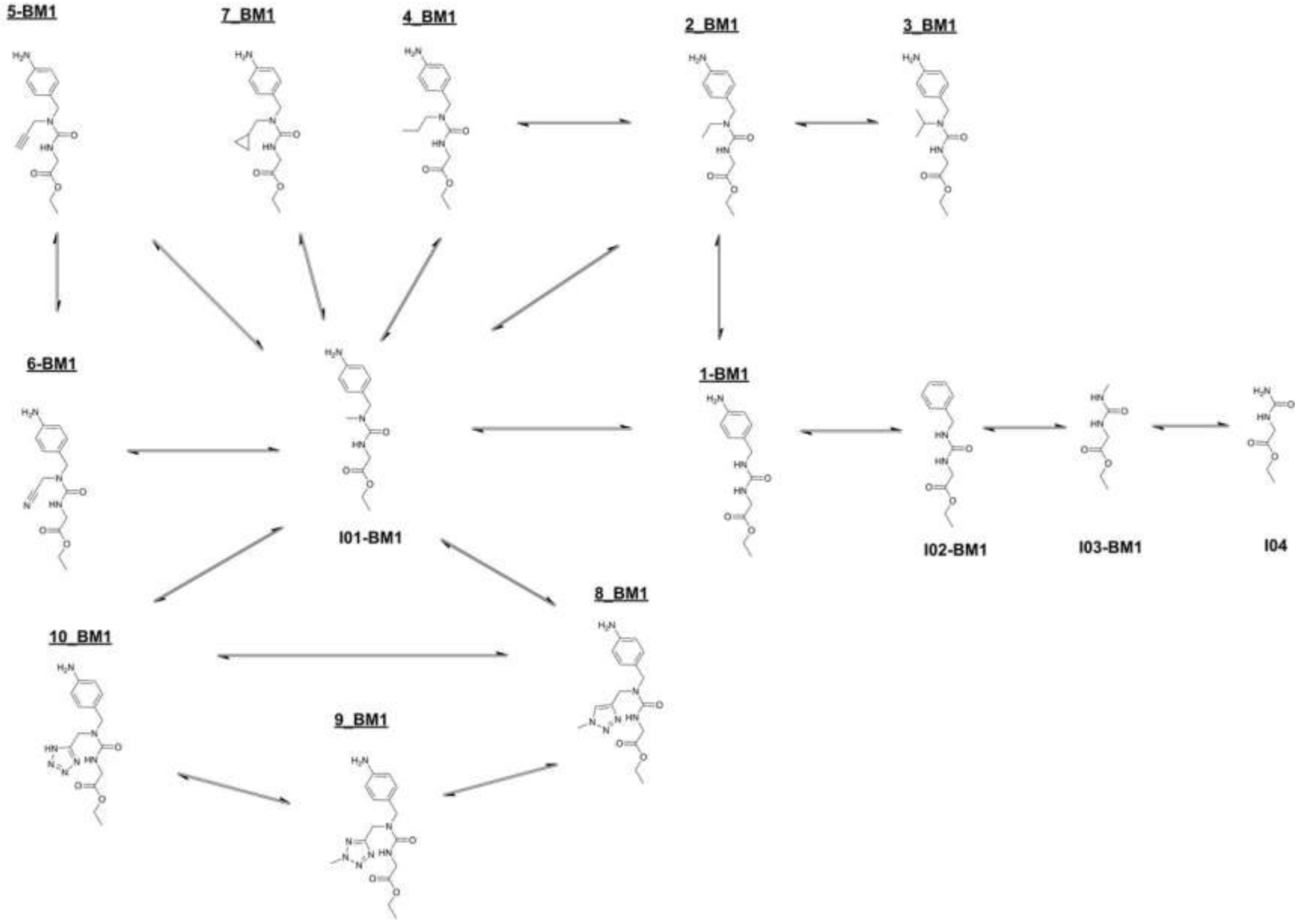

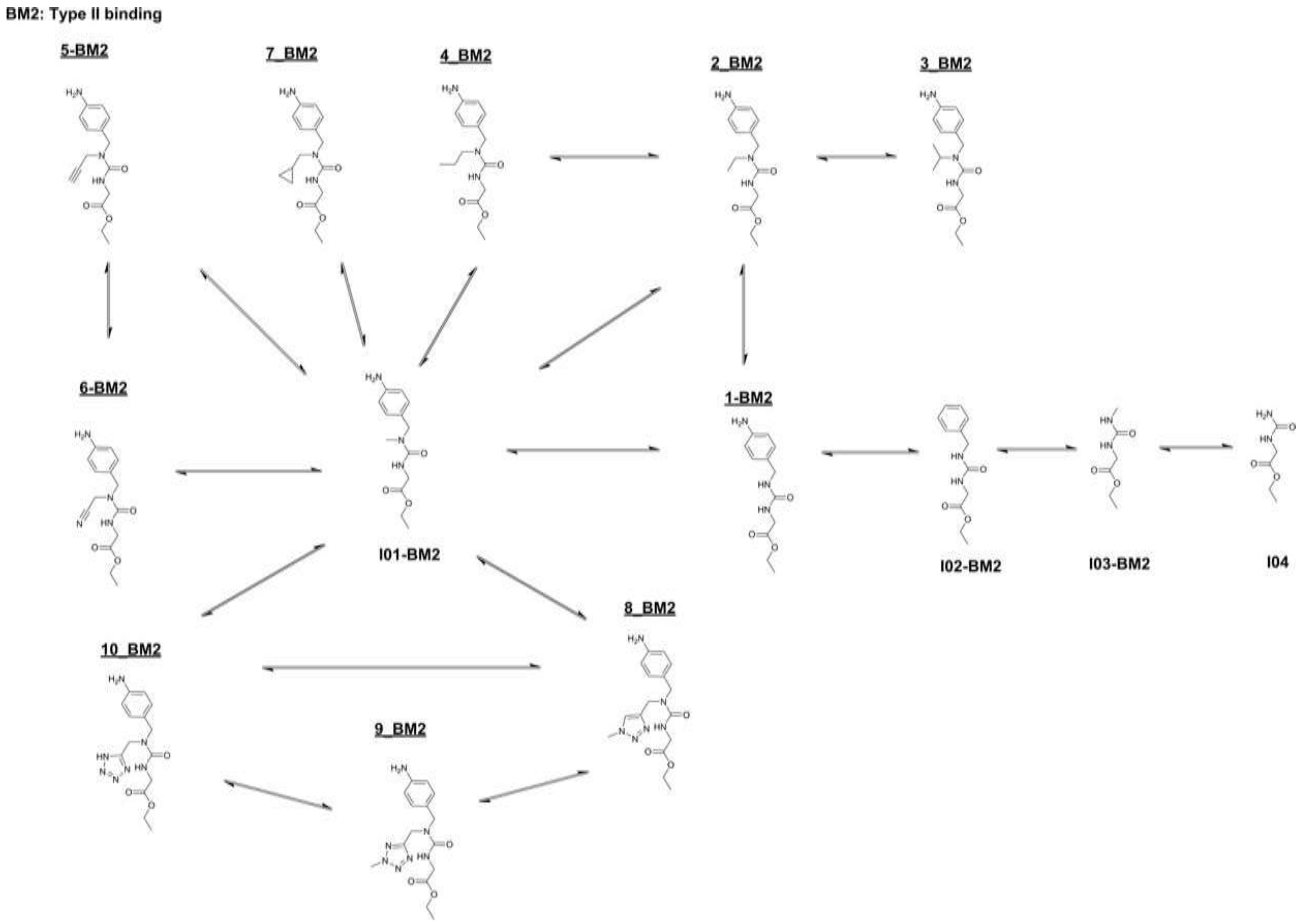

Figure S2. Perturbation map for FEP calculations in the ester series.

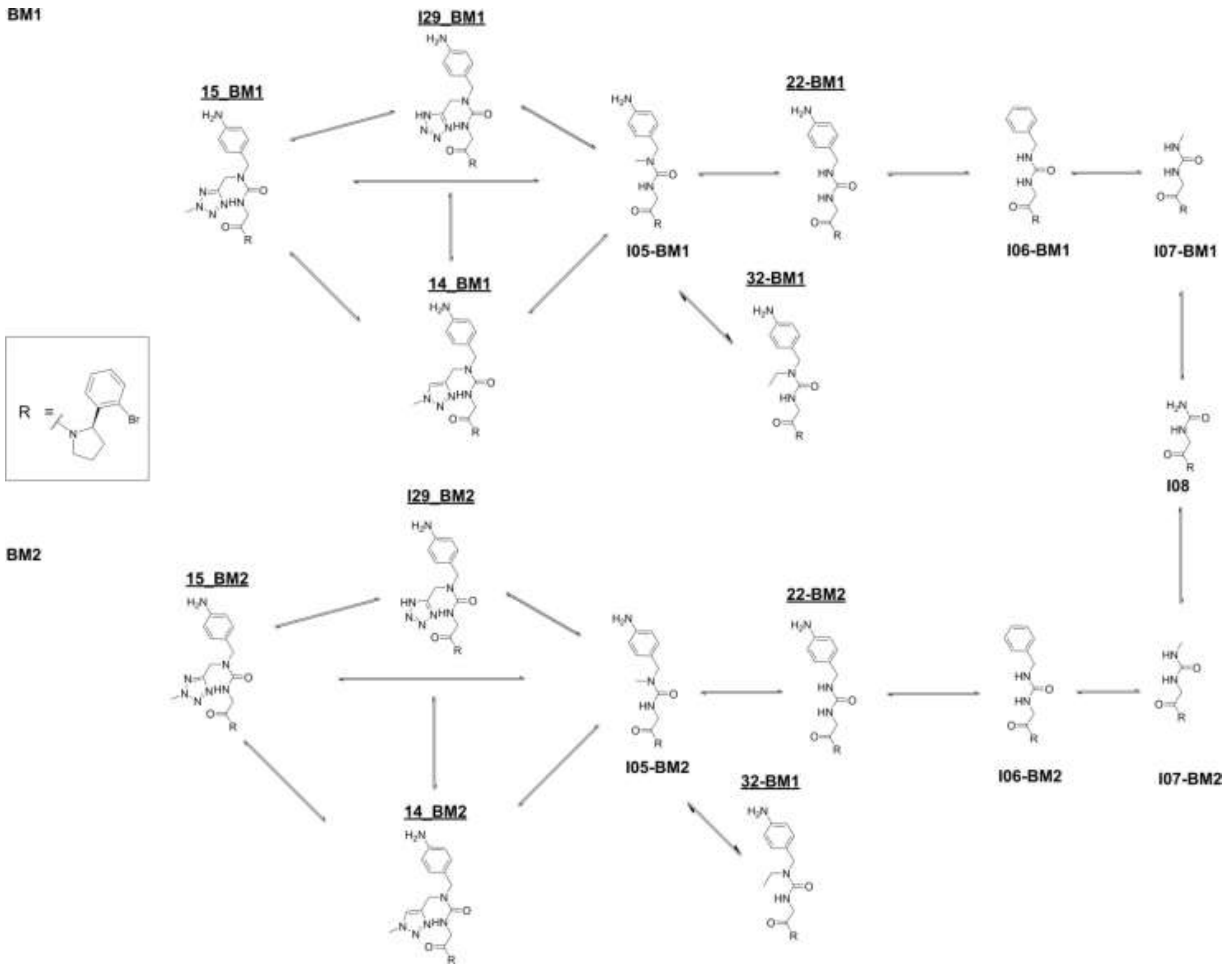

Figure S3. Perturbation map for FEP calculations in the bromo-arylpyrrolidone series.

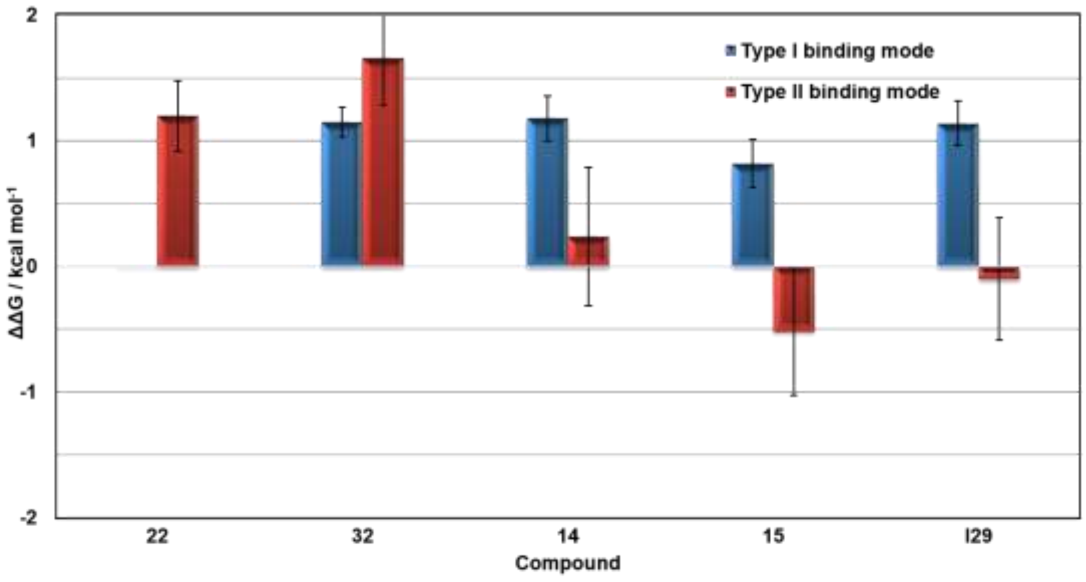

Figure S4. FEP calculated binding energetics for compounds **14** and **15** to CypA.

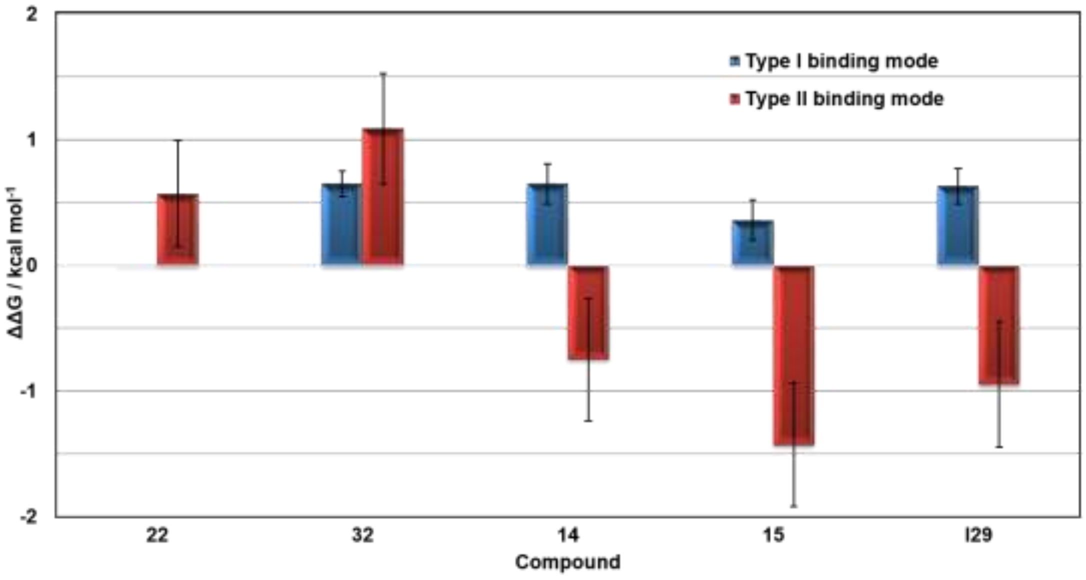

Figure S5. FEP calculated binding energetics for compounds **14** and **15** to CypD.

Table S3. Average ΔG ± 1σ for each FEP (averaged over three independent forward and three independent backward calculations) and cycle closure errors in the ester series.

L1	L2	ΔG	std
		kcal mol^{-1}	
10_BM2	9_BM2	-0.4	0.04
2_BM1	1_BM1	-0.75	0.09
2_BM1	4_BM1	-0.28	0.05
2_BM2	1_BM2	-0.68	0.2
2_BM2	4_BM2	-0.18	0.07
3_BM1	2_BM1	-2.03	0.18
3_BM2	2_BM2	-3.96	0.38
5_BM1	6_BM1	-0.15	0.02
5_BM2	6_BM2	-0.34	0.03
8_BM1	9_BM1	-0.29	0.01
8_BM1	10_BM1	-0.15	0.05
8_BM2	9_BM2	-0.94	0.04
8_BM2	10_BM2	-0.21	0.09
9_BM1	10_BM1	-0.05	0.14
I01_BM1	1_BM1	-0.75	0.1
I01_BM1	8_BM1	-0.51	0.09
I01_BM1	7_BM1	-0.29	0.04
I01_BM1	6_BM1	-0.24	0.03
I01_BM1	4_BM1	-0.17	0.06
I01_BM1	10_BM1	-0.13	0.18
I01_BM1	5_BM1	-0.07	0.05
I01_BM1	2_BM1	-0.04	0.12
I01_BM2	10_BM2	-2.46	0.19
I01_BM2	8_BM2	-1.9	0.23
I01_BM2	1_BM2	-1.86	0.22
I01_BM2	6_BM2	-0.88	0.01
I01_BM2	5_BM2	-0.63	0.05
I01_BM2	2_BM2	-0.4	0.04
I01_BM2	4_BM2	-0.32	0.11
I01_BM2	7_BM2	-0.31	0.04
I02_BM1	1_BM1	-1.36	0.06
I02_BM2	1_BM2	-0.3	0.07
I03_BM1	I02_BM1	-1.85	0.17
I03_BM2	I02_BM2	-1.64	0.23
I04	I03_BM1	-0.51	0.09
I04	I03_BM2	-0.43	0.08

Cycle closures	Binding mode	ΔΔG (kcal mol^{-1})
I01 - 10 - 9 - 8 - I01	1	0.72
I01 - 10 - 8 - I01	1	0.53
10 - 9 - 8 - 10	1	0.19
I01 - 1 - 2 - 4 - I01	1	-0.11
I01 - 1 - 2 - I01	1	0.04
I01 - 2 - 4 - I01	1	-0.15
I01 - 5 - 6 - I01	1	0.02
I01 - 10 - 9 - 8 - I01	2	-0.02
I01 - 10 - 8 - I01	2	-0.35
10 - 9 - 8 - 10	2	0.33
I01 - 1 - 2 - 4 - I01	2	-1.04
I01 - 1 - 2 - I01	2	-0.78
I01 - 2 - 4 - I01	2	-0.26
I01 - 5 - 6 - I01	2	-0.09

Table S4. Average ΔG ± 1σ for each FEP (averaged over three independent forward and three independent backward calculations) and cycle closure errors in the bromo-arylpyrrolidone series binding to CypA.

L1	L2	[illegible]	[illegible]
		kcal mol^{-1}	
14_BM1	15_BM1	-0.42	0.05
14_BM1	I05_BM1	-0.08	0.18
14_BM2	15_BM2	-0.72	0.06
14_BM2	I29_BM2	-0.36	0.17
32_BM1	I05_BM1	-0.07	0.03
I05_BM1	22_BM1	-1.08	0.12
I05_BM1	15_BM1	-0.52	0.1
I05_BM2	15_BM2	-2.28	0.44
I05_BM2	I29_BM2	-2.05	0.65
I05_BM2	14_BM2	-1.67	0.16
I05_BM2	22_BM2	-0.67	0.07
I05_BM2	32_BM2	-0.21	0.23
I06_BM1	22_BM1	-1.1	0.05
I06_BM2	22_BM2	-0.47	0.05
I07_BM1	I06_BM1	-1.97	0.08
I07_BM2	I06_BM2	-1.45	0.24
I08	I07_BM1	-0.43	0.07
I08	I07_BM2	-0.37	0.08
I29_BM1	15_BM1	-0.4	0.03
I29_BM1	I05_BM1	-0.37	0.14

I29_BM1	14_BM1	-0.02	0.02
I29_BM2	15_BM2	-0.41	0.08

Cycle closures	Binding mode	ΔΔG (kcal mol^{-1})
I05 - I29 - 15 - 14 - I05	1	0.31
I05 - I29 - 14 - I05	1	0.27
I05 - I29 - 15 - I05	1	0.49
I05 - 15 - 14 - I05	1	-0.18
I29 - 15 - 14 - I29	1	0.04
I05 - I29 - 15 - 14 - I05	2	-0.07
I05 - I29 - 14 - I05	2	-0.02
I05 - I29 - 15 - I05	2	-0.18
I05 - 15 - 14 - I05	2	0.11
I29 - 15 - 14 - I29	2	-0.05

Table S5. Average ΔG ± 1σ for each FEP (averaged over three independent forward and three independent backward calculations) and cycle closure errors in the bromo-arylpyrrolidone series binding to CypD.

L1	L2	ΔG	std
		kcal mol^{-1}	
14_BM1	15_BM1	-0.44	0.03
14_BM1	I05_BM1	-0.3	0.12
14_BM1	I29_BM1	-0.07	0.06
14_BM2	15_BM2	-0.65	0.03
14_BM2	I29_BM2	-0.23	0.09
22_BM2	I06_BM2	-0.02	0.04
32_BM2	I05_BM2	-0.03	0.07
I05_BM1	22_BM1	-0.74	0.04
I05_BM1	15_BM1	-0.58	0.08
I05_BM1	I29_BM1	-0.32	0.24
I05_BM1	32_BM1	-0.09	0.1
I05_BM2	I29_BM2	-2.41	0.14
I05_BM2	15_BM2	-2.06	0.26
I05_BM2	14_BM2	-1.81	0.23
I05_BM2	22_BM2	-0.49	0.12
I06_BM1	22_BM1	-0.4	0.09
I07_BM1	I06_BM1	-1.83	0.23
I07_BM2	I06_BM2	-1.19	0.27
I08	I07_BM2	-1.17	0.15
I08	I07_BM1	-0.68	0.14
I29_BM1	15_BM1	-0.26	0.04

I29_BM2	15_BM2	-0.3	0.06

Cycle closures	Binding mode	ΔΔG (kcal mol^{-1})
I05 - I29 - 15 - 14 - I05	1	-0.44
I05 - I29 - 14 - I05	1	-0.55
I05 - I29 - 15 - I05	1	0
I05 - 15 - 14 - I05	1	-0.44
I29 - 15 - 14 - I29	1	0.11
I05 - I29 - 15 - 14 - I05	2	-0.25
I05 - I29 - 14 - I05	2	-0.37
I05 - I29 - 15 - I05	2	-0.65
I05 - 15 - 14 - I05	2	0.4
I29 - 15 - 14 - I29	2	0.12

Protein Expression and Purification

The plasmids of HisCypA, HisCypB and HisCypD were provided by the Edinburgh Protein Production Facility (EPPF). Protein expression and purification protocols were slightly modified from Wear et al. for their usage in ITC and X-ray studies.[14]

PEP1 - HisCypA and His-CypD transformation and expression

Recombinant Cyclophilins (Cyps) were produced in C41 BL21(DH3) *E. coli* cell lines (Lucigen, Middleton, WI, USA). Briefly, 1 µL of the stock plasmids were added to the competent cells, left on ice for about half an hour and after a two-minute heat shock at 42ºC were incubated by shaking (250 rpm) in SOC media (500 µL) at 37ºC for 45 minutes. They were left overnight to colonize in agar plates (100 µL/plate) containing carbenicillin (100 µg mol^{-1}). After this a single colony was picked and grown for six hours in LB media. Subsequently, 20 % v/v glycerol was added and these glycerol stocks were used in future reference.

A 100 mL pre-culture was left overnight in LB media using a glycerol stock and carbenicillin as antibiotics (100 µg mol^{-1}). The cultures were centrifuged for five minutes at 1,500 g and new 500 mL cultures were made by transferring the cell pellets, adding carbenicillin and incubating by shaking (250 rpm) until OD_{600} 0.6 – 0.8 at 37ºC and then induced at 30 ºC with 0.5 mM IPTG for four hours. Finally, the cultures were pelleted by centrifugation at 8,000 g for 20 minutes at 4 ºC prior to cell lysis.

PEP2 - Protein Purification

All purifications were performed on an ÄKTA Pure (GE Healthcare) equipment at 4 ºC. Prior to purification cell pellets were lysed using protease inhibitors (Roche) in loading buffer (20 mM phosphate, 300 mM NaCl, 20 mM imidazole, pH 7.4) by a double passage on a Constant Systems Cell Disruptor (1.1 kW TS Benchtop) at 22 kpsi followed by one hour centrifugation at 4 ºC (55,000 g). A two-step purification protocol was used in all cases, i.e. Immobilized Metal Ion Affinity Chromatography (IMAC) and Size Exclusion Chromatography (SEC) using the HiTrap IMAC FF 5 mL and the HiLoad Superdex 75 pg 16/60 columns, respectively. The buffer used in the SEC purification step was similar to the ITC buffer and for the IMAC elution 20 mM phosphate, 300 mM NaCl, 500 mM imidazole, pH 7.4. Representative gels are depicted in Figure S6.

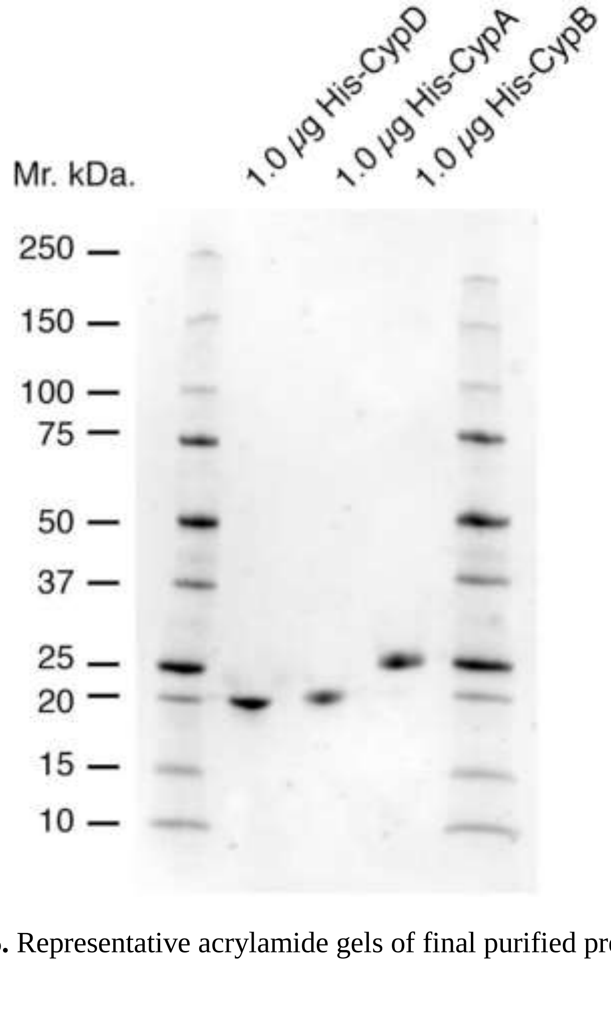

Figure S6. Representative acrylamide gels of final purified preparations

PEP3 - HisTag cleavage

Protein His-Tag was cleaved for further use of the protein in ITC and X-ray studies, whereas for the SPR experiments the protein was uncleaved. Proteins were desalted to cleavage buffer (100 mM Tris, 100

mM NaCl, pH 7.5) using a HiPrep 26/10 desalting column prior to the addition of TEV protease (200 ng TEV / 40 µg protein). Samples were left incubating at 30 °C for about four hours and the cleaved His-tag was removed by IMAC. At the end of each purification the purity of the fractions was tested by using precast gels (Biorad®) in Tris/Glycine/SDS, pH 8.3 buffer. A representative LC-MS spectrum of purified His-tag cleaved CypA is shown in Figure S7.

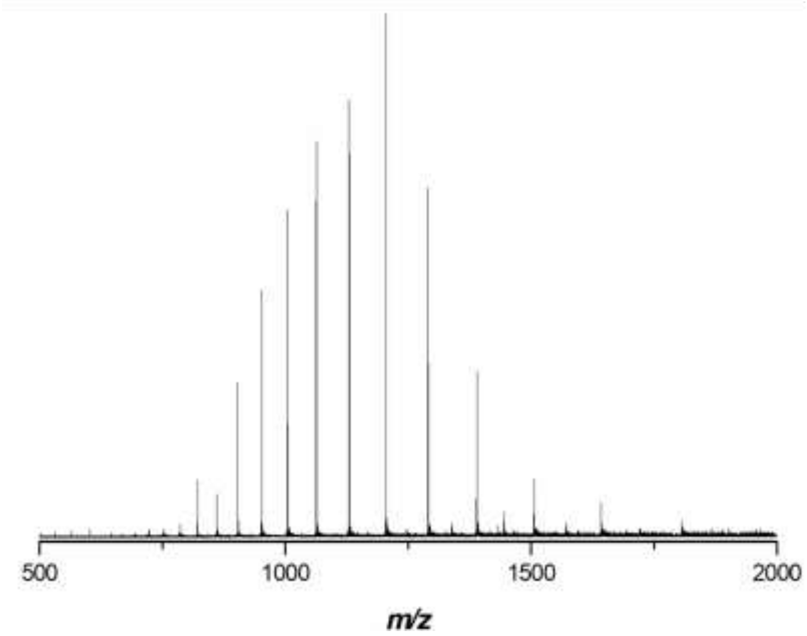

Figure S7. Positive mode mass spectrum acquired by LC-MS of wt-CypA following His tag cleavage. A charge state distribution can be seen, and the $[M+21H]^{+21}$ and $[M+20H]^{+20}$ ions have been highlighted. The deconvoluted average mass was calculated to be 18070.33 Da, this corresponds well with the calculated theoretical average mass of 18070.39 Da.

PEP4 - Miscellaneous

The molecular weights of HisCypA and free CypA are 20.893 and 18.070 kDa, respectively. Protein concentration was determined by measuring the absorbance at 280 nm and the extinction coefficients 14440 and 8480 M^{-1} cm^{-1}, respectively.

Isothermal Titration Calorimetry (ITC)

ITC1 - Ester series: Instrument and experimental setup

All ITC experiments for the ester series compounds were carried out at 25 °C on a MicroCal Auto iTC200 (GE Healthcare) instrument. The buffer used in the titrations of the compounds belonging to the ester series (**1 – 12**) was 50 mM phosphate buffer, pH 6.5 and the concentration of DMSO was 2% v/v for all the compounds, unless otherwise stated in Table S1. Final compound solutions were heated to 65 °C and/or sonicated prior to the experiment. Each experiment consisted of an initial injection of 0.4 µL followed by nineteen 2 µL injections and in most of cases for these compounds, the "continue injections" protocol was used leaving the cell intact and performing a second series of titrations using the above protocol to achieve saturation. Control experiments were performed, where each compound was titrated into buffer and when small amount of heat was detected due to heat of dilution, it was subtracted when processing the data using a linear fit method. A more detailed setup for each experiment is depicted in Table S6. In all cases the first injection was omitted from the data processing. All data were analysed using the MicroCal PEAQ-ITC Analysis software. Because most of these compounds lie in the low-mid micromolar range and the *c* value (Wiseman constant) is very small, a fixed stoichiometry to 1 was applied during the non-linear regression of the raw data for fitting the data.[15] Fig. S8 shows selected ITC thermograms of the ester series compounds. Compound **1** was run as a control at the end of each experiment to verify that CypA remained active during the duration of the experiment.

Table S6. ITC set-up details with the respective dissociation constants (K_d) of compounds 1 – 12.

	**[Cell] (µM)[#]**	**[Syringe][#] (mM)**	**[DMSO] (% v/v)**	**Continuous Injections**	**K_d (µM)[$,&]**
**1**	50	2	2		35.1 ± 0.6
**2**	50	3	---		200 ± 5
**3**	50	2	---		n.b
**4**	50	2	2	✓	266 ± 3

**5**	50	0.5	2	✓	>1000
**6**	100	2	2	✓	993 ± 20
**7**	50	0.5	2		n.b
**8**	30	0.6	2	✓	135 ± 6
**9**	30	0.6	2	✓	184 ± 13
**10**	30	0.6	---	✓	139 ± 4
**11**	25	0.5	2		n.b
**12**	25	0.5	2		n.b

[#]CypA in cell and ligands in syringe, except for compounds **11** and **12** that are reverse titrations. [$]$K_d$ values coming from single experiments. [&]Uncertainties as resulted from the fitting to the one-site-binding model. n.b: no binding

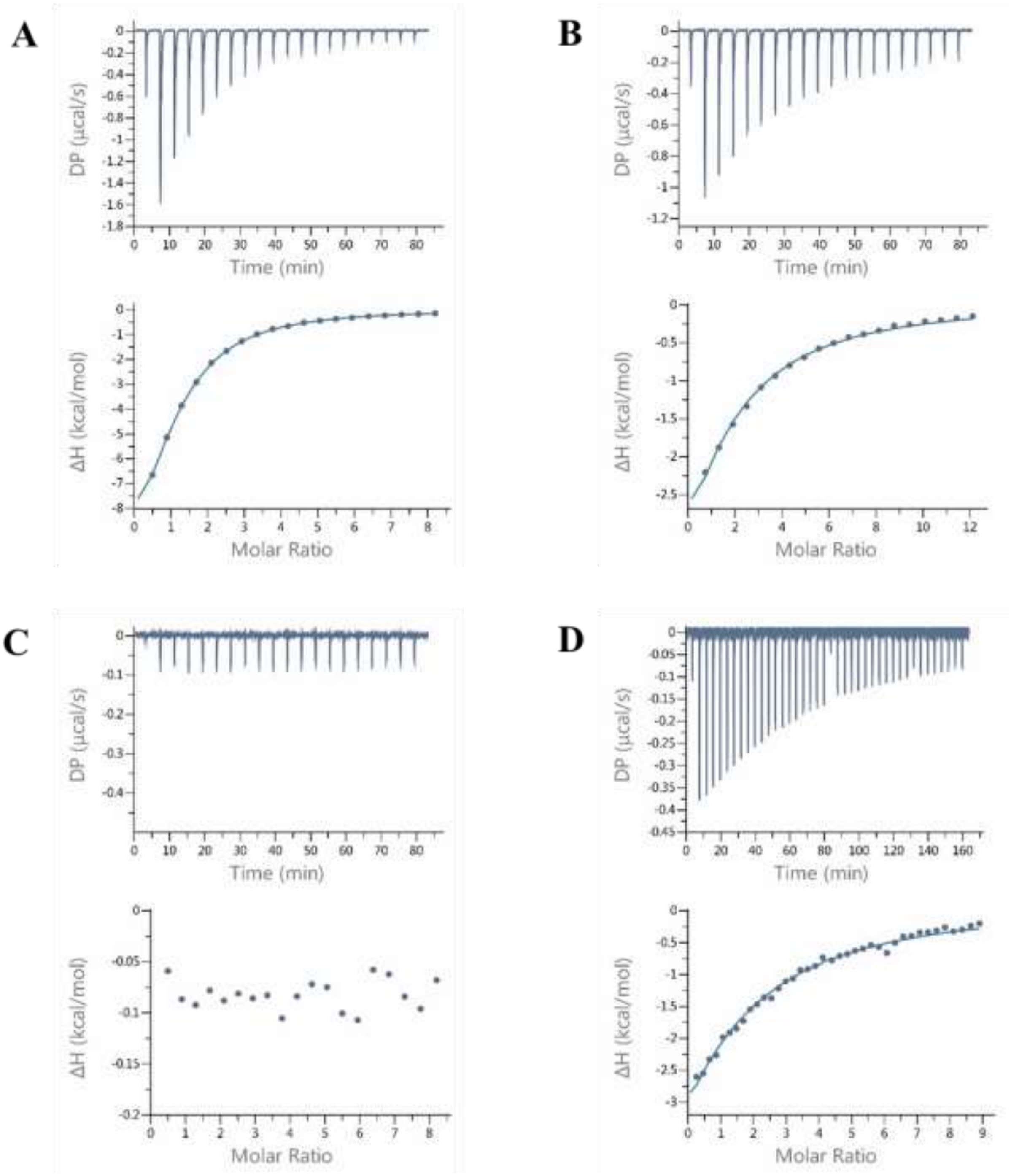

Figure S8. Representative graphs from ITC titrations with compounds **1** (A), **2** (B), **3** (C) and **10** (D).

ITC2 - Bromo-aryl-pyrrolidine series: Instrument and experimental setup

Compound **15,** that belong to the bromo-arryl-pyrrolidine series, was tested using a reverse titration using a competition-based method as previously described with CsA. [16] The buffer used for this titrations was: PBS, 0.05% v/v P20 surfactant, 50 µM EDTA in presence of 2% v/v EtOH and the pH was set to 7.4. Cyclophilin A (60 µM) was titrated into 4 µM CsA in presence of 10 µM compound **15** in cell using a 15-

injection protocol (Fig. S9B). A control experiment comprising of a titration of 60 μM CsA into 4 μM CsA using the same instrument parameters was performed (Fig. S9A).

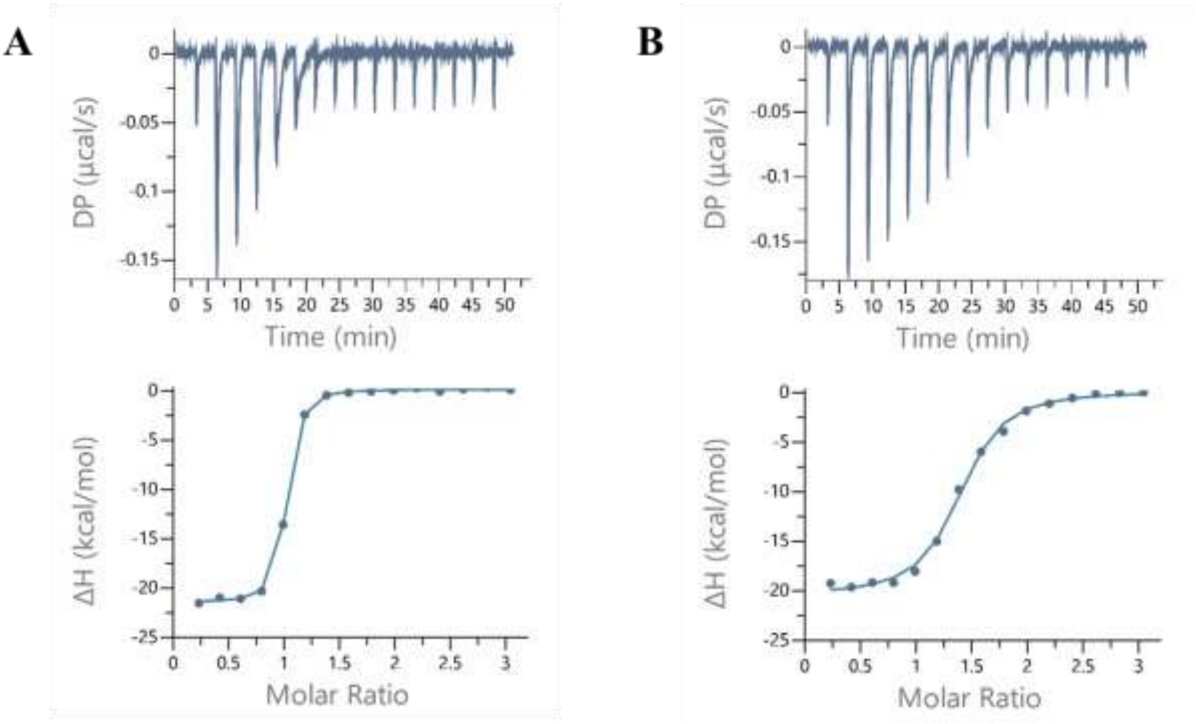

Figure S9. Representative graphs from ITC titrations with **CsA** (A), **15** (B).

Surface Plasmon Resonance (SPR)

SPR1 - SPR equipment and reagents

SPR measurements were performed on a BIAcore T200 instrument (GE Healthcare). Ni^{2+}-nitrilotriacetic acid (NTA) sensor chips, 1-ethyl-3-(3- diaminopropyl) carbodiimide hydrochloride (EDC) and Nhydroxysuccinimide (NHS) were purchased from GE Healthcare.

SPR2 - Immobilization and covalent stabilizations of His-Cyps

Pure His-cyclophilins were immobilized and covalently stabilized on the NTA sensor chip according to the protocol described [Wear *et al.*, 2017, FEBS OpenBio], using 200 nM concentrations of each protein, in Running Buffer (PBS, pH 7.4; 0.05% surfactant P20, 2% v/v ethanol; 50 µM EDTA), at 30 µl min^{-1} with 60 second contact times on the activated NTA surfaces. This gave signals of 1,921 RU for His-CypA, 1932 RU for

His-CypB and 1,397 RU for His-CypD. Specific surface protein activity was assayed by passing saturating amounts of CsA (2 µM) in Running Buffer over these surfaces; values of 94.1 %, 95.5 % and 95.6 % activity were obtained for His-CypA, -B and –D, respectively.

SPR3 - Kinetic titration experiments

CsA controls: Single cycle kinetic titration binding experiments were performed using SPR in triplicate at 25˚C. 3-fold dilution concentration series of CsA, ranging from 2.45 nM to 200 nM, in Running Buffer (PBS, pH 7.4, 50 µM mM EDTA; 0.05 % v/v surfactant P20; 2 % v/v ethanol), were injected over the sensor surface, at 100 µl.min^{-1} with a 90 s contact time and a 90 s dissociation time. The sensor surface was regenerated between experiments by dissociating any formed complex in running buffer for at least 1,200 seconds. The apparent on-rate (k+) and off-rate (k-) constants and the equilibrium dissociation constant (K_d) were calculated from reference corrected sensorgrams by global fitting of a 1:1 binding model, including a mass transport term, using analysis software (v.2.02, GE Healthcare) provided with the BIAcore T200 instrument. Typical results are shown in Figure S10.

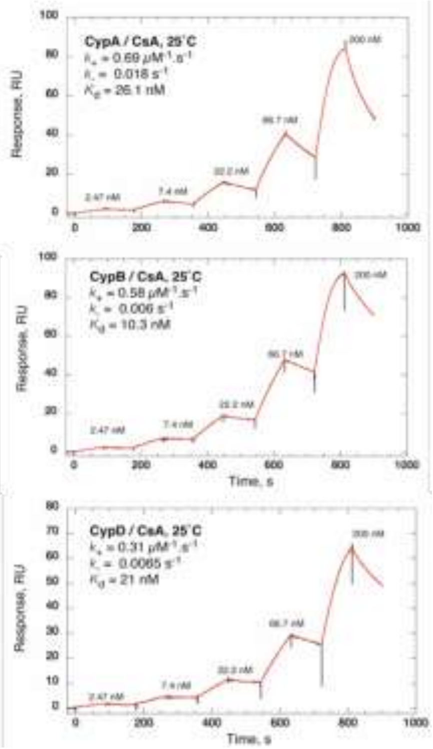

Figure S10. Representative SPR single cycle kinetic titration experiments with CsA.

Assays of compounds 13-16: Kinetic titration binding experiments were performed in triplicate at 25˚C. 2-fold dilution concentration series of the compounds, ranging from 0.0195 µM to 20 µM, in Running Buffer (PBS, pH 7.4, 50 µM mM EDTA; 0.05 % v/v surfactant P20; 2 % v/v ethanol), were injected over the sensor surface, at 100 µl.min^{-1} with a 15 s contact time and a 600 s dissociation time. The sensor surface was regenerated between experiments by dissociating any formed complex in running buffer for at least a further 600 seconds. The apparent on-rate (k+) and off-rate (k-) constants and the equilibrium dissociation constant (K_d) were calculated from reference corrected sensorgrams by global fitting of a 1:1 binding model, including a mass transport term, using analysis software (v.2.02, GE Healthcare) provided with the BIAcore T200 instrument. Typical results are shown in respective figures S11-S13 for HisCypA, -B and –D.

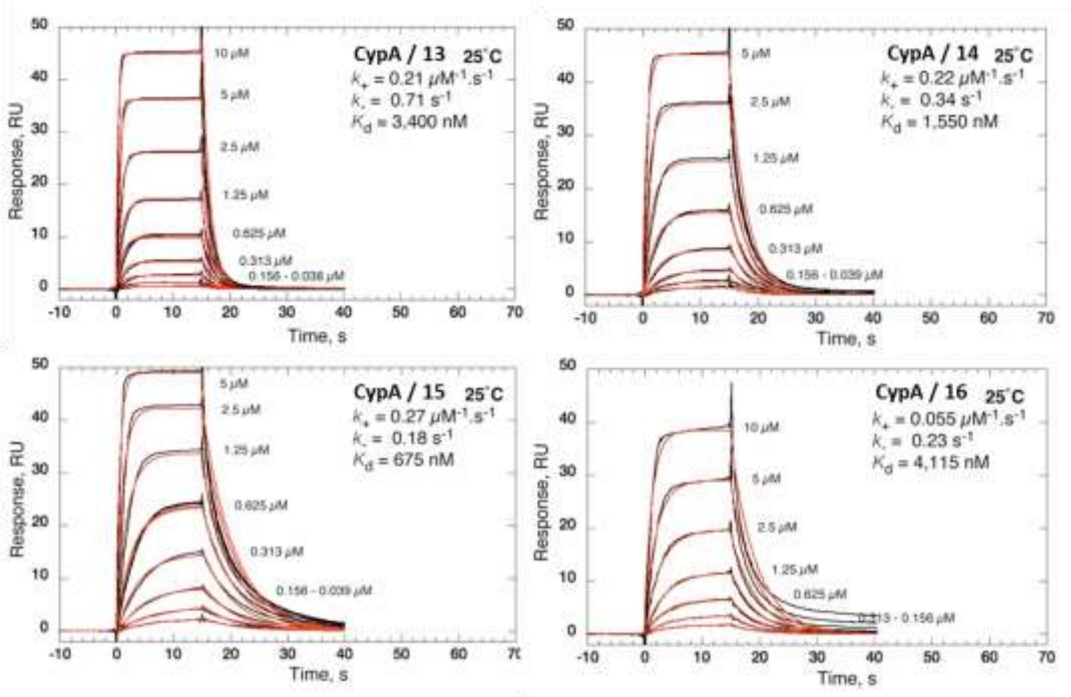

Figure S11. Characterisation of the interaction of His-CypA with compounds **13-16** using BIAcore T200.

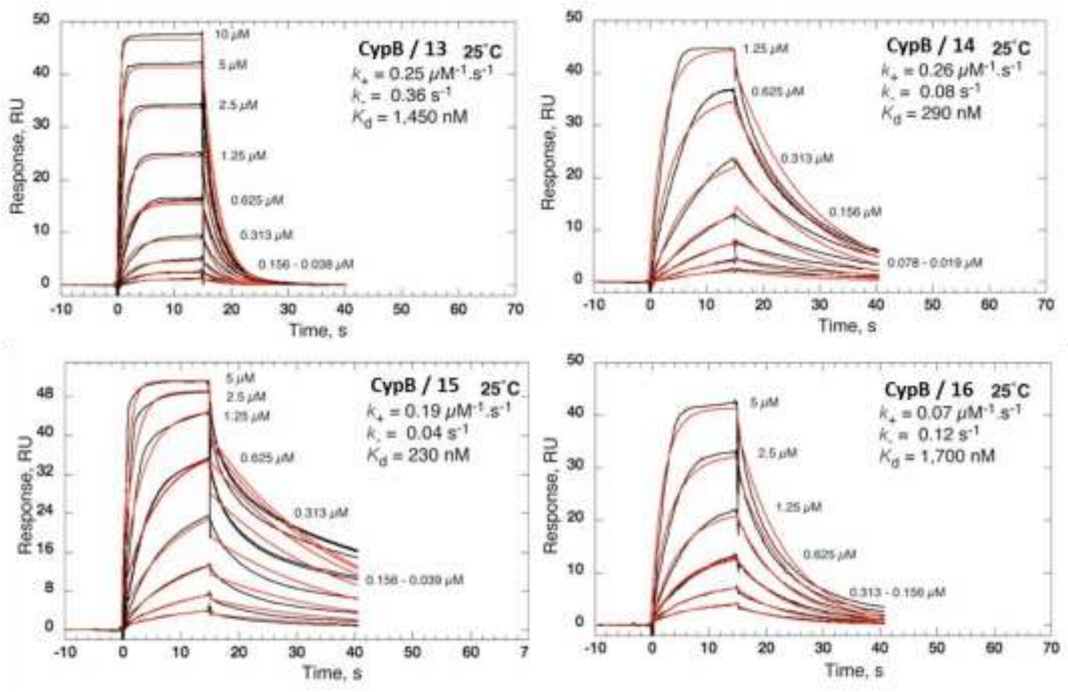

Figure S12. Characterisation of the interaction of His-CypB with compounds **13-16** using BIAcore T200.

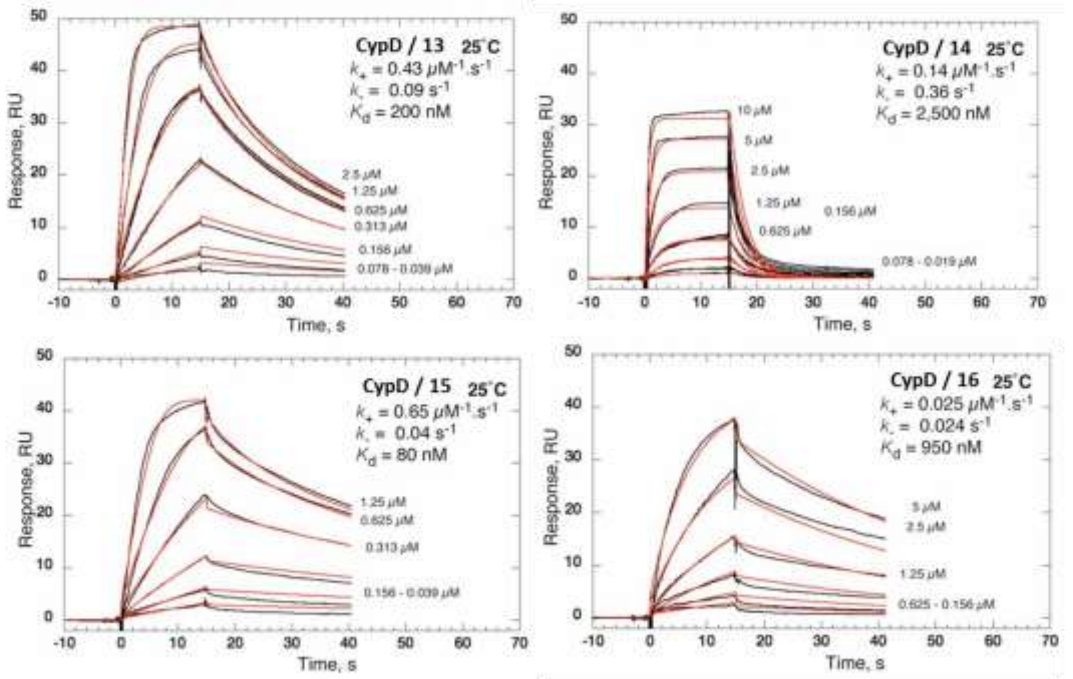

Figure S13. Characterisation of the interaction of His-CypD with compounds **13-16** using BIAcore T200.

X-ray diffraction experiments

Purified and his-tag-cleaved CypA was buffer-exchanged into PBS and concentrated to ~29 mg ml^{-1}. For crystallisation 1μL of protein was mixed with an equal volume of the well solution, consisting of 100mM Tris-HCl pH8.0 and 20-22% v/v PEG 8000, and crystal formation came about after equilibration overnight in 6 °C by vapour diffusion using the hanging drop method over 1mL of the same well solution. Apo CypA crystals were soaked overnight into different ligand solution consisting of 100mM Tris-HCl pH 8.0, 35% w/v PEG 8000, 5% v/v Glycerol, 5% v/v DMSO and 5mM ligand, before flash frozen into liquid nitrogen. X-ray data were collected at the Diamond synchrotron-radiation facility in Oxford-shire, England at 100K. Structures were solved by molecular replacement using DIMPLE from the CCP4i suite.[17] Modelled structures were visualised and manually adjusted as needed using Coot10 and further refined using REFMAC5 from CCP4i.[18] X-ray diffraction and refinement statistics are reported in Table S7.

Table S7. X-ray refinement statistics

**Protein**	CypA	CypA	CypA	CypA	CypA
**Compound number**	**2**	**4**	**5**	**7**	**8**
**PDB ID**	6GJJ	6GJM	6GJY	6GJP	6GJI
**Data collection and processing**					
High resolution limit	1.38 (1.38)	1.35 (1.35)	1.29 (1.29)	1.94 (1.94)	1.56 (1.56)
Low resolution limit	46.11 (3.74)	34.04 (1.37)	43.99 (1.31)	54.28 (1.97)	46.28 (1.62)
Completeness	99.80 (99.8)	99.87 (100)	98.89 (98.75)	99.6 (99.7)	99.9 (100)
Multiplicity	6.30 (6.4)	6.20 (6.30)	6.0 (5.1)	5.7 (5.8)	6.3 (6.6)
I/sigma	16 (1.3)	11.6 (2.2)	12.1 (1.2)	11.0 (4.0)	13.5 (2.4)
Rmerge	0.045 (1.56)	0.07 (0.49)	0.06 (1.29)	0.16 (1.11)	0.07 (0.62)
Unit cell dimensions:					
a (Å)	42.99	42.74	42.59	42.49	42.62
b (Å)	53.88	54.35	52.84	54.28	54.53
c (Å)	85.15	87.31	87.98	88.18	87.48
α (°)	90.00	90.00	90.00	90.00	90.00
β (°)	90.00	90.00	90.00	90.00	90.00
γ (°)	90.00	90.00	90.00	90.00	90.00
Space group	$P\ 2_1\ 2_1\ 2_1$	$P\ 2_1\ 2_1\ 2_1$	$P\ 2_1\ 2_1\ 2_1$	$P\ 2_1\ 2_1\ 2_1$	$P\ 2_1\ 2_1\ 2_1$
Total observations	272156 (13600)	280231 (14056)	300562 (12564)	89117 (4465)	176212 (8865)
Total unique	43264 (2113)	45033 (2216)	50202 (2449)	15641 (765)	27755 (1350)

Refinement statistics					
No. of residues per chain (No. of chains)	165	165	165	165	165
No. of ligands	1	1	1	1	1
No. of waters	242	253	297	182	225
Percentage of free reflections	4.86	5.10	5.05	5.20	5.16
R factor	0.15	0.16	0.16	0.17	0.15
R free	0.18	0.18	0.18	0.23	0.19
Rms BondLength	0.02	0.01	0.02	0.02	0.02
Rms BondAngle	1.65	1.29	2.08	1.75	1.99
**Ramachandran plot**					
Favoured (%)	94.3	96.2	94.7	93.7	95.6
Allowed (%)	5.7	3.8	5.3	6.3	4.4
No. of outliers	0	0	0	0	0

**Protein**	CypA	CypA	CypA
**Ligand**	**9**	**10**	**15**
**PDB ID**	6GJR	6GJL	6GJN
**Data collection and processing**			
High resolution limit	1.69 (1.69)	1.16 (1.16)	1.70 (1.70)
Low resolution limit	38.25 (1.72)	31.49 (1.18)	45.74 (1.74)
Completeness	99.67 (99.82)	99.8 (99.6)	99.6 (98.91)
Multiplicity	5.4 (4.6)	6.00 (4.20)	9.0 (7.4)
I/sigma	8.1 (0.7)	19.0 (1.2)	8.3 (4.2)
Rmerge	0.10 (1.72)	0.03 (1.17)	0.18 (0.60)
Unit cell dimensions:			
a (Å)	42.51	43.10	41.88
b (Å)	53.17	54.02	52.96
c (Å)	87.68	88.64	88.86
α (°)	90.00	90.00	90.00
β (°)	90.00	90.00	90.00
γ (°)	90.00	90.00	90.00
Space group	$P\ 2_1\ 2_1\ 2_1$	$P\ 2_1\ 2_1\ 2_1$	$P\ 2_1\ 2_1\ 2_1$
Total observations	122679 (5188)	434385 (14999)	205259 (8703)
Total unique	22862 (1122)	72239 (3537)	22682 (1178)
**Refinement statistics**			
No. of residues per	165	165	165

chain (No. of chains)			
No. of ligands	1	1	1
No. of waters	161	298	241
Percentage of free reflections	5.16	4.92	5.12
R factor	0.20	0.17	0.17
R free	0.26	0.19	0.20
Rms BondLength	0.02	0.01	0.02
Rms BondAngle	2.03	1.49	2.10
**Ramachandran plot**			
Favoured (%)	95.0	95.5	95.0
Allowed (%)	5.0	4.5	5.0
No. of outliers*	0	0	0

Cell assays

Materials and Reagents

Dulbecco's Modified Eagle's Medium (DMEM, with high glucose, sodium bicarbonate and L-glutamine) was purchased from Sigma-Aldrich and fetal bovine serum (FBS) was purchased from Thermo-Fisher Scientific. Draq 7 was purchased from New England Biolabs. 384-well microclear tissue culture-treated plates for microscopy were purchased from Greiner Bio-One.

Cyclophilin A antibody (rat polyclonal), cyclophilin B antibody (rabbit monoclonal) and GAPDH antibody (rabbit monoclonal) were purchased from New England Biolabs. Cyclophilin D antibody (mouse monoclonal) and mammalian protein extraction reagent (M-PER) was purchased from Thermo-Fisher Scientific. Complete EDTA-free protease inhibitor and phosSTOP phosphatase inhibitor were purchased from Roche. IRDye 800CW goat anti-rabbit, IRDye 800CW goat anti-mouse antibodies and IRDye 680RD were purchased from Li-Cor BioSciences. 4-15% mini protean TGX stain-free gels, 10X tris/glycine/SDS PAGE buffer and Transblot Turbo Midi nitrocellulose transfer packs were purchased from Bio-Rad Laboratories.

Cell Culture

The tumorigenic, breast, epithelial adenocarcinoma cell lines MDA-MB-231_NLG and the normal, lung fibroblast cell line, IMR90 were cultured as adherent monolayers in DMEM with 10% volume FBS in an atmosphere with 5% CO_2 and 95% humidity and were routinely sub-cultured upon reaching 80-90% confluence. MDA-MB-231_NLG cells are a variant of MDA-MB-231 cells, expressing nuclear-restricted green fluorescent protein; they were produced by stable transduction of MDA-MB-231 cells with NucLight Green lentivirus (Essen Bioscience), following the manufacturer's protocol.

Image-based Cell Viability Assays

Cells were seeded at a density of 500 cells per well in cell culture medium in 384-well cell culture plates and allowed to adhere overnight (about 16 hours), incubated in a humidified atmosphere with 5% CO_2. Subsequently, cell culture medium was refreshed, supplemented with Draq 7 (3μM final concentration) and test compound at the indicated concentrations with three wells being treated for each condition tested. Cells were then returned to the cell culture incubator and imaged with a 10X objective every 3 hours for 120 hours using an IncuCyte ZOOM microscope from Essen Bioscience.

Using the IncuCyte ZOOM software, custom image analysis procedures were developed and applied for each cell line to determine cell confluency, cell number and number of dead cells over the time course of the experiment. Phase contrast was used to determine relative area of each image occupied by cells (confluency), while green nuclear counts were used to determine number of MDA-MB-231_NLG cells and red nuclear counts were used to determine number of dead (Draq7-positive) cells.

Cell viability was determined relative to vehicle-treated (0.1% DMSO) controls using the GI_{50} method established by the National Cancer Institute with GI_{50} values (concentration of compound causing 50% growth inhibition) being determined by fitting non-linear regression curves to the data and extrapolating the required values using GraphPad Prism 6. Statistical analyses in cell viability assays to compare the effect of compound treatment to treatment with vehicle were performed using GraphPad Prism 6 (2-way ANOVA with Bonferroni correction post hoc).

Western Blotting

Cell were lysed with ice-cold MPER supplemented with protease and phosphatase inhibitor cocktails. Clarified lysates were resolved on 4-15% Trid-glycine gels by SDS-PAGE and total protein transferred to nitrocellulose membrane. Membranes were blocked with Li-Cor Buffer, probed with appropriate primary antibodies overnight, followed by washing and probing with appropriate fluorescence-conjugated secondary antibodies. Membranes were imaged and fluorescence intensity on the membranes recorded using the Li-Cor Odyssey CLx imager (Fig S14).

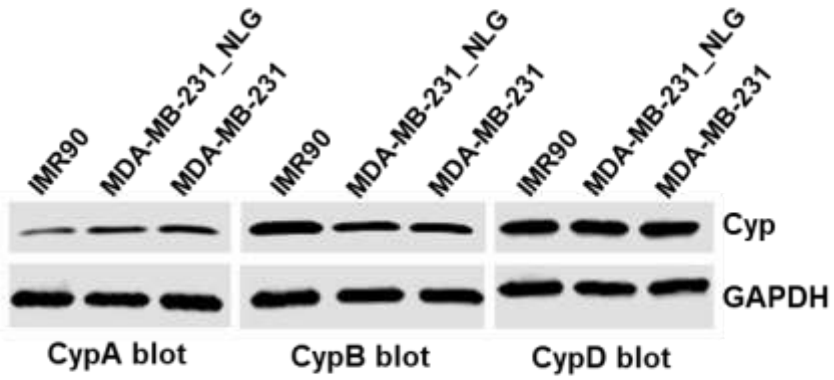

Figure S14. Verification by Western Blotting that the cell lines used for cell assays express CypA, CypB and CypD

^{1}H NMR and ^{13}C NMR of final products

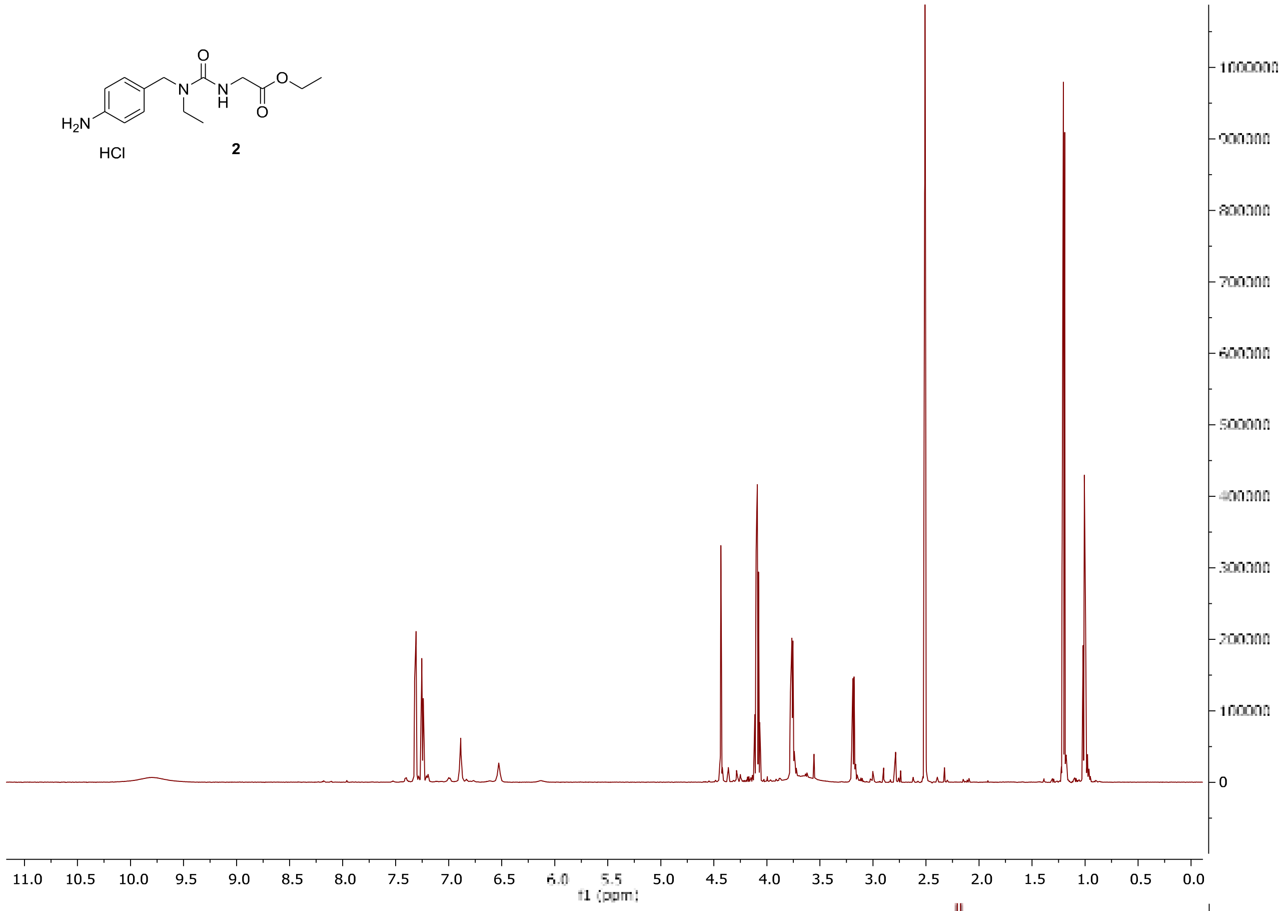
H2N
HCl
2
f1 (ppm)

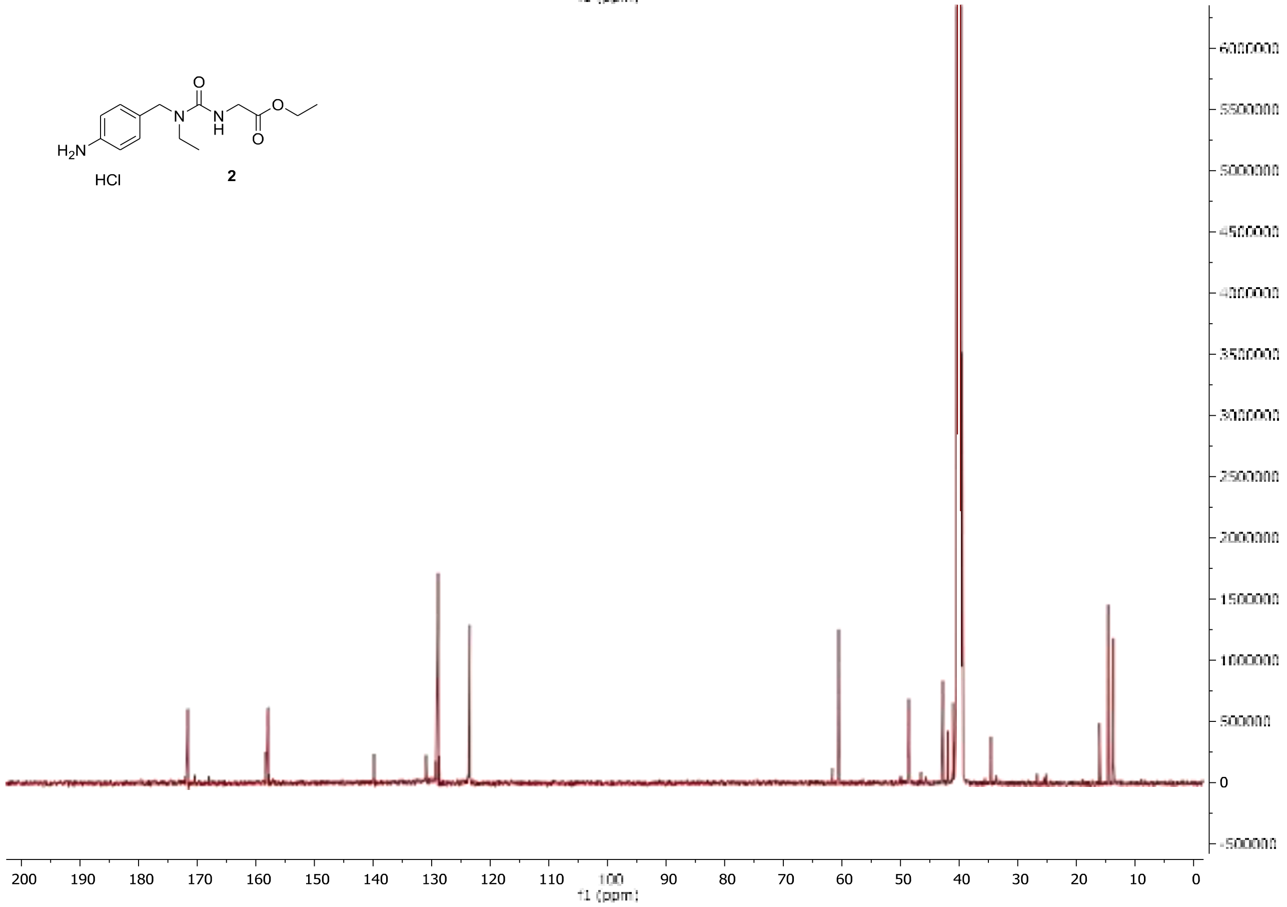
H2N
HCl
2
f1 (ppm)

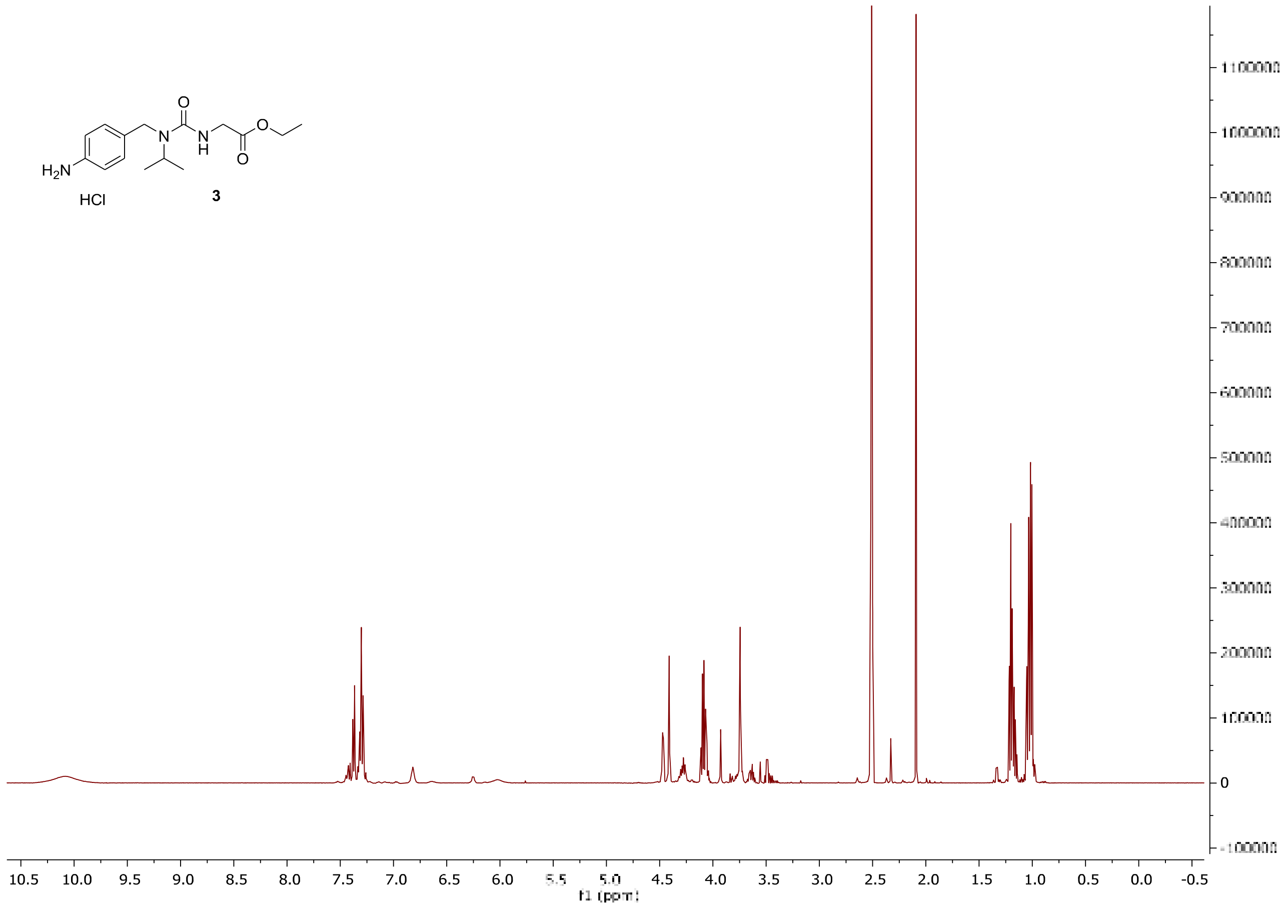

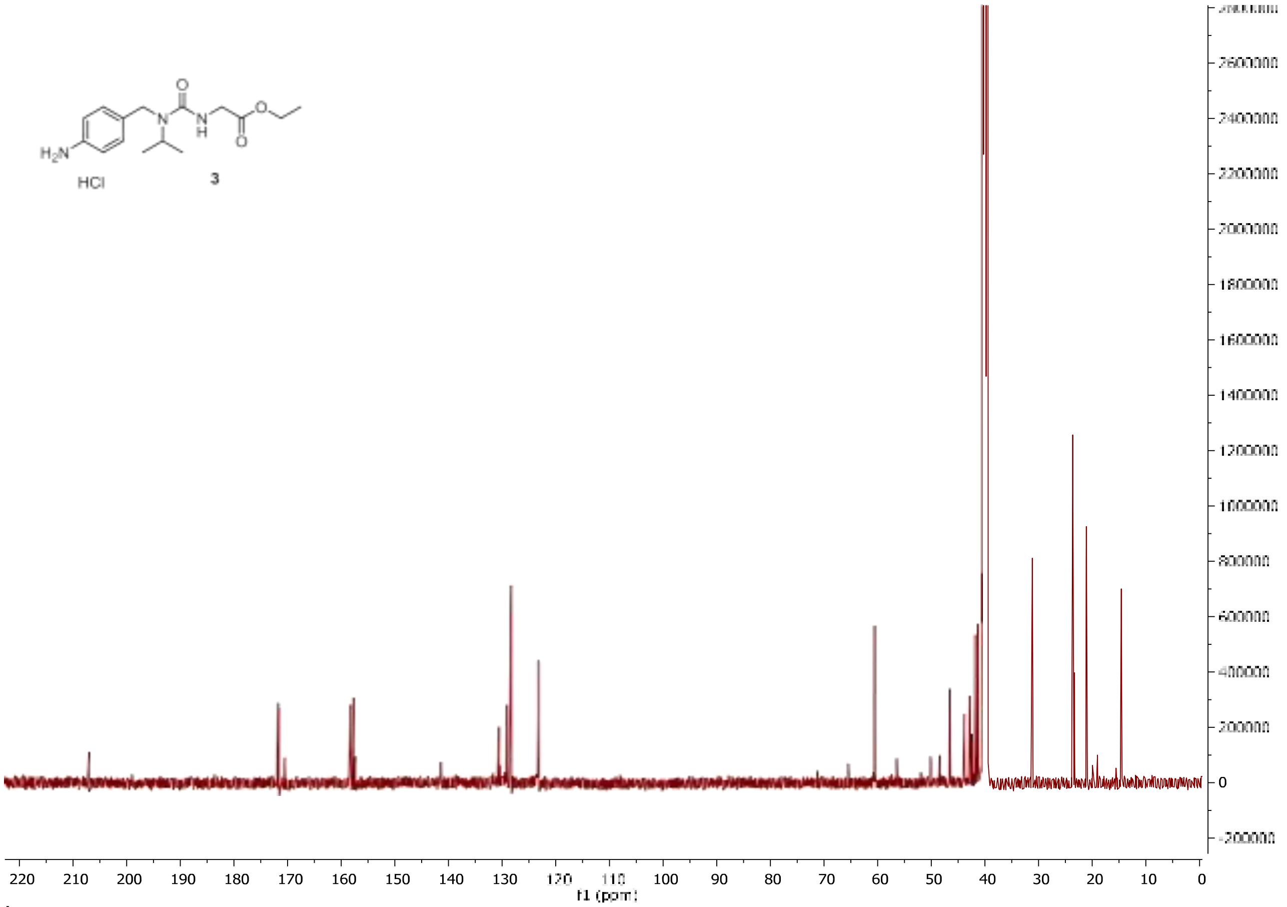

b

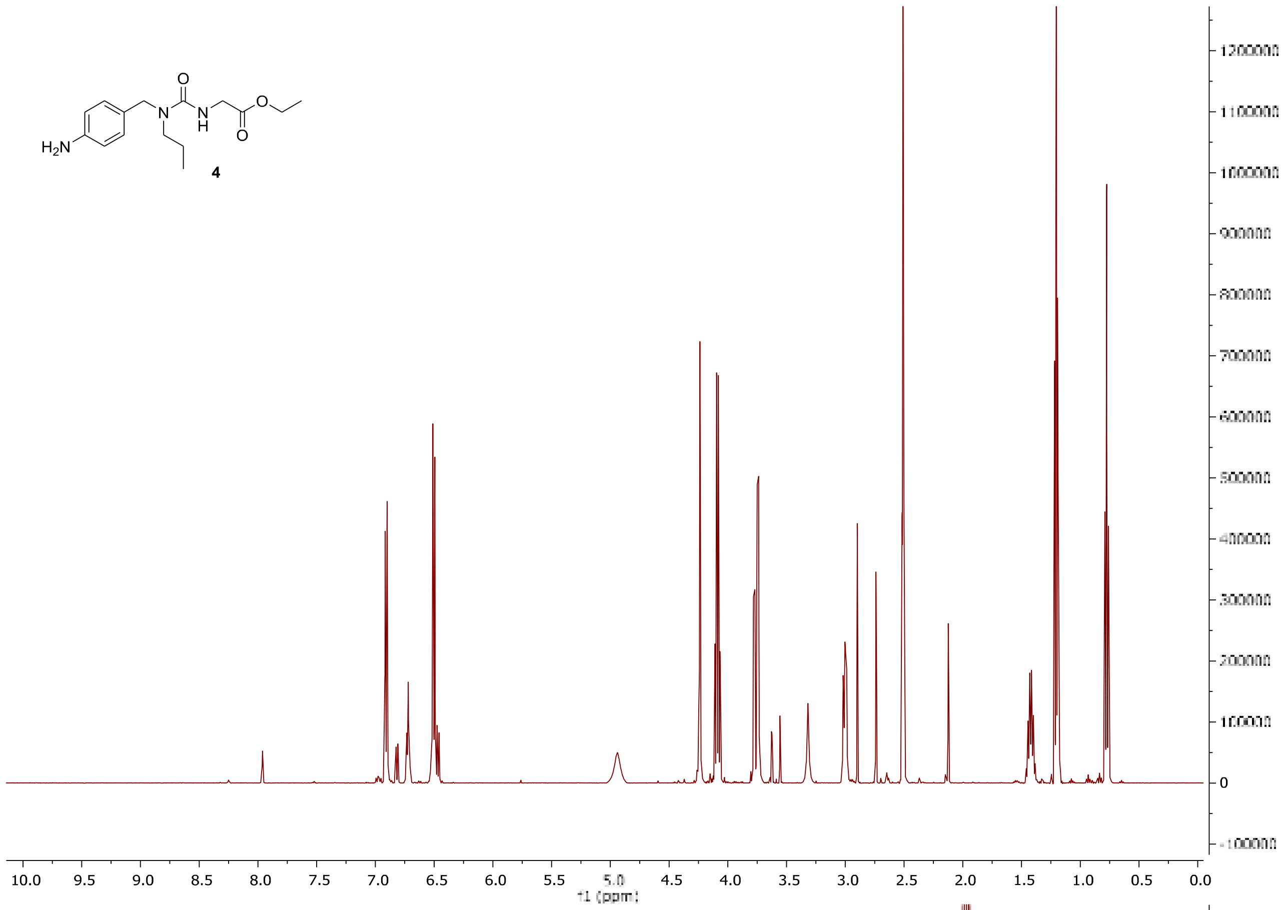

H2N
N
N
H
O
O
O
4
f1 (ppm)

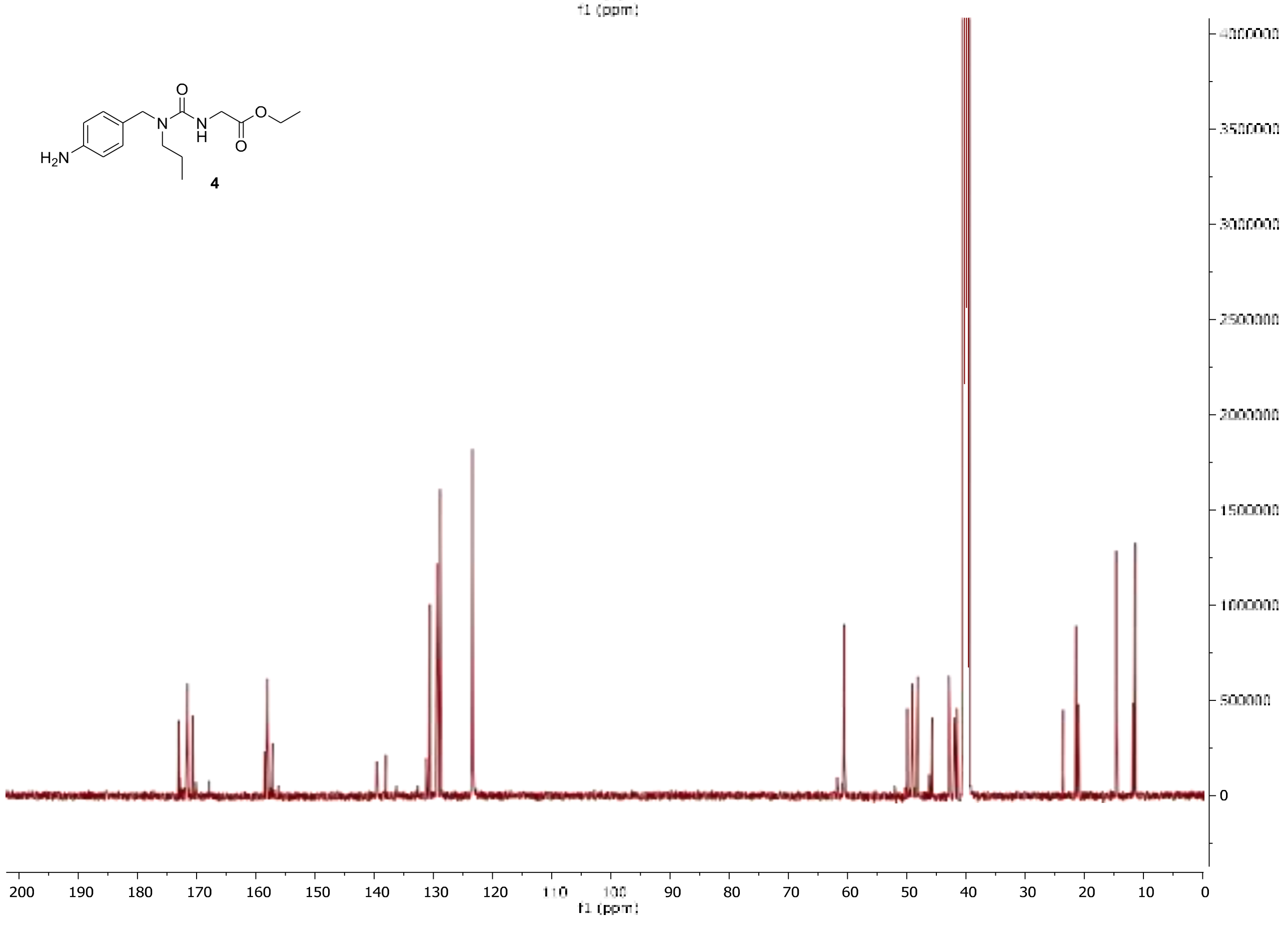

H2N
N
N
H
O
O
O
4
f1 (ppm)

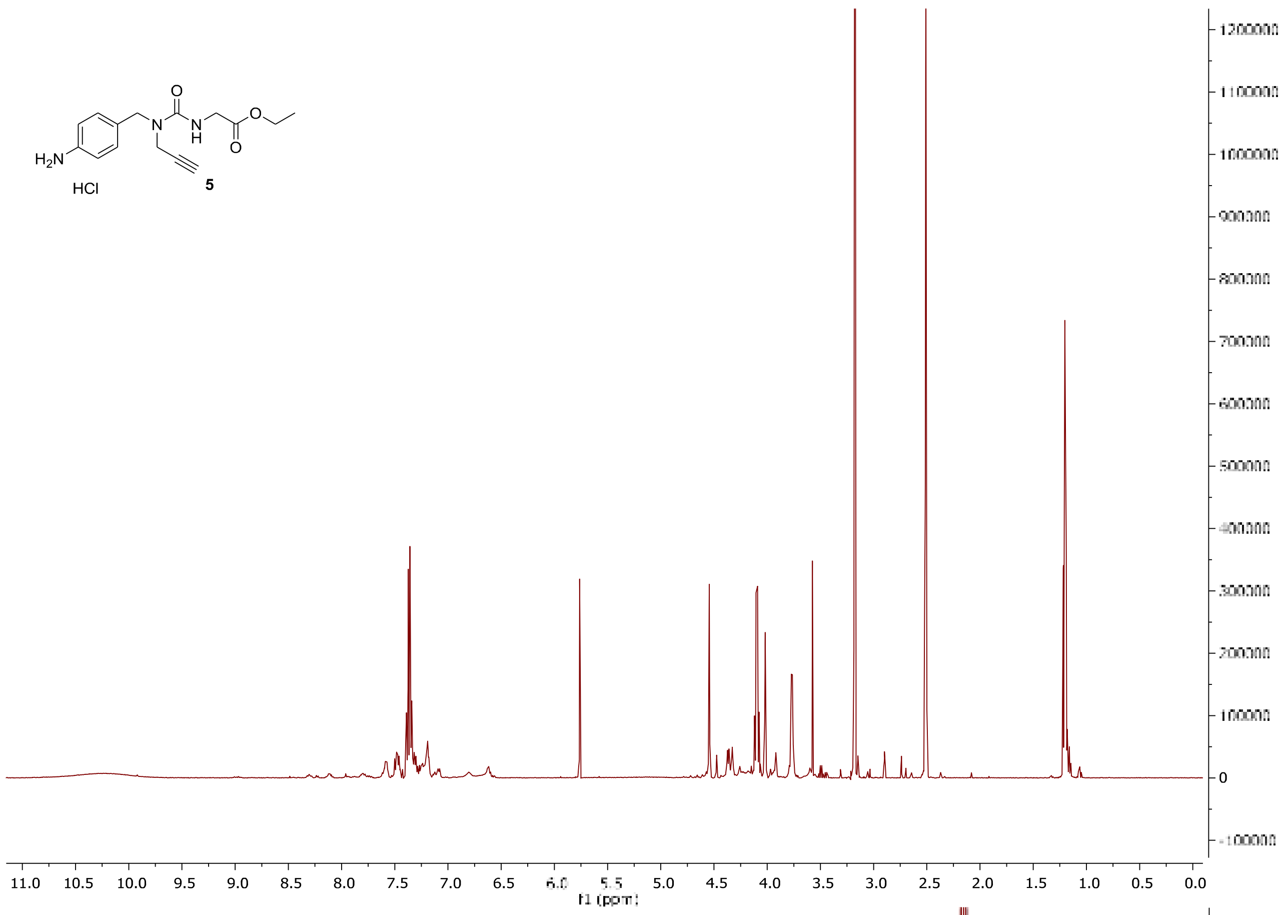
H2N
HCl
5
f1 (ppm)

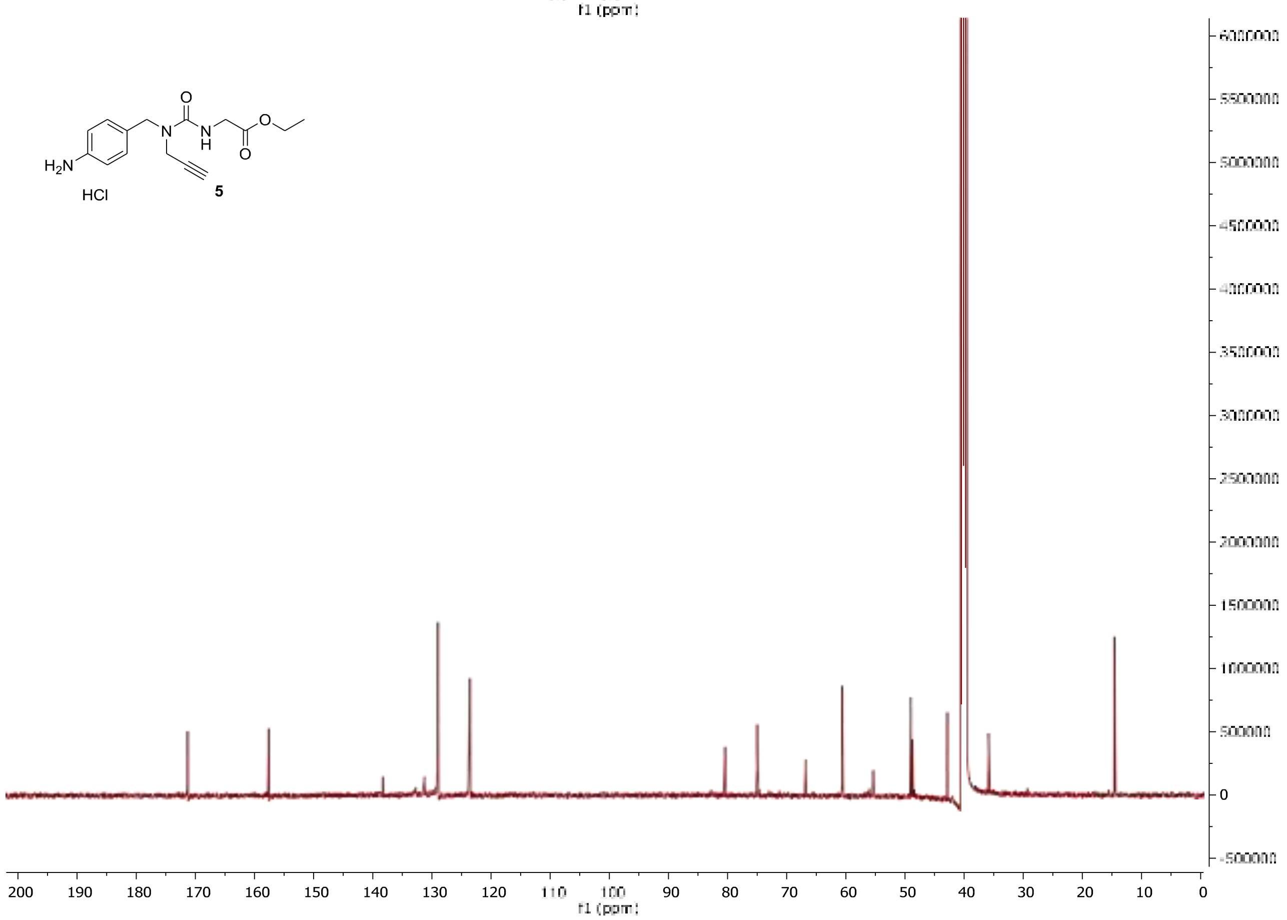
H2N
HCl
5
f1 (ppm)

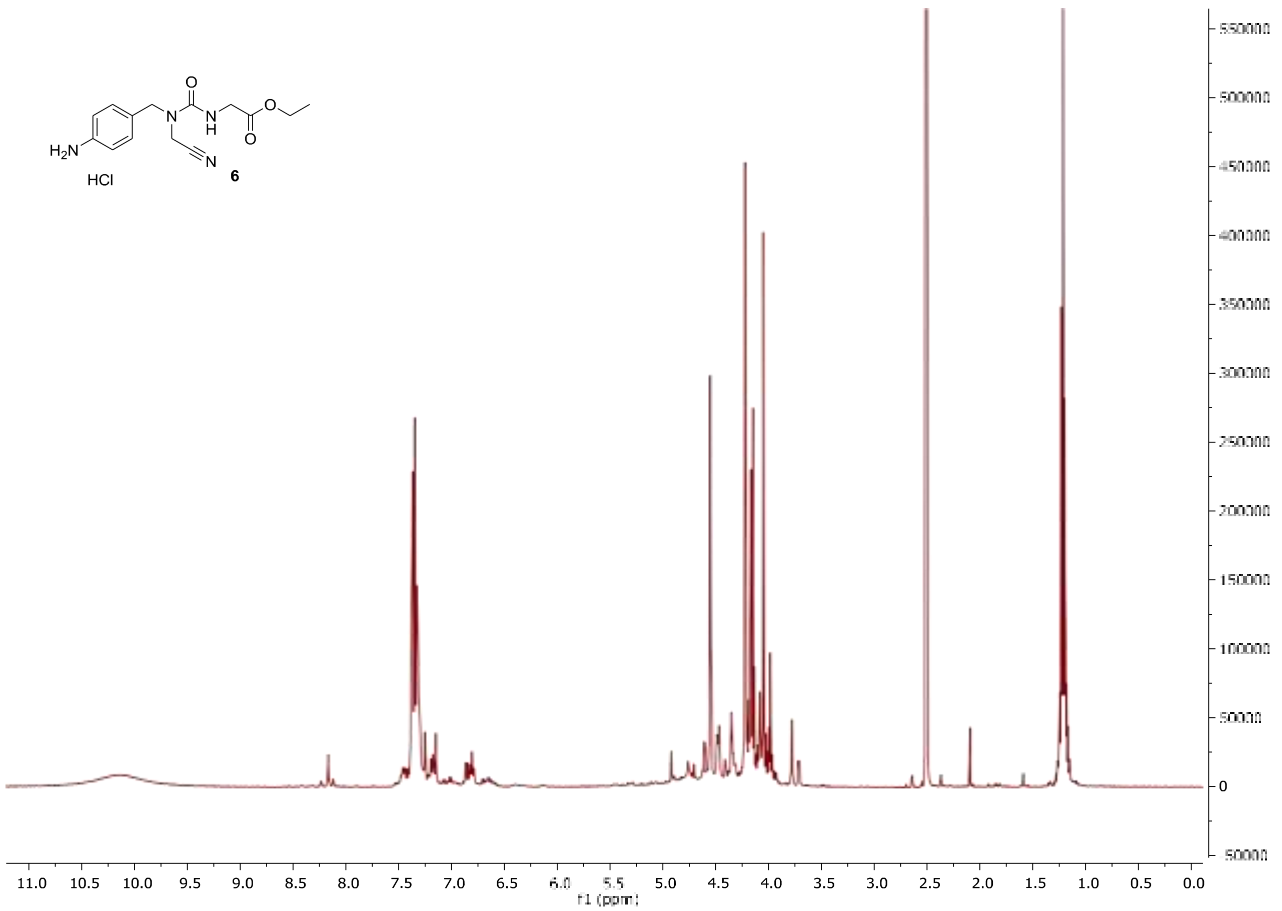
H2N
HCl
6
f1 (ppm)

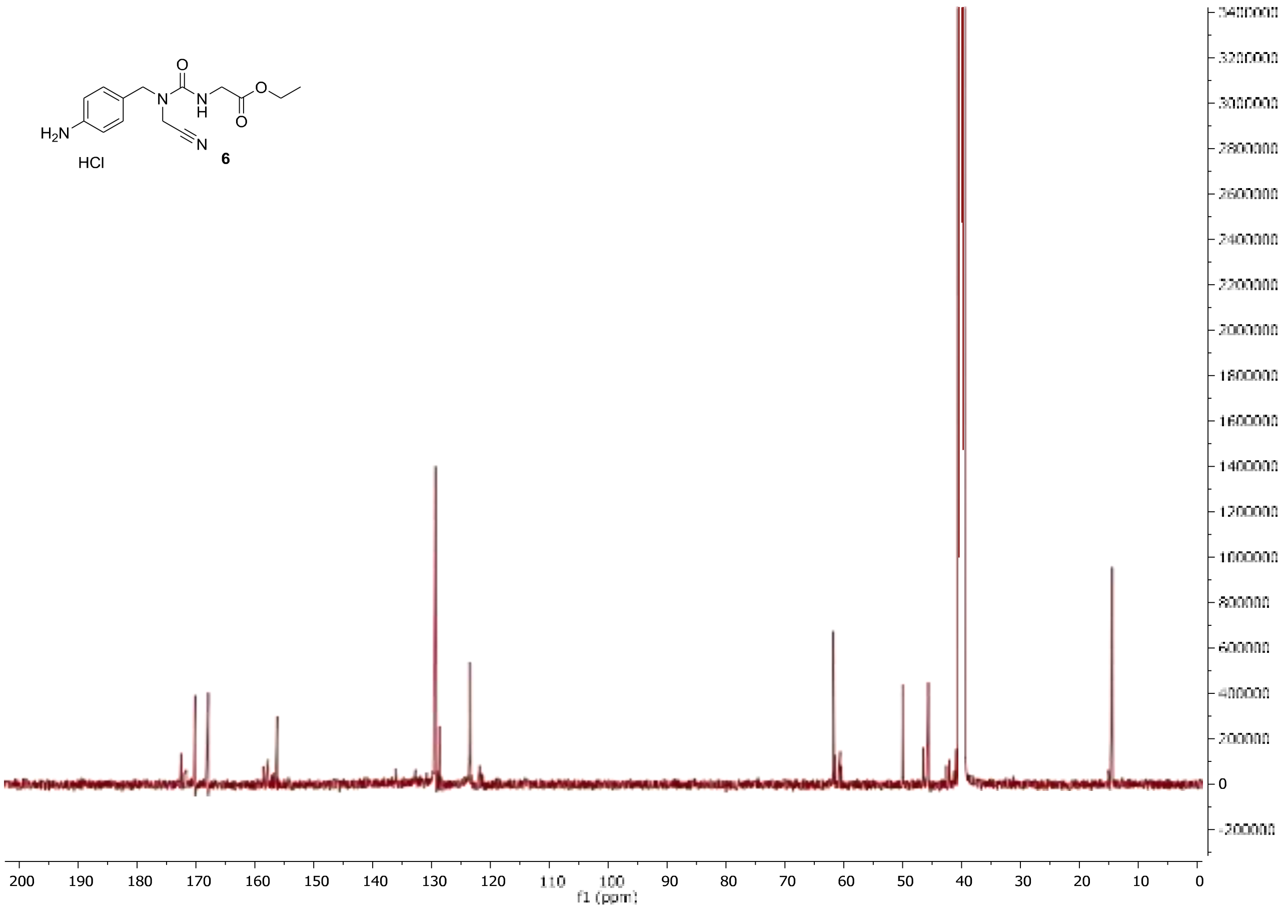
H2N
HCl
6
f1 (ppm)

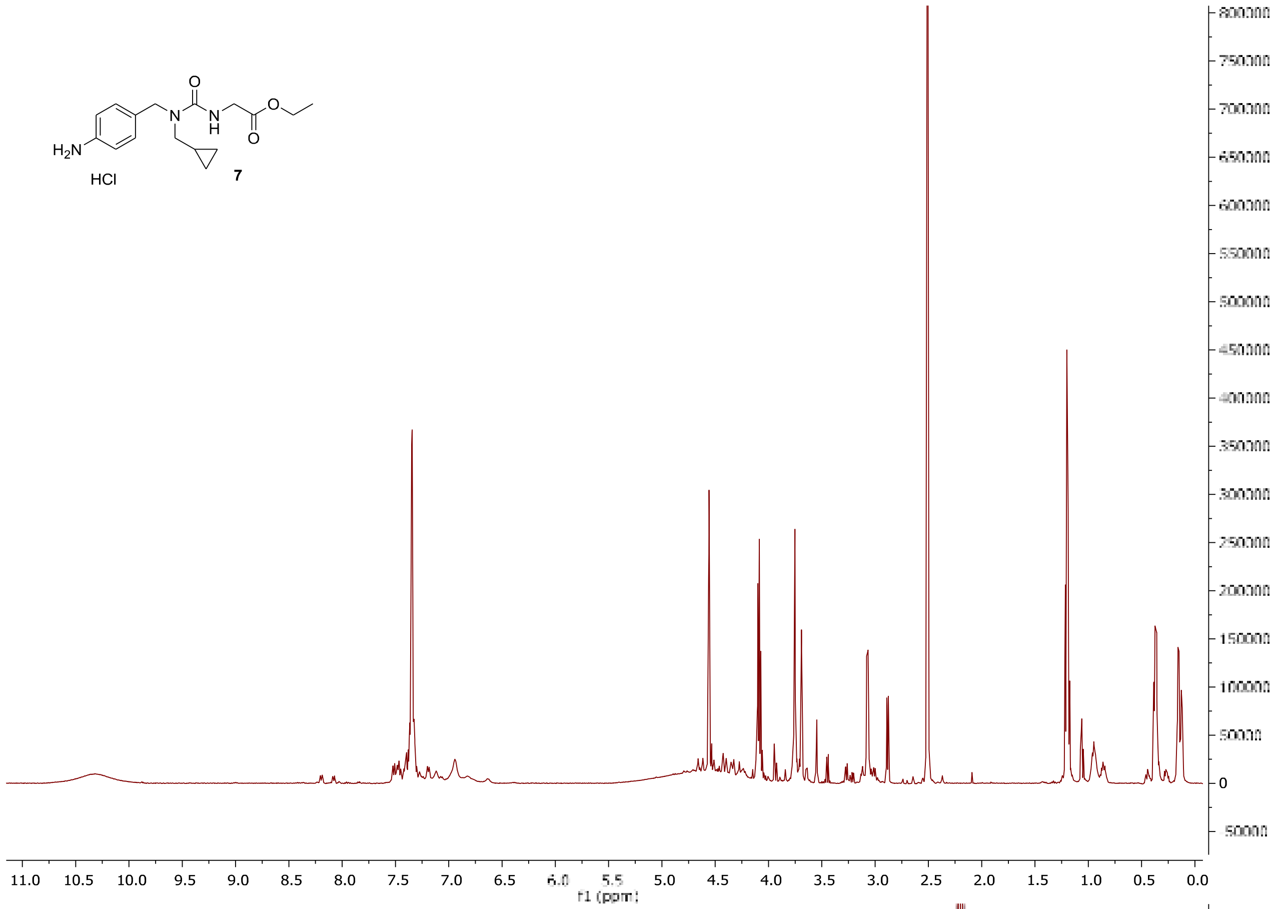
H2N
HCl
7
f1 (ppm)

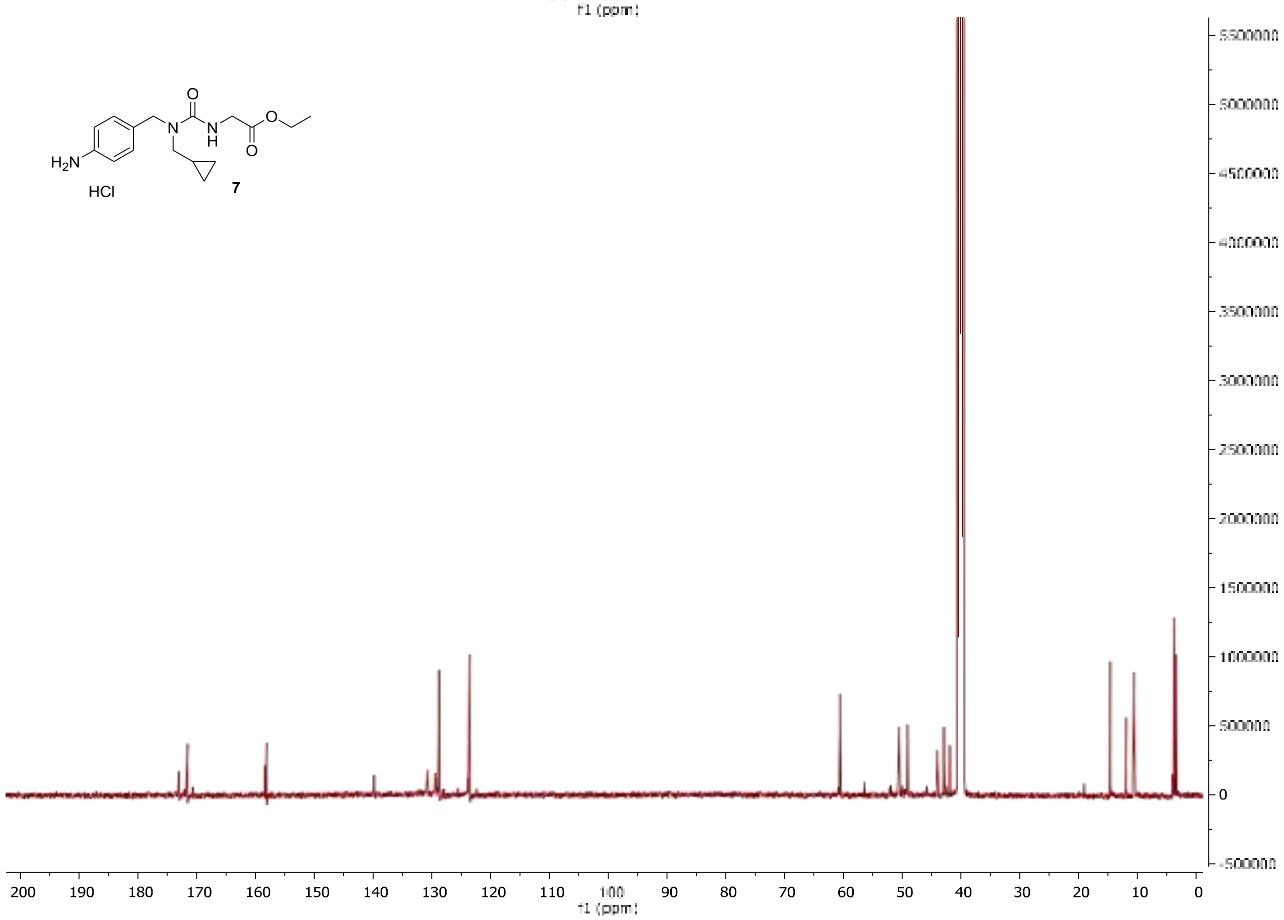
H2N
HCl
7
f1 (ppm)

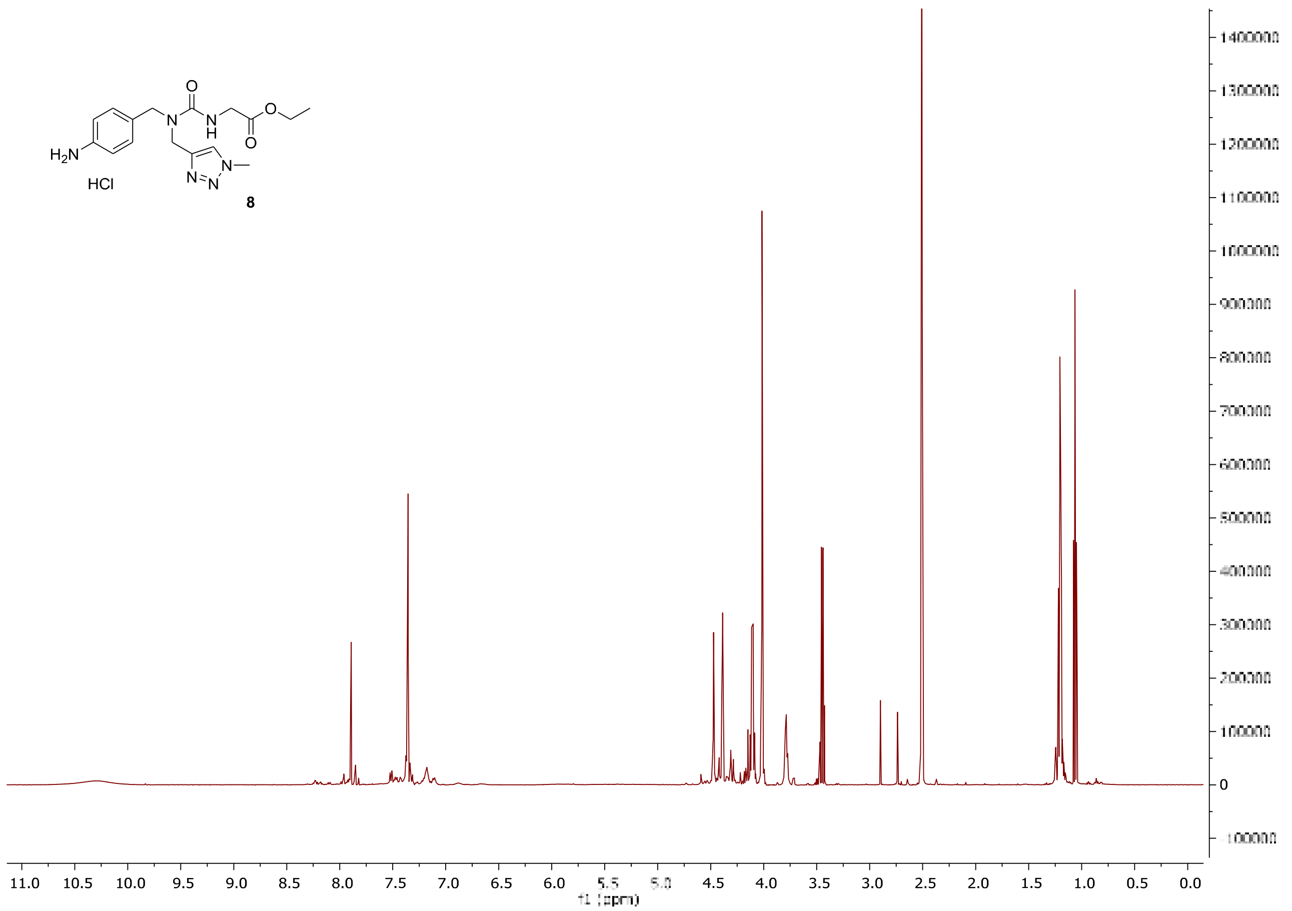
H2N
HCl
8
11.0
10.5
10.0
9.5
9.0
8.5
8.0
7.5
7.0
6.5
6.0
5.5
5.0
4.5
4.0
3.5
3.0
2.5
2.0
1.5
1.0
0.5
0.0
f1 (ppm)

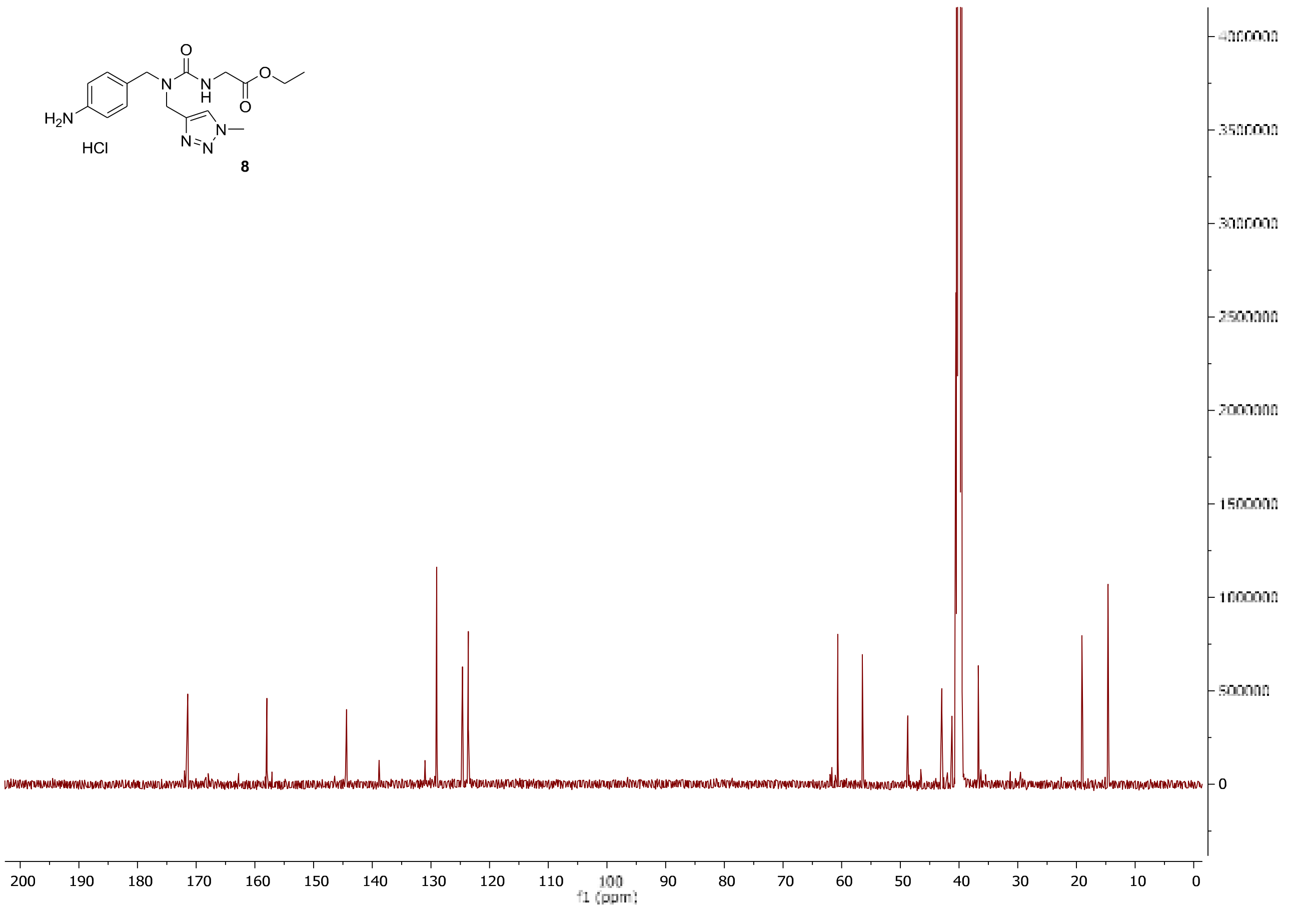
H2N
HCl
8
200
190
180
170
160
150
140
130
120
110
100
90
80
70
60
50
40
30
20
10
0
f1 (ppm)

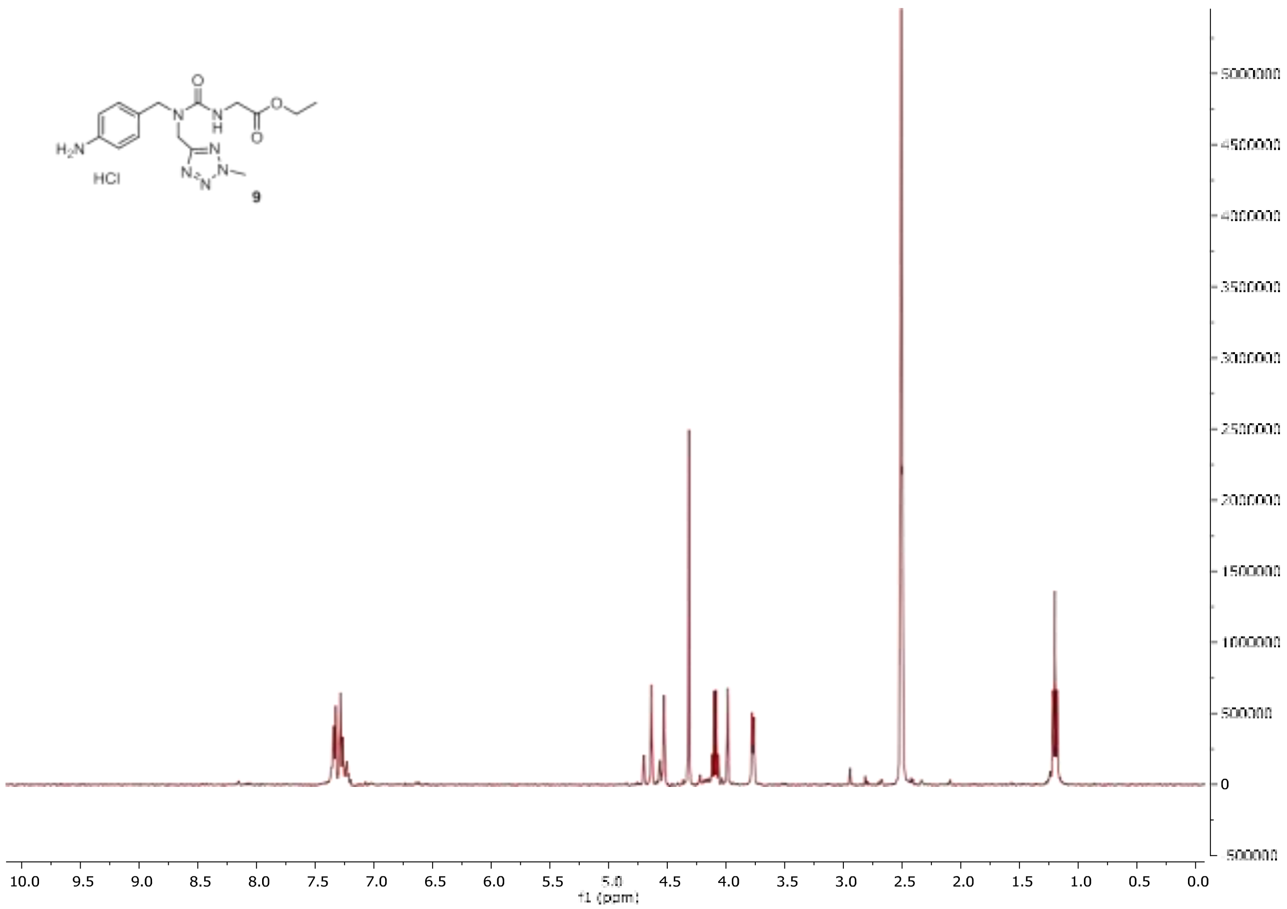
H2N
HCl
9
f1 (ppm)

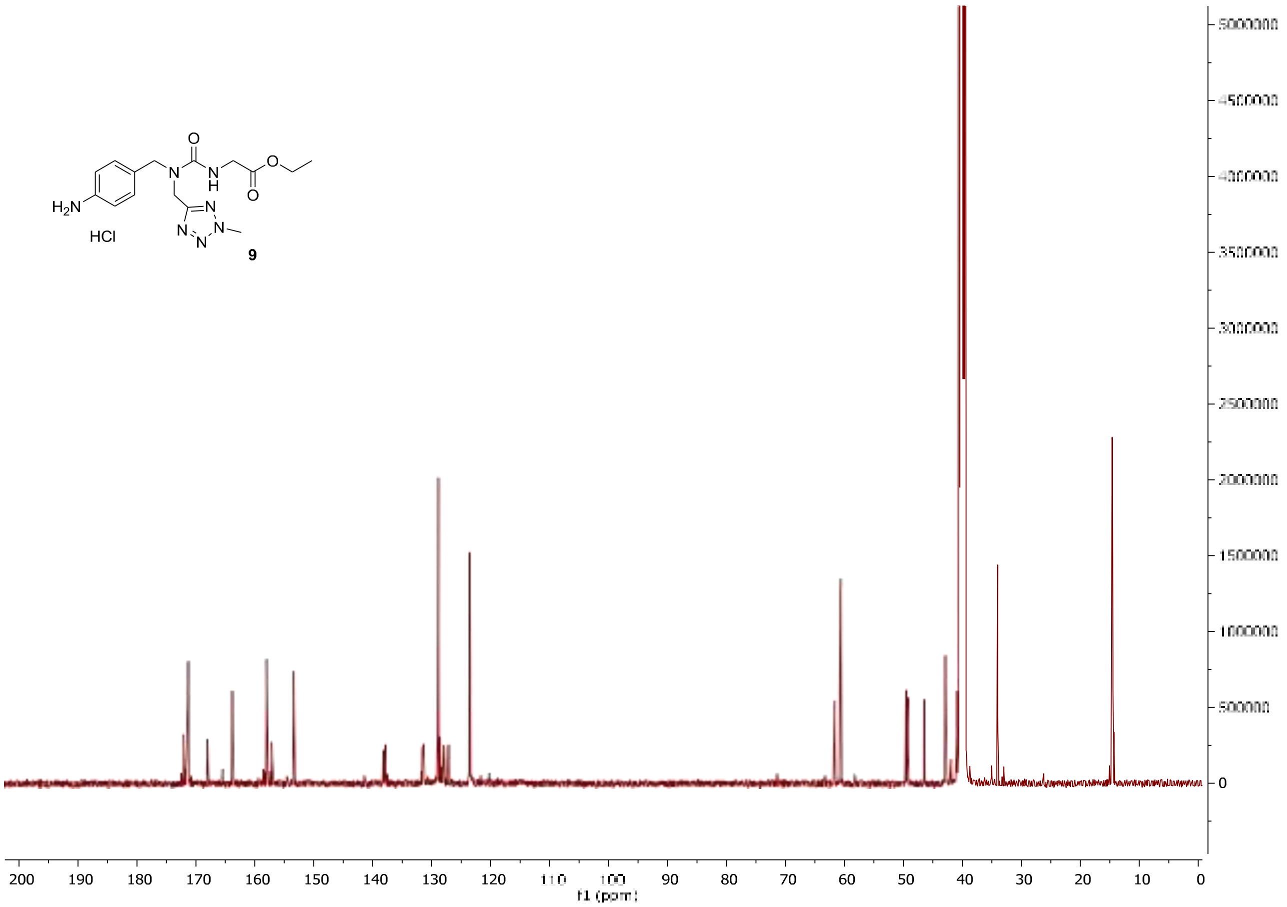
H2N
HCl
9
f1 (ppm)

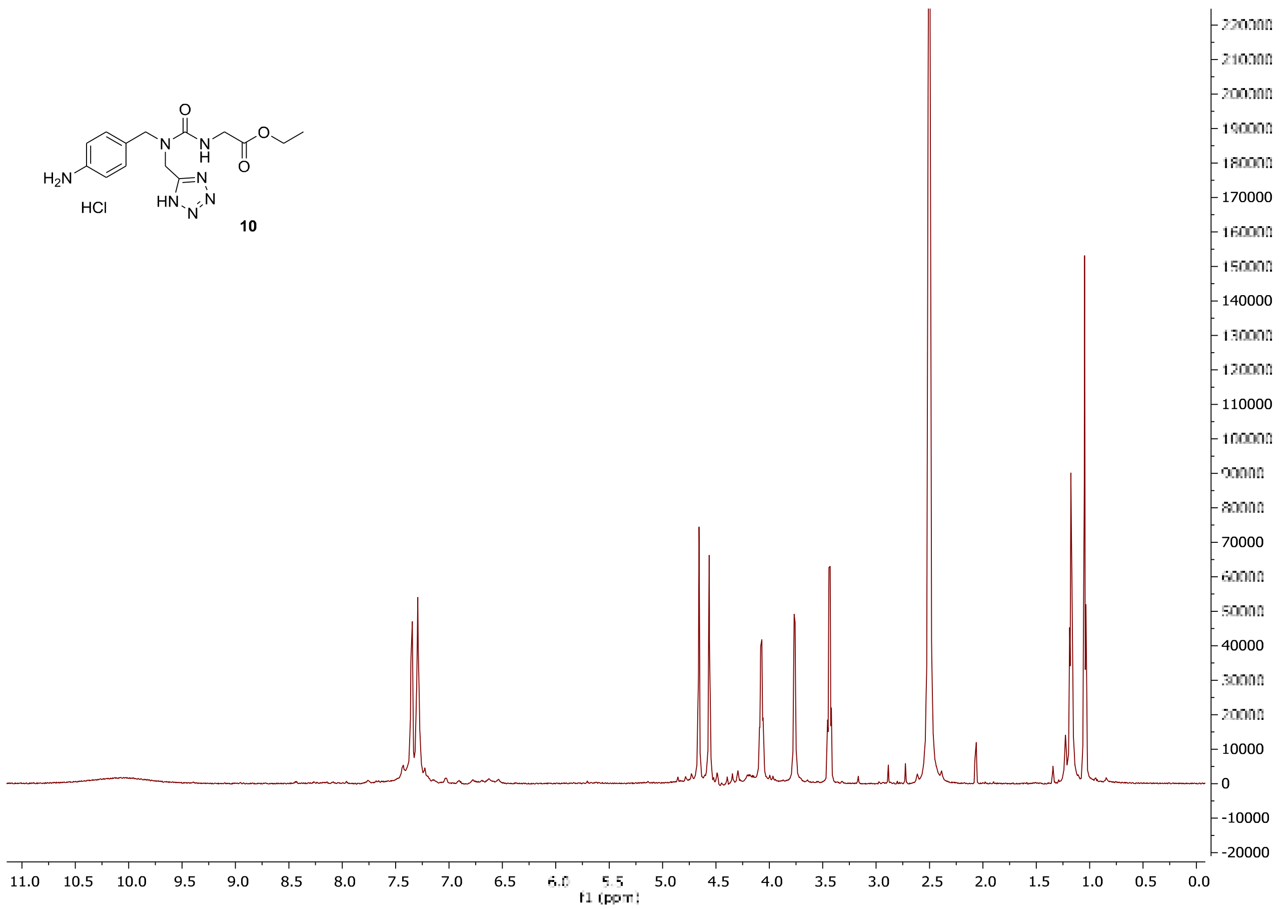
H2N
HCl
O
N
N
H
O
O
HN
N
N
N
10
11.0 10.5 10.0 9.5 9.0 8.5 8.0 7.5 7.0 6.5 6.0 5.5 5.0 4.5 4.0 3.5 3.0 2.5 2.0 1.5 1.0 0.5 0.0
f1 (ppm)
220000
210000
200000
190000
180000
170000
160000
150000
140000
130000
120000
110000
100000
90000
80000
70000
60000
50000
40000
30000
20000
10000
0
-10000
-20000

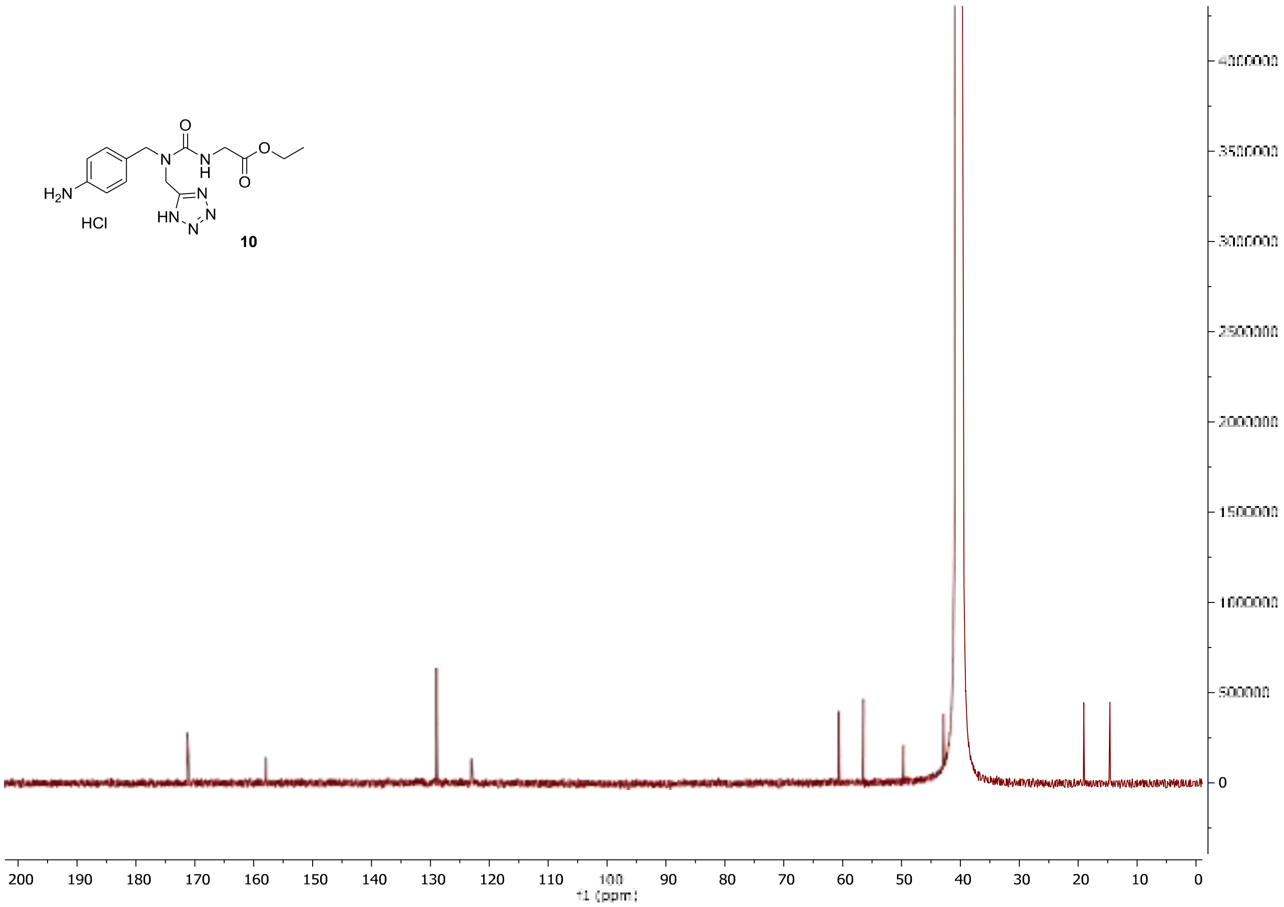
H2N
HCl
O
N
N
H
O
O
HN
N
N
N
10
200 190 180 170 160 150 140 130 120 110 100 90 80 70 60 50 40 30 20 10 0
f1 (ppm)
4000000
3500000
3000000
2500000
2000000
1500000
1000000
500000
0

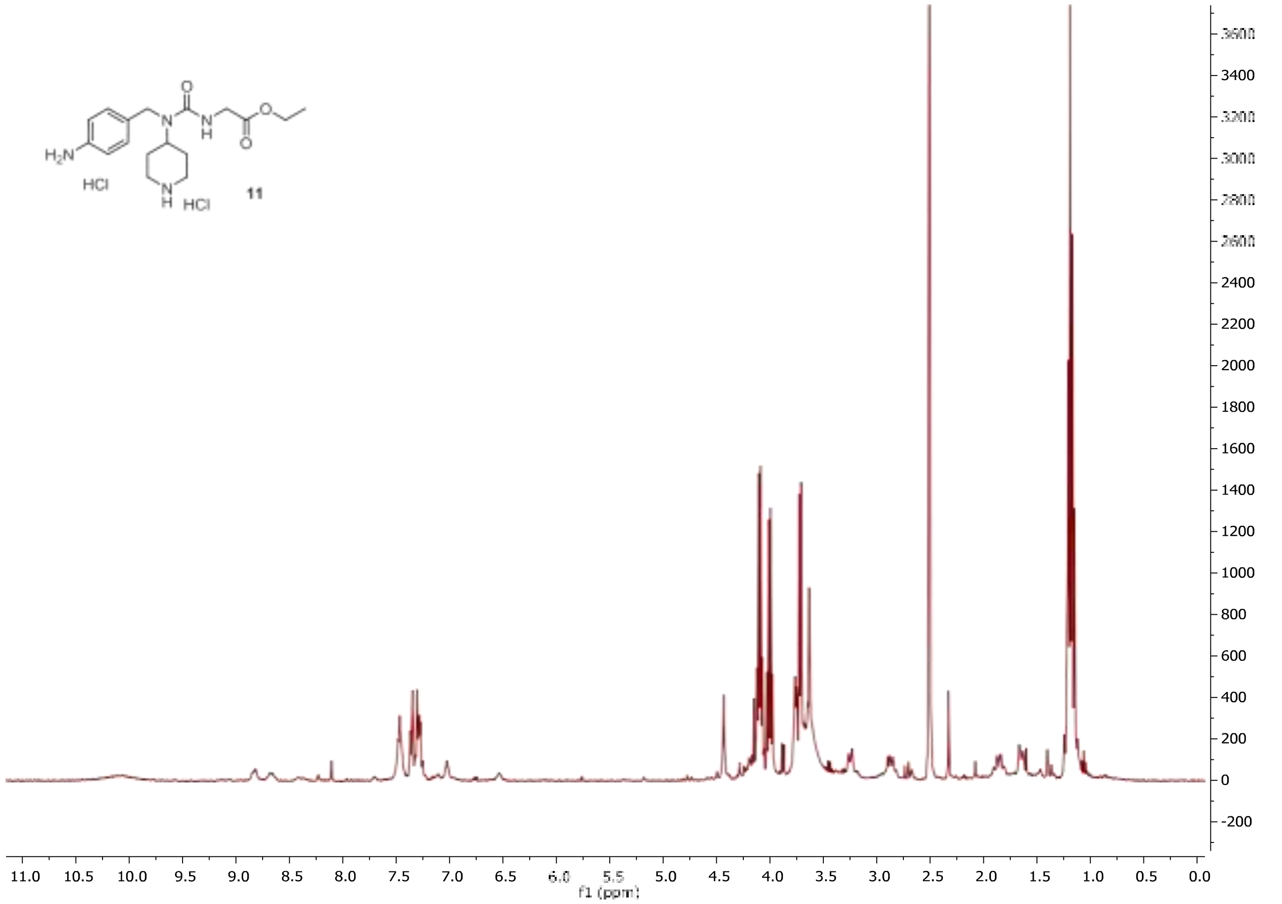
HCl
HCl
11
f1 (ppm)

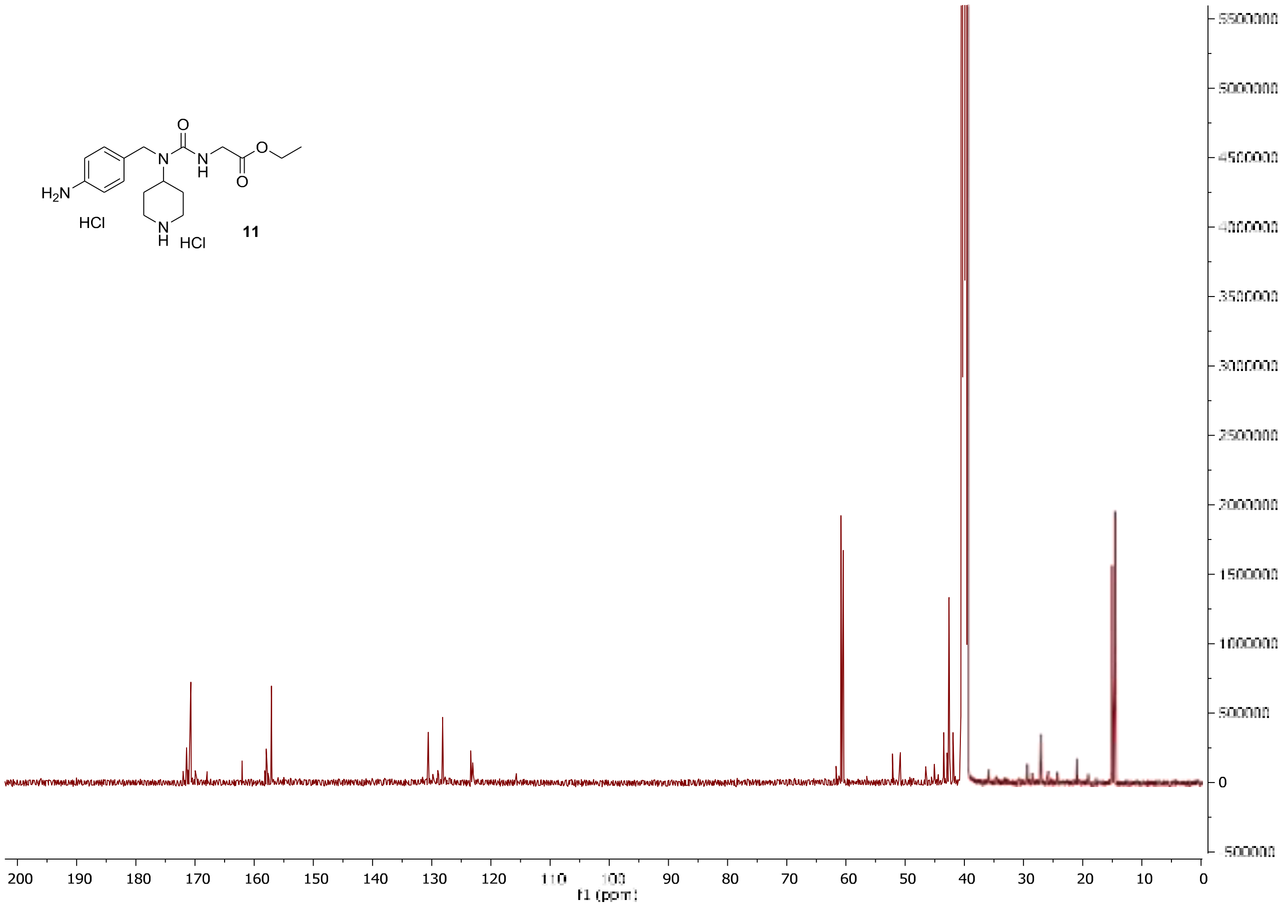
HCl
HCl
11
f1 (ppm)

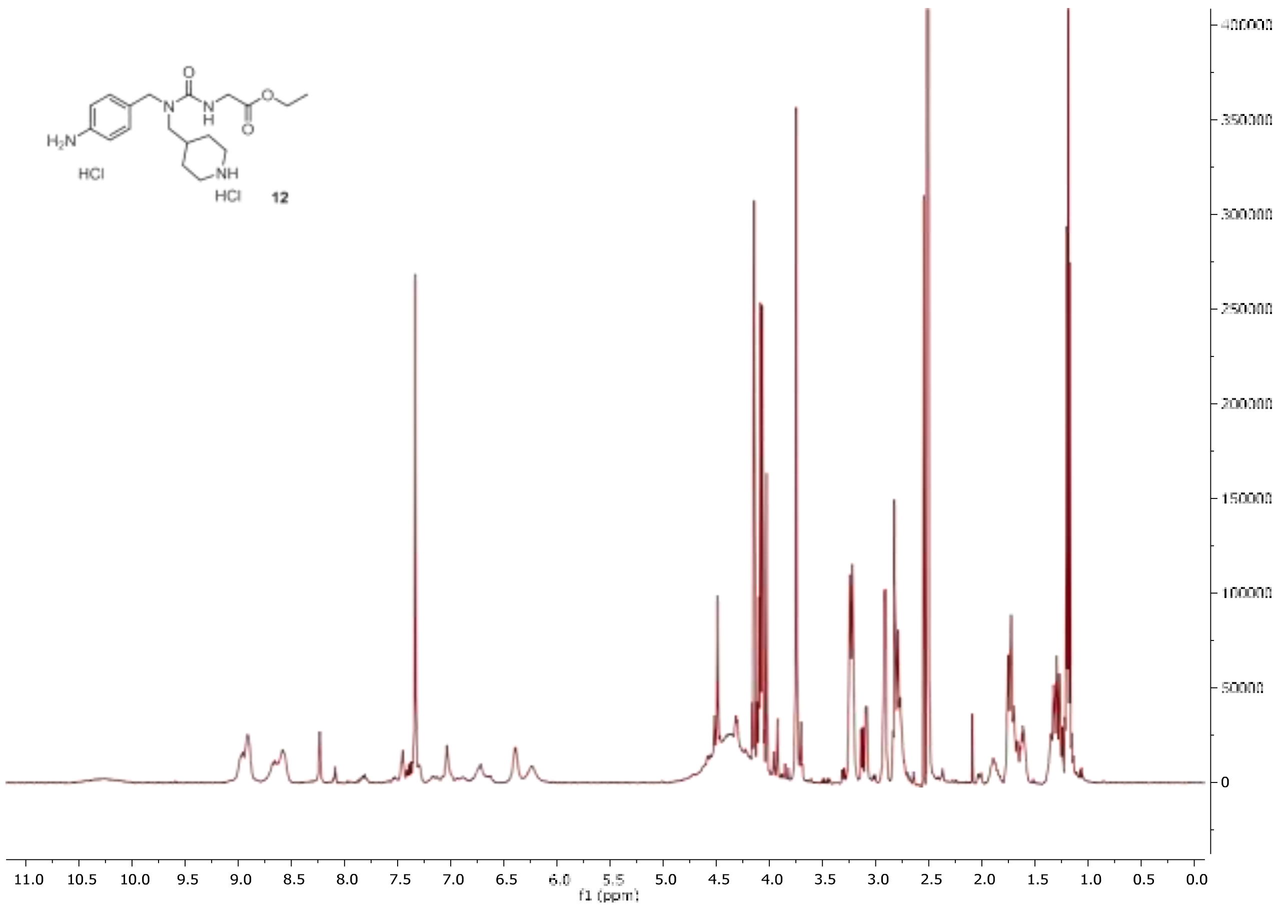
H2N
HCl
NH
HCl
12
f1 (ppm)

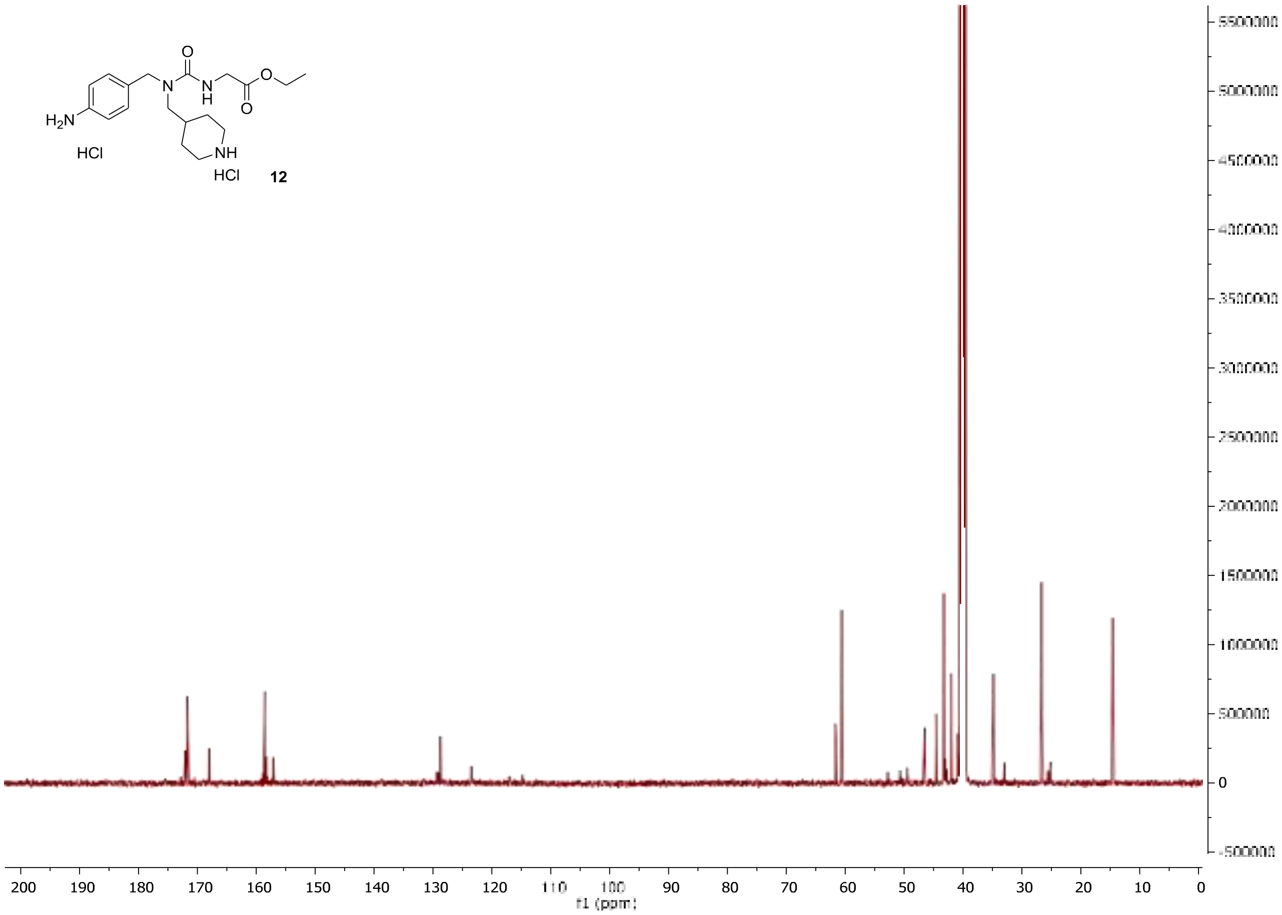
H2N
HCl
NH
HCl
12
f1 (ppm)

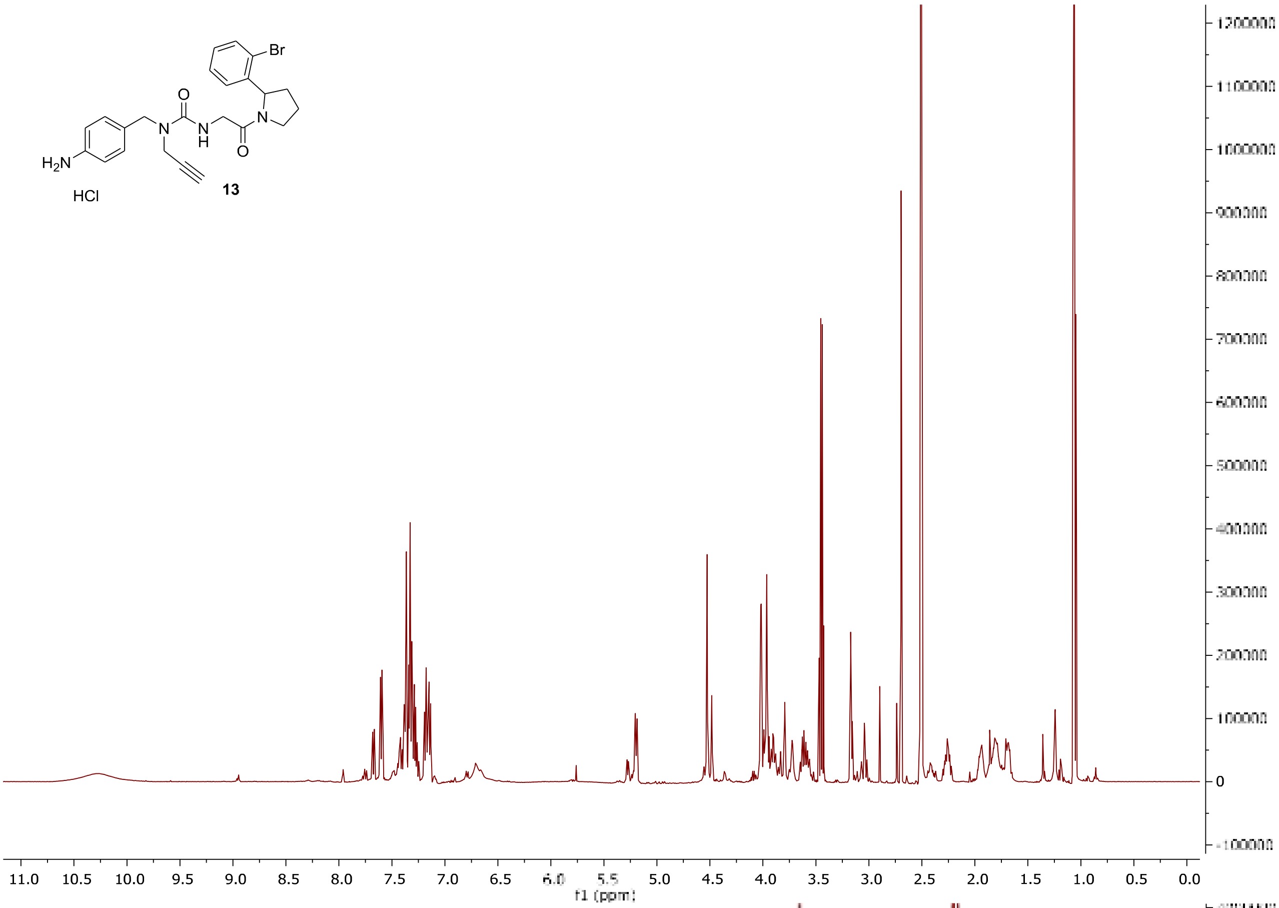
Br
O
N
H
N
O
H2N
HCl
13
f1 (ppm)

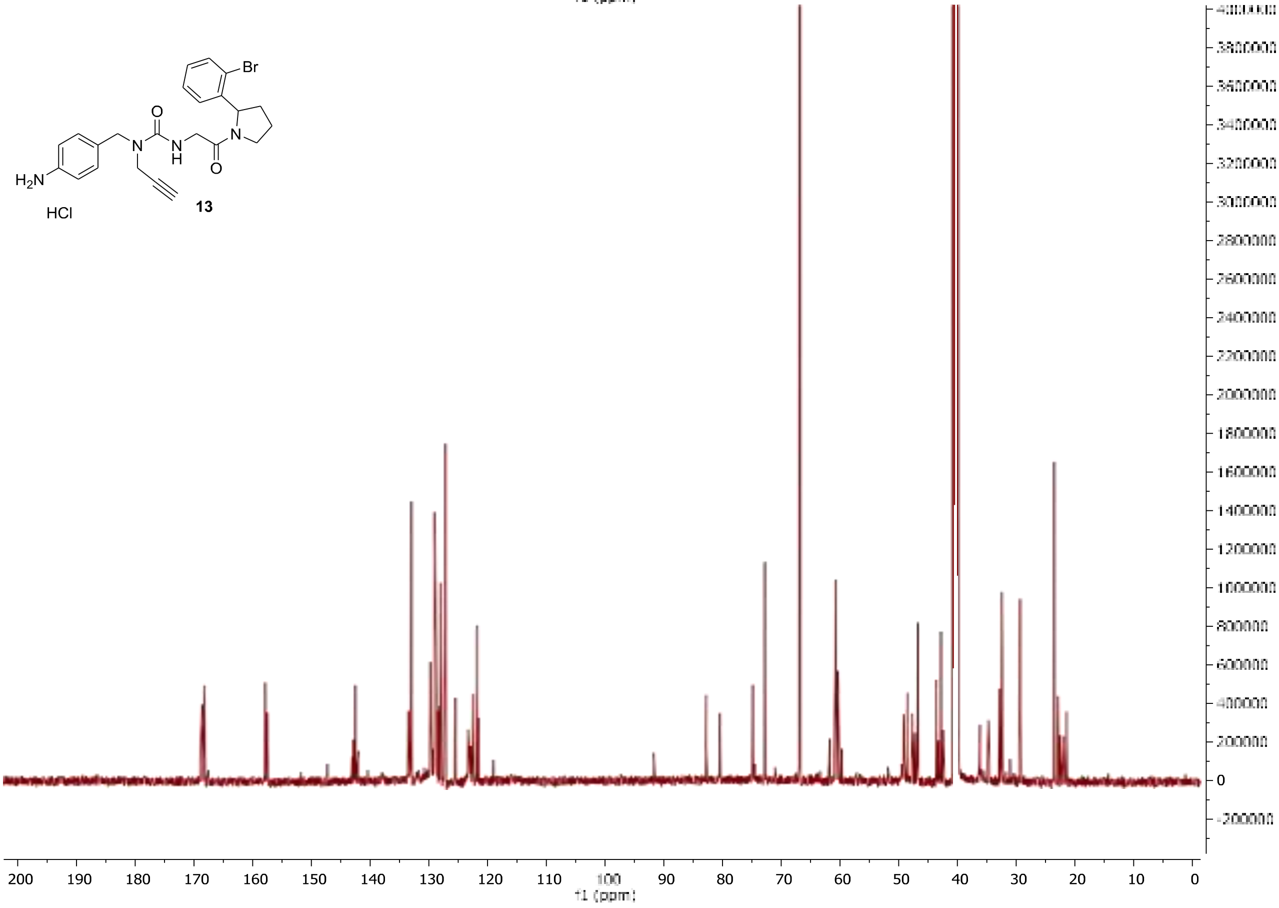
Br
O
N
H
N
O
H2N
HCl
13
f1 (ppm)

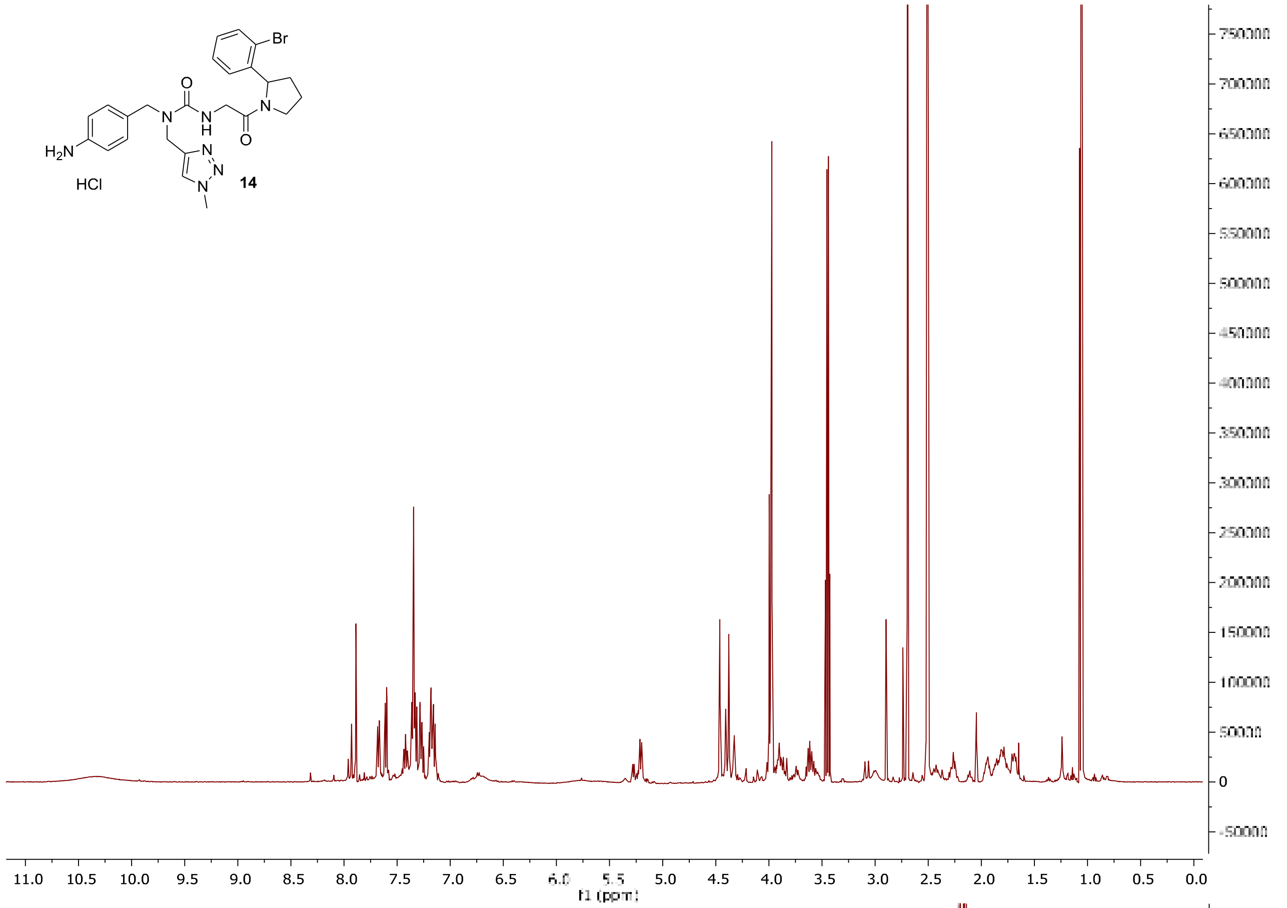
Br
O
N
H
N
H2N
N
N
N
HCl
14
f1 (ppm)

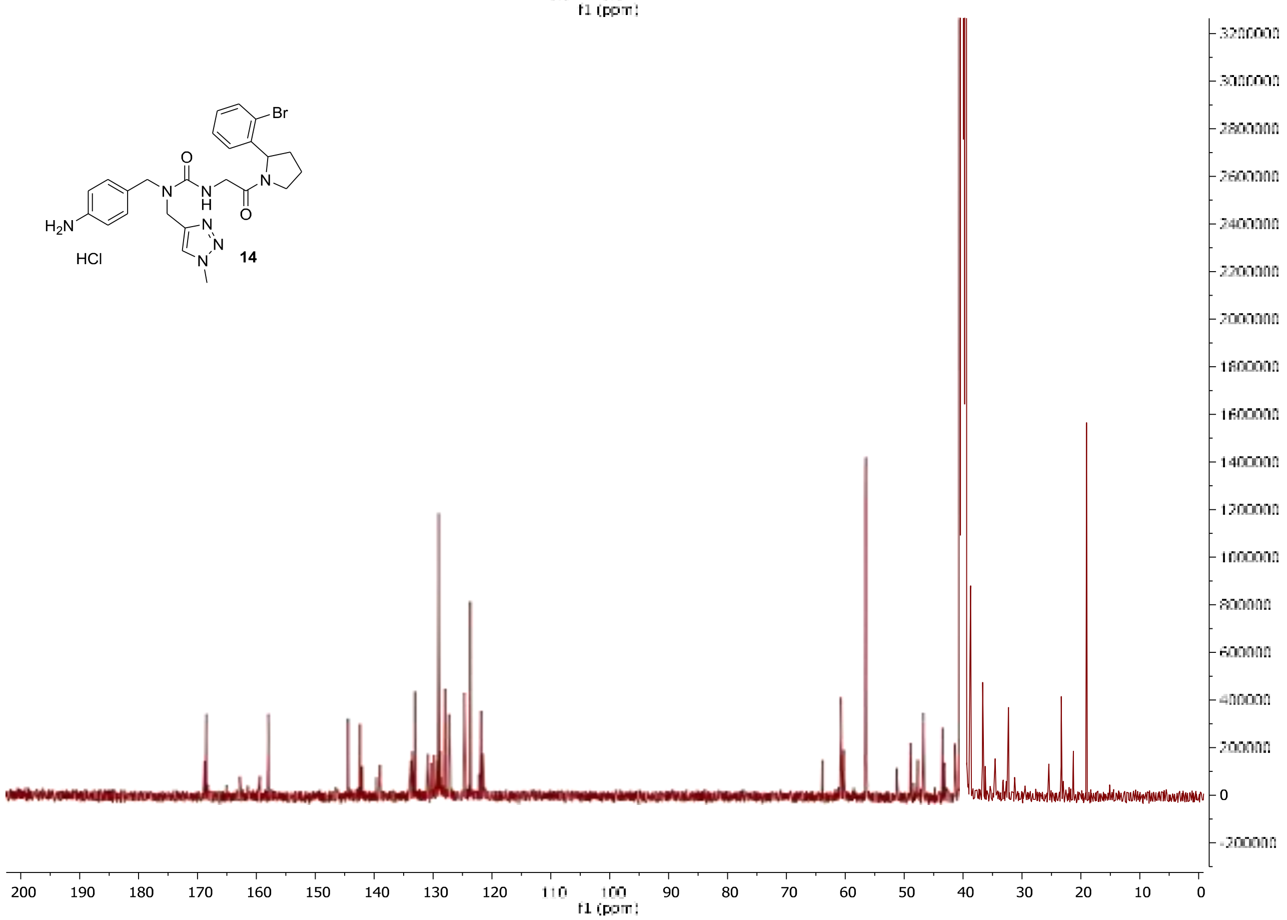
Br
O
N
H
N
H2N
N
N
N
HCl
14
f1 (ppm)

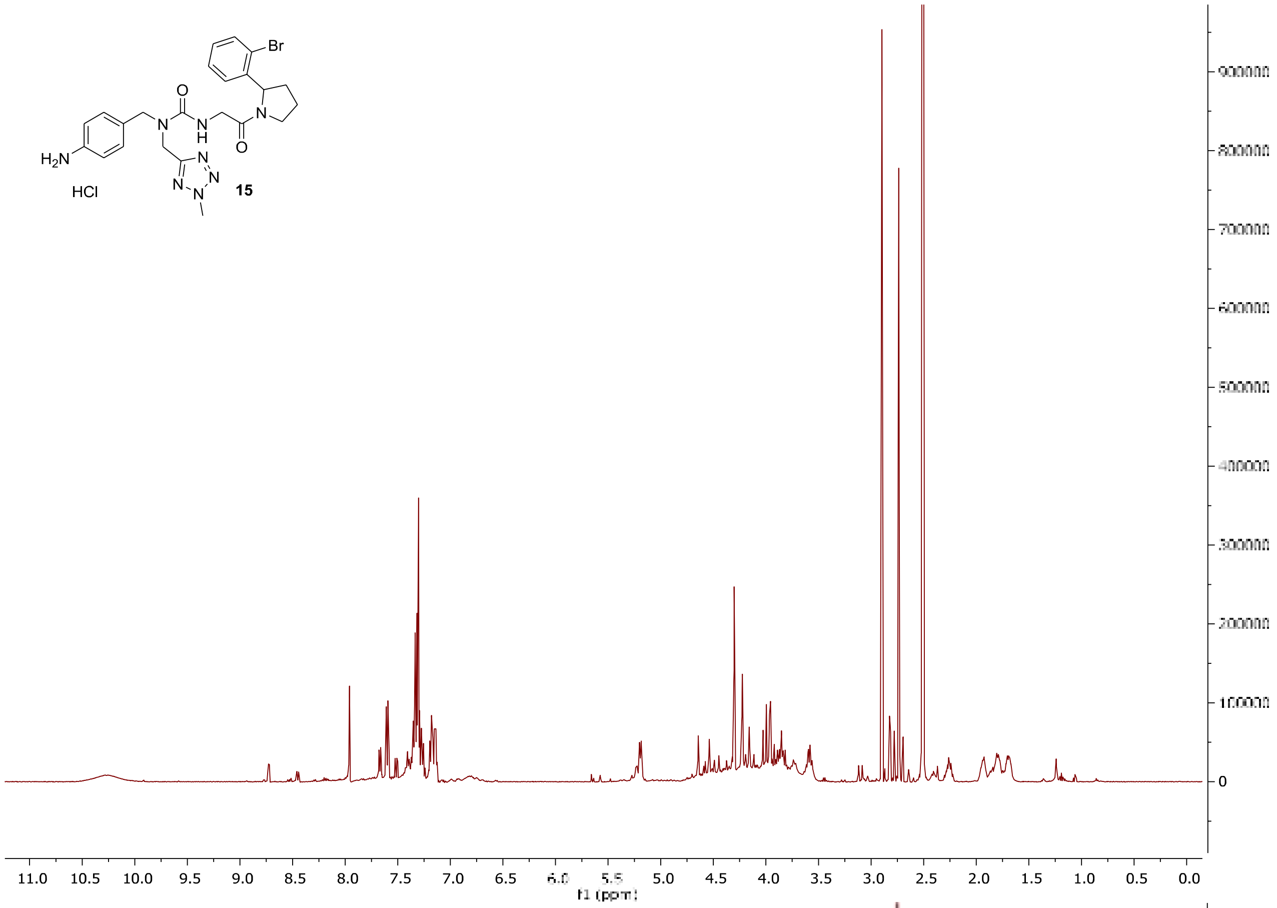
Br
O
N
N
H
N
O
H2N
N
N
N
N
HCl
15
f1 (ppm)

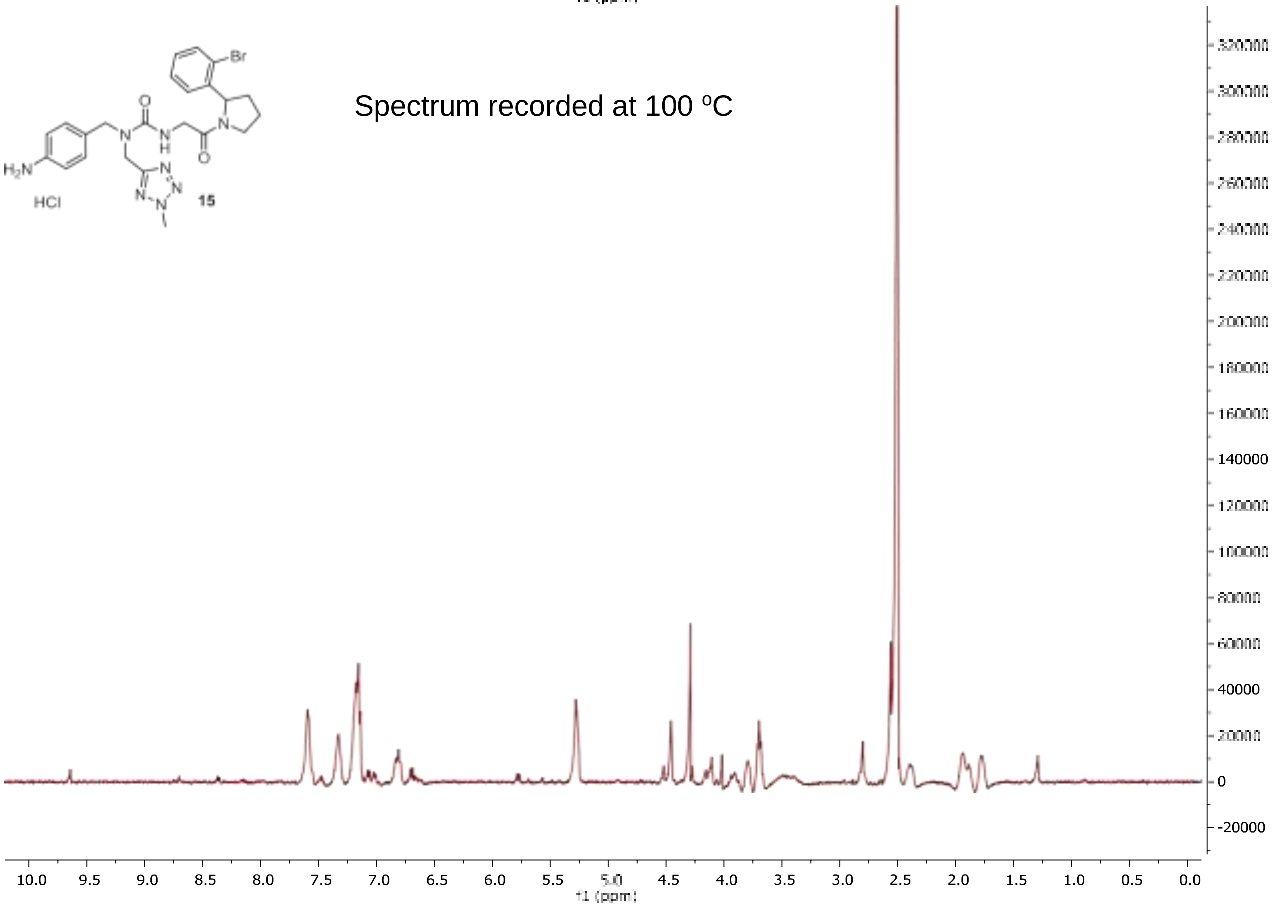
Br
H2N
HCl
15
Spectrum recorded at 100 ºC
f1 (ppm)

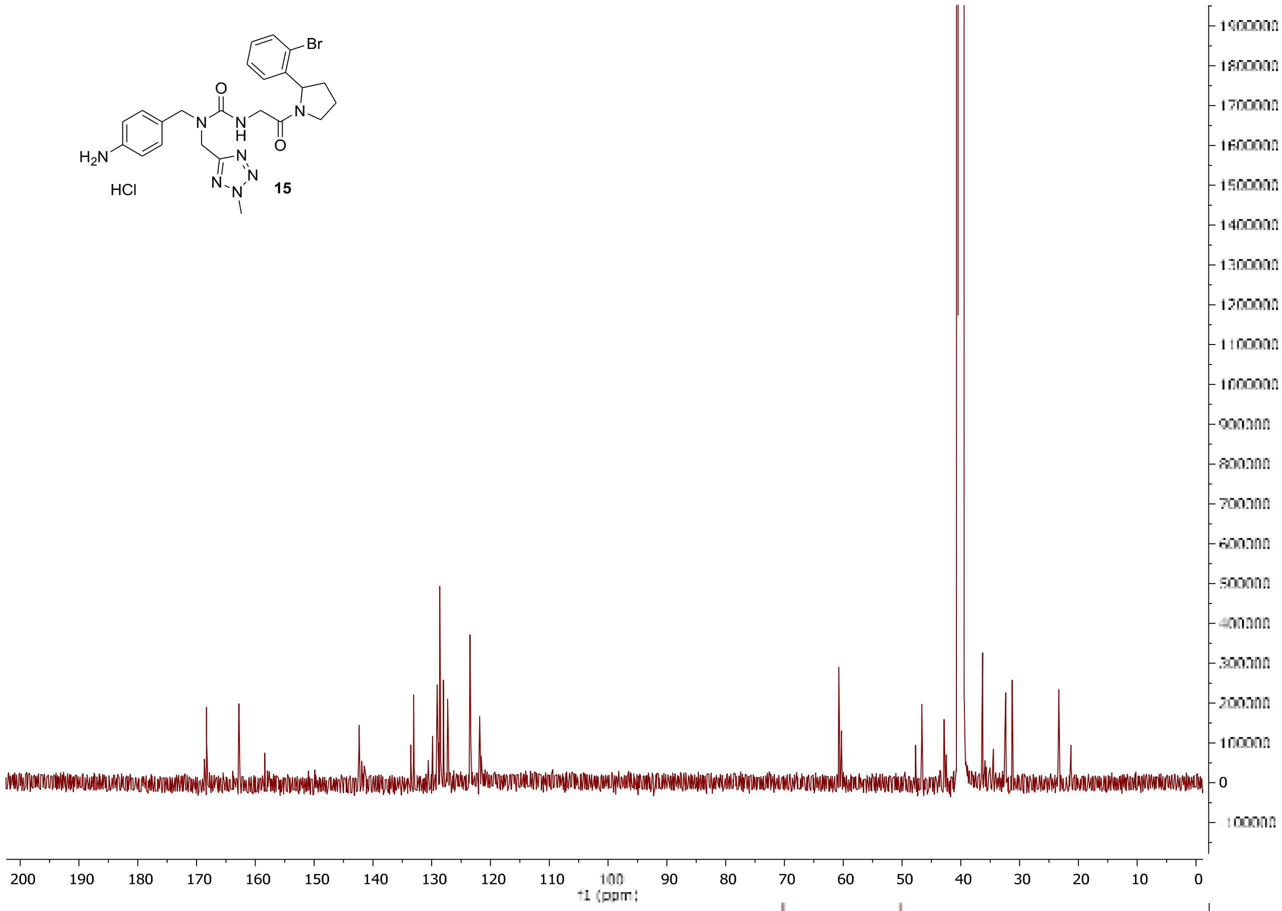

Br
O
N
N
H
N
O
H_2N
N
N
N
N
HCl
15

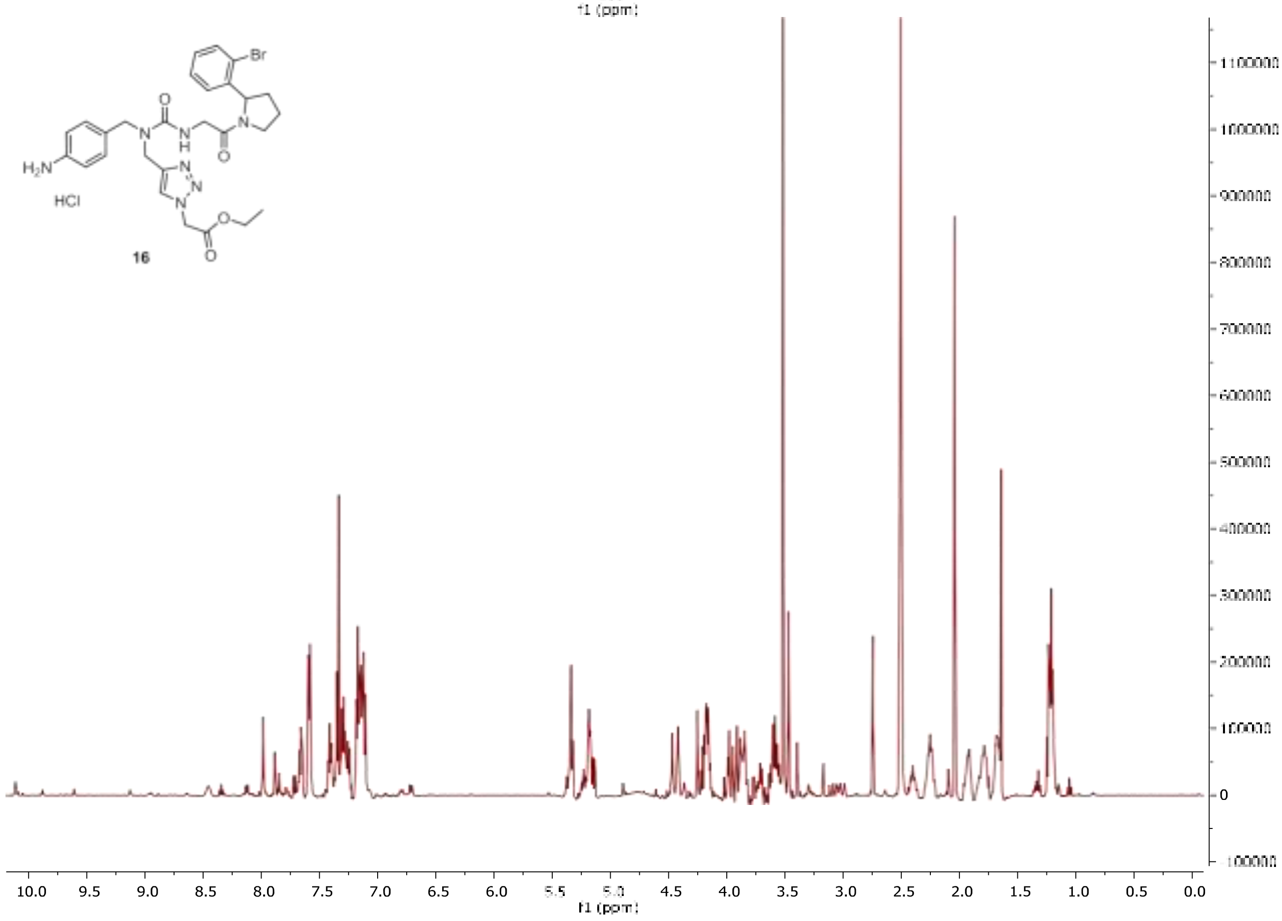

Br
O
N
N
H
N
O
H_2N
N
N
N
HCl
O
O
16

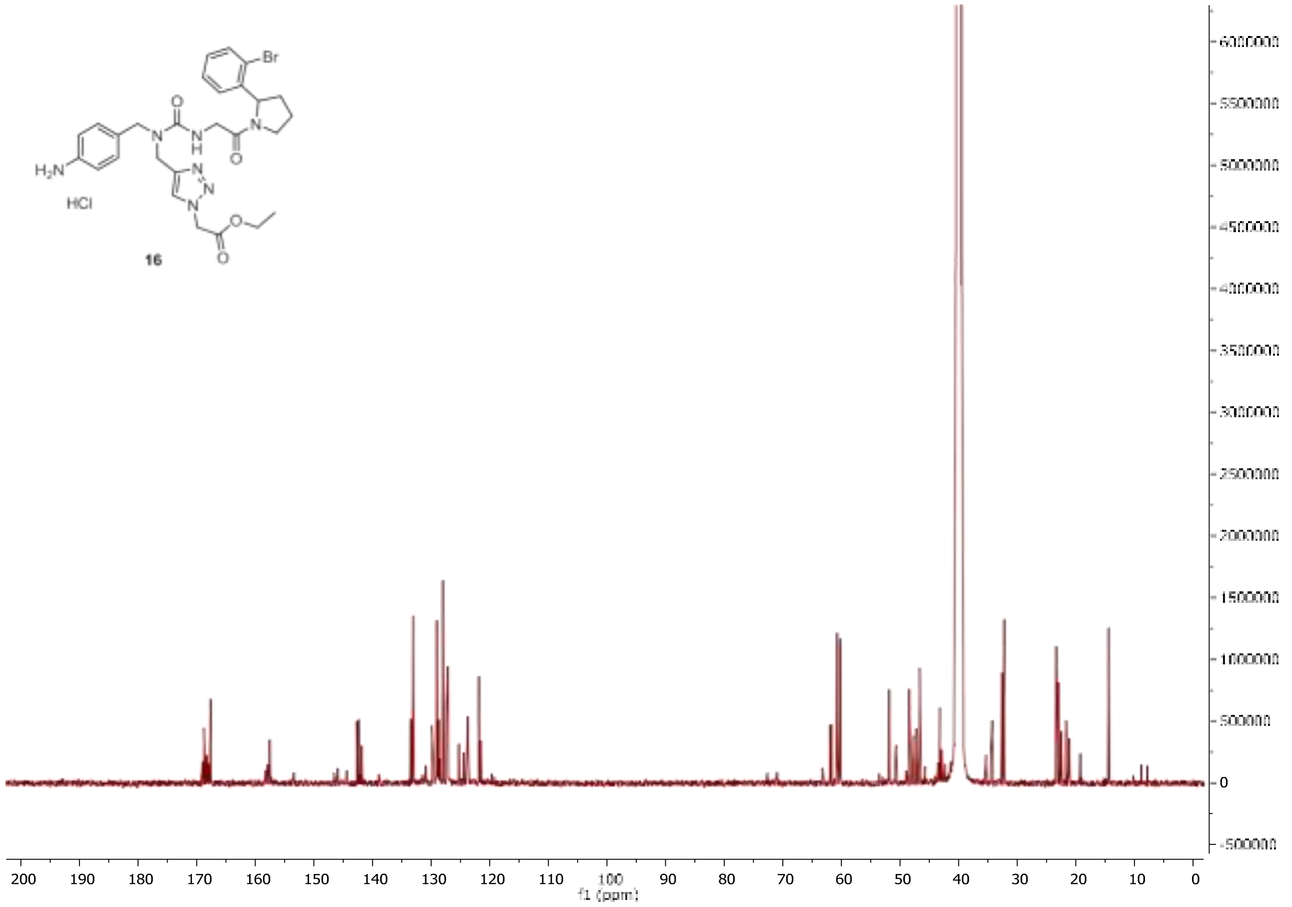

References

1. Ahmed-Belkacem, A.; Colliandre, L.; Ahnou, N.; Nevers, Q.; Gelin, M.; Bessin, Y.; Brillet, R.; Cala, O.; Douguet, D.; Bourguet, W.; Krimm, I.; Pawlotsky, J. M.; Guichou, J. F., Fragment-based discovery of a new family of non-peptidic small-molecule cyclophilin inhibitors with potent antiviral activities. *Nat Commun* **2016,** *7*, 12777.
2. Loeffler, H. H.; Michel, J.; Woods, C., FESetup: Automating Setup for Alchemical Free Energy Simulations. *Journal of Chemical Information and Modeling* **2015,** *55*, 2485-2490.
3. Wang, J.; Wolf, R. M.; Caldwell, J. W.; Kollman, P. A.; Case, D. A., Development and testing of a general Amber force field. *Journal of Computational Chemistry* **2004,** *25*, 1157-1174.
4. Jakalian, A.; Bush, B. L.; Jack, D. B.; Bayly, C. I., Fast, efficient generation of high-quality atomic charges. AM1-BCC model: I. Method. *Journal of computational chemistry* **2000,** *21*, 132-146.
5. Maier, J. A.; Martinez, C.; Kasavajhala, K.; Wickstrom, L.; Hauser, K. E.; Simmerling, C., ff14SB: Improving the Accuracy of Protein Side Chain and Backbone Parameters from ff99SB. *Journal of Chemical Theory and Computation* **2015,** *11*, 3696-3713.
6. Price, D. J.; Brooks, C. L., A modified TIP3P water potential for simulation with Ewald summation. *The Journal of Chemical Physics* **2004,** *121* (20), 10096-10103.
7. Salomon-Ferrer, R.; Götz, A. W.; Poole, D.; Grand, S. L.; Walker, R. C., Routine Microsecond Molecular Dynamics Simulations with AMBER on GPUs. 2. Explicit Solvent Particle Mesh Ewald. *J. Chem. Theory Comput.* **2013,** *9* (9), 3878-3888.
8. Michel, J.; Foloppe, N.; Essex, J. W., Rigorous Free Energy Calculations in Structure-Based Drug Design. *Mol Inform* **2010,** *29* (8-9), 570-8.
9. (a) Eastman, P.; Pande, V. S., OpenMM: A Hardware Independent Framework for Molecular Simulations. *Comput Sci Eng* **2015,** *12* (4), 34-39; (b) Woods, C.; Calabro, C.; Michel, J., www.siremol.org.

10. Andersen, H. C., Molecular dynamics simulations at constant pressure and/or temperature. *The Journal of Chemical Physics* **1980,** *72*, 2384.
11. Barker, J. A.; Watts, R. O., Monte Carlo studies of the dielectric properties of water like models. *Molecular Physics* **1973,** *26* (3), 789-792.
12. Shirts, M. R.; Chodera, J. D., Statistically optimal analysis of samples from multiple equilibrium states. *Journal of Chemical Physics* **2008,** *129*.
13. Mey, A.; Jimenez, J. J.; Michel, J., Impact of domain knowledge on blinded predictions of binding energies by alchemical free energy calculations. *J Comput Aided Mol Des* **2018,** *32* (1), 199-210.
14. Wear, M. A.; Nowicki, M. W.; Blackburn, E. A.; McNae, I. W.; Walkinshaw, M. D., Thermo-kinetic analysis space expansion for cyclophilin-ligand interactions - identification of a new nonpeptide inhibitor using Biacore T200. *FEBS Open Bio* **2017,** *7* (4), 533-549.
15. (a) Tellinghuisen, J., Isothermal titration calorimetry at very low c. *Anal Biochem* **2008,** *373* (2), 395-7; (b) Turnbull, W. B.; Daranas, A. H., On the Value of c: Can Low Affinity Systems Be Studied by Isothermal Titration Calorimetry? *J Am Chem Soc* **2003,** *125* (48), 14859-14866.
16. Zhang, Y.; Zhang, Z., Low-Affinity Binding Determined by Titration Calorimetry Using a High-Affinity Coupling Ligand: A Thermodynamic Study of Ligand Binding to Protein Tyrosine Phosphatase. *Anal Biochem* **1998,** *261*, 139-148.
17. Collaborative Computational Project, N., The CCP4 suite: programs for protein crystallography. *Acta Crystallographica Section D Biological Crystallography* **1994,** *50*, 760-763.
18. (a) Murshudov, G. N.; Vagin, A. A.; Dodson, E. J., Refinement of Macromolecular Structures by the Maximum-Likelihood Method. *Acta Crystallographica Section D Biological Crystallography* **1997,** *53*, 240-255; (b) Murshudov, G. N.; Skubák, P.; Lebedev, A. A.; Pannu, N. S.; Steiner, R. A.; Nicholls, R. A.; Winn, M. D.; Long, F.; Vagin, A. A., REFMAC5 for the refinement of macromolecular crystal structures. *Acta Crystallographica Section D: Biological Crystallography* **2011,** *67*, 355-367; (c) Emsley, P.; Cowtan, K., <i>Coot</i> : model-building tools for molecular graphics. *Acta Crystallographica Section D Biological Crystallography* **2004,** *60*, 2126-2132.
